# Evaluation of Hybridization Capture Versus Amplicon‐Based Methods for Whole‐Exome Sequencing

**DOI:** 10.1002/humu.22825

**Published:** 2015-07-15

**Authors:** Eric Samorodnitsky, Benjamin M. Jewell, Raffi Hagopian, Jharna Miya, Michele R. Wing, Ezra Lyon, Senthilkumar Damodaran, Darshna Bhatt, Julie W. Reeser, Jharna Datta, Sameek Roychowdhury

**Affiliations:** ^1^ Comprehensive Cancer CenterThe Ohio State UniversityColumbusOhio43210; ^2^ Division of Medical OncologyDepartment of Internal MedicineThe Ohio State UniversityColumbusOhio43210; ^3^ Department of PharmacologyThe Ohio State UniversityColumbusOhio43210

**Keywords:** bioinformatics, genomics, exome sequencing, WES, next generation sequencing, NGS

## Abstract

Next‐generation sequencing has aided characterization of genomic variation. While whole‐genome sequencing may capture all possible mutations, whole‐exome sequencing remains cost‐effective and captures most phenotype‐altering mutations. Initial strategies for exome enrichment utilized a hybridization‐based capture approach. Recently, amplicon‐based methods were designed to simplify preparation and utilize smaller DNA inputs. We evaluated two hybridization capture‐based and two amplicon‐based whole‐exome sequencing approaches, utilizing both Illumina and Ion Torrent sequencers, comparing on‐target alignment, uniformity, and variant calling. While the amplicon methods had higher on‐target rates, the hybridization capture‐based approaches demonstrated better uniformity. All methods identified many of the same single‐nucleotide variants, but each amplicon‐based method missed variants detected by the other three methods and reported additional variants discordant with all three other technologies. Many of these potential false positives or negatives appear to result from limited coverage, low variant frequency, vicinity to read starts/ends, or the need for platform‐specific variant calling algorithms. All methods demonstrated effective copy‐number variant calling when evaluated against a single‐nucleotide polymorphism array. This study illustrates some differences between whole‐exome sequencing approaches, highlights the need for selecting appropriate variant calling based on capture method, and will aid laboratories in selecting their preferred approach.

## Introduction

Next‐generation sequencing (NGS) technologies have accelerated efforts to characterize human genomic variation and disease [Metzker, 2010]. However, whole‐genome sequencing remains costly for large‐scale studies, and researchers have instead utilized a whole‐exome sequencing approach that focuses on the expressed exons, which constitute 1% of the genome [Gnirke et al., 2009; Sulonen et al., 2011]. To this end, microarrays have been utilized to enrich for the whole human exome prior to sequencing [Albert et al., 2007; Hodges et al., 2007; Sulonen et al., 2011]. Yet, such array‐mediated capture functions best on DNA fragments around 500‐bp long, thus limiting targeting of closely spaced regions [Hodges et al., 2007; Gnirke et al., 2009], such as human exons [Clamp et al., 2007]. To aid focus on smaller DNA fragments, solution‐based exon capture was developed [Gnirke et al., 2009]. This study aims to compare the merits of several currently available solution‐based exome capture methods in order to aid other research laboratories in their choice of approach.

Whole‐exome methods generally capture from 35 to 70 megabases of target region for sequencing depending on the reference database utilized and inclusion of 3′ or 5′ untranslated regions (UTRs). Several previous studies have examined the strengths and weaknesses of the early whole‐exome capture methods [Asan et al., 2011; Clark et al., 2011; Parla et al., 2011; Sulonen et al., 2011; Chilamakuri et al., 2014]. These have included evaluation of the initial strategies developed, including hybridization capture‐based Agilent's SureSelect, Roche/Nimbelgen's SeqCap, and Illumina's TruSeq and Nextera approaches, all sequenced on an Illumina NGS platform [Clark et al., 2011; Chilamakuri et al., 2014]. To facilitate rapid production of whole‐exome sequencing data, new amplicon‐based methods have been designed that simplify DNA preparation and utilize smaller inputs of DNA. Thus, for researchers seeking to utilize whole‐exome sequencing approaches, there are many choices for capture and sequencing approach.

**Figure 1:**
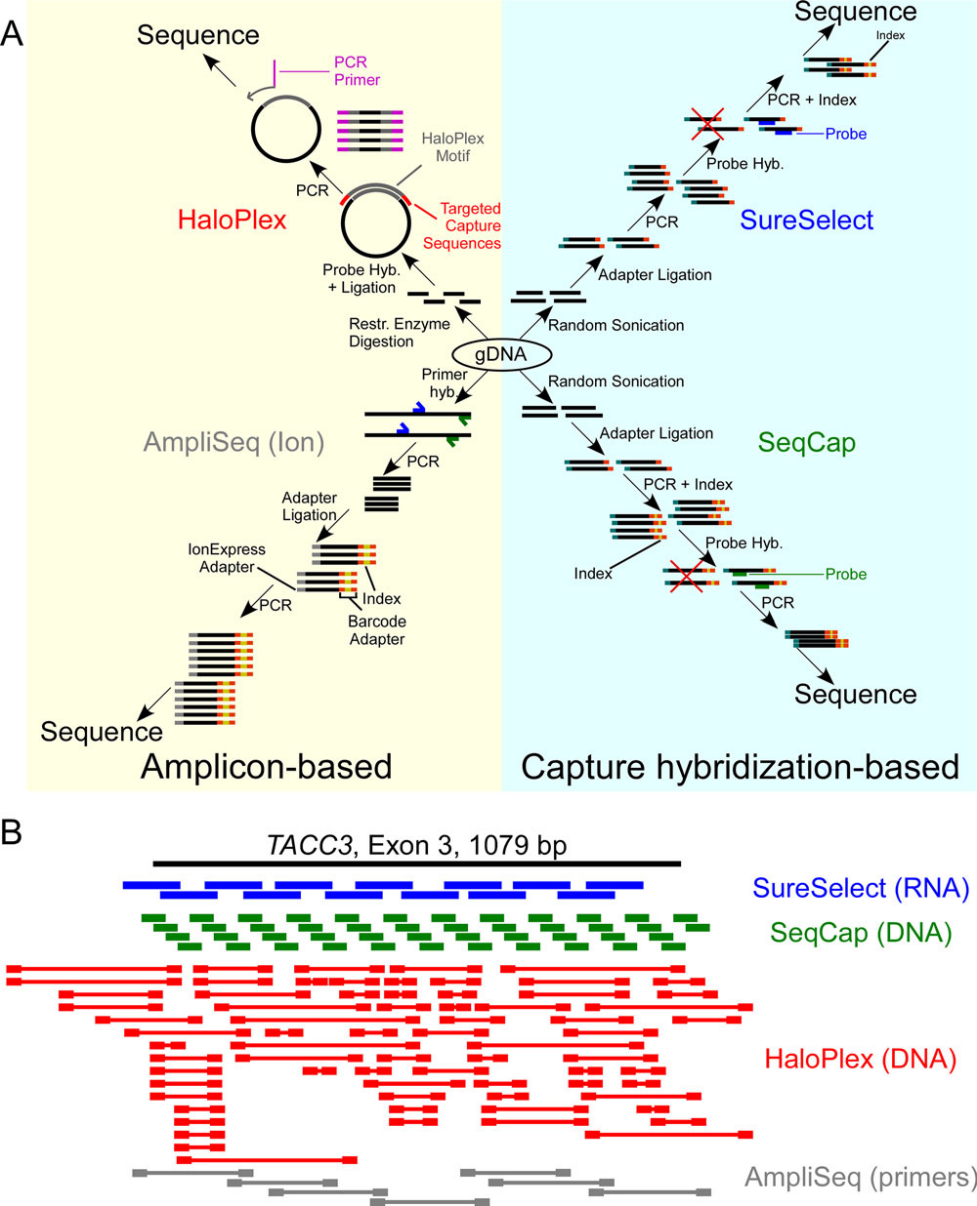
Whole exome capture methods and probe strategy. **A**: Schematic of experimental procedures of all four methods. SureSelect and SeqCap are classified as capture based due to their sonication‐based fragmentation method and the use of oligonucleotides to hybridize and capture target regions, whereas HaloPlex and AmpliSeq are classified as amplicon based due to their use of use of oligonucleotides as PCR primers for amplicons. **B**: Probe or amplicon lay out targeting a particular target exon (*TACC3*, exon 3, chr4:1729435–1730514). The probe layout of SeqCap, in this diagram, is an approximation, since these probe coordinates are not publicly available. SeqCap and SureSelect utilize staggered probes, and Ampliseq uses adjacent amplicons, whereas HaloPlex's probes are complimentary only to sequences near ends of the amplicons, and the middle of the probe is a DNA motif used for HaloPlex‐specific purposes.

Given the significant differences in these novel approaches, we evaluated the newer amplicon‐based and the sonication/hybridization capture‐based approaches for whole‐exome sequencing on two NGS sequencing platforms (Illumina and Ion Torrent) (Fig. 1A). The two capture‐based technologies, SureSelect and SeqCap, shear genomic DNA using high‐frequency sounds waves (sonication) creating randomly sized DNA fragments. Then, synthetic oligonucleotides are hybridized to regions of interest in solution and captured through magnetic beads. One of the amplicon methods, HaloPlex, however, fragments genomic DNA with restriction enzymes, then uses probes complimentary to the 5′‐ and 3′‐ends of each fragment for the creation of a targeted amplicon; this is followed by PCR primer annealing and PCR amplification. The final method we evaluated, Ion Torrent's Ion AmpliSeq, amplifies target regions with PCR primers, followed by sequencing on the Ion Proton^TM^ System. We evaluated all technologies for normalized metrics including on‐target alignment, coverage uniformity, and GC bias. Furthermore, unlike previous comparison studies, we provide an in‐depth concordance assessment for detection of single‐nucleotide variants (SNVs) using three variant callers. Lastly, we assessed each technology's ability to call copy‐number variants (CNVs).

## Materials and Methods

### Cell Lines

We obtained BT‐20 breast cancer cell line (with *PIK3CA* NM_006218.2:c.1616C>G p.P539R and NM_006218.2:c.3140A>G p.H1047R mutations), MCF‐7 breast cancer cell line (with a *PIK3CA* NM_006218.2:c.1633G>A p.E545K mutation), breast cancer cell line HCC‐2218 (with *ERBB2* amplification), and HCC‐2218's matching B‐lymphoblastoid cell line, HCC‐2218BL, from the American Type Culture Collection (ATCC, Manassas, VA) (Supp. Table S1). DNA was extracted from log phase growing cell lines using the DNeasy Blood and Tissue Kit (Qiagen, Valencia, CA). DNA was quantitated using the Qubit dsDNA HS Assay Kit with the Qubit 2.0 Fluorometer (Invitrogen, Carlsbad, CA) and the NanoDrop 2000C (Thermo Scientific, Waltham, MA) (optical density ratios were 260:280 = 1.8–2.0, 260:230 = 2.0–2.2). Genomic DNA ScreenTapes were used on the TapeStation 2200 System (Agilent Technologies, Santa Clara, CA) to evaluate DNA size and quality. All four cell lines tested negative for mycoplasma and were authenticated (DNA Diagnostics Center, Cincinnati, OH).

### SureSelect^XT^ Human All Exon V4+UTR's

We used 3 μg of each cell line's genomic DNA diluted in 1× TE Buffer (pH 8.0) and sheared to a target peak size of 150–200 bp using the Covaris S220 focused‐ultrasonicator (Covaris, Woburn, MA) according to the manufacturer's recommendations. Library preparation and exome capture were performed using the SureSelect^XT^ Human All Exon V4+UTR's capture baits as described in Agilent's SureSelect^XT^ Target Enrichment System for Illumina Paired‐End Sequencing Library Protocol (version 1.5) without modification. We performed 11 cycles of PCR for amplification of the postcapture exome libraries and validated the quality of each library using Agilent's High Sensitivity D1K ScreenTapes on the TapeStation 2200 system.

### SeqCap EZ Human Exome V3.0

We used 1.1 μg of each cell line's genomic DNA diluted in nuclease‐free water and sheared to a target peak size of 250–300 bp using the Covaris S220 focused‐ultrasonicator according to the manufacturer's specifications. Whole‐genome libraries were prepared using the Illumina TruSeq DNA Kit and Sample Preparation Guide without modification. Exome capture was executed by following Roche's SeqCap EZ Library Guide (version 4.1) with the SeqCap EZ Human Exome Library V3.0 Kit. We performed 14 cycles of PCR to amplify the postcapture exome libraries and assessed library quality using Agilent's D1K Screentapes (regular sensitivity) on the TapeStation 2200 system.

### HaloPlex Exome

We diluted 225 ng of genomic DNA from each cell line in nuclease‐free water and fragmented the DNA in eight separate restriction enzyme digestion reactions. The Haloplex Exome Target Enrichment System Protocol (Version A) was followed without modification to perform the library preparation and exome capture using the HaloPlex Exome capture baits. We assessed library quality using Agilent's High Sensitivity D1K ScreenTapes on the TapeStation 2200 system.

### Ion Ampliseq Exome

We submitted 250 ng of each cell line's genomic DNA to the Roswell Park Cancer Institute's Genomics Shared Resource (GSR). As an Ion Ampliseq Exome Certified Service Provider, the GSR prepared and sequenced each AmpliSeq Exome library according to the manufacturer's specifications on the Ion Proton^TM^ System on a P1.1.17 chip. Each library was sequenced on a separate chip.

### Sequencing of Libraries

We prepared indexed SureSelect, SeqCap, and HaloPlex whole‐exome libraries and sent them for 100‐bp paired‐end sequencing (2 × 100 bp) on an Illumina HiSeq 2000 at Beijing Genomics Institute (BGI, Beijing, China). For SureSelect and SeqCap, all four libraries were pooled and sequenced in one lane and two lanes, respectively. For HaloPlex, BT‐20 and MCF‐7 were pooled and sequenced on one lane, with HCC‐2218BL and HCC‐2218 in a separate lane. The AmpliSeq libraries were single‐end sequenced on an Ion Proton^TM^ System at Roswell Park Cancer Institute.

### Single‐Nucleotide Polymorphism Array

We submitted DNA isolated from HCC‐2218BL and HCC‐2218 to Case Western Reserve University's Genomic Sequencing Core to use the Affymetrix Genome‐Wide Human SNP Array 6.0 (Affymetrix, Santa Clara, CA) to determine copy number variation. The Core utilized the Affymetrix Genome‐Wide Human SNP Array 6.0 method for sample preparation. Briefly, 500 ng of genomic DNA was digested employing the Nsp1 and Sty1 restriction enzymes and then ligated to adapters. These adapter‐bound fragments were then amplified, fragmented, labeled, and hybridized to the SNP Array 6.0.

### Alignment

For SureSelect and SeqCap paired‐end data, BGI removed reads with adapters, reads with 5% or more unknown bases, and reads with 50% or more bases with quality score less than or equal to 10, using their own custom algorithm called SOAPnuke. Manual inspection in Integrative Genomics Viewer [Robinson et al., 2011] confirmed this. Because HaloPlex utilizes different adapters from the standard Illumina TruSeq adapters, we used Agilent's proprietary software SureCall‐2.1.1.13 under the default parameters to trim adapters from paired‐end HaloPlex libraries. Raw sequencing reads were aligned to the human genome (hg19) utilizing the Burrows‐Wheeler Aligner (BWA‐0.6.2) [Li and Durbin, 2010] employing the default parameters. Resultant SAI‐files were consolidated and converted to Sequence Alignment/MAP (SAM) using the SAMtools‐0.1.18 [Li et al., 2009aa] “sampe” command, the output SAM files were changed to Binary Alignment/MAP (BAM) using the SAMtools “view” command, and the latter were sorted by chromosome and position using the SAMtools “sort” command. After reads were aligned, duplicate reads were removed from SureSelect and SeqCap libraries using Picard‐1.84's (http://picard.sourceforge.net/) “MarkDuplicates” command. In accordance with manufacturer's instructions, duplicates were not removed from HaloPlex data. Afterward, regardless of whether the technology required duplicate removal, we realigned reads around known indels in dbSNP file hg19 snp137 [Sherry et al., 2001] using the Genome Analysis Toolkit‐2.4.7 (GATK) [McKenna et al., 2010] employing the “RealignerTargetCreator” and “IndelRealigner” commands. Due to high‐quality scores (in our experience, these commands failed when they found an alignment score greater than or equal to 61), realignment commands included the additional “fixMisencodedQuals” flag. Following realignment, Picard's “FixMateInformation” command under the default parameters was used. Next, quality scores were recalibrated using GATK's “BaseRecalibrator” and “PrintReads” commands under the default parameters. Additionally, the resulting BAM files were sorted by name using the SAMtools “sort” function to generate name‐sorted BAM files.

Note that AmpliSeq data were processed differently from SureSelect, SeqCap, and HaloPlex libraries. Roswell Park Cancer Institute's Genomics Shared Resource used the Torrent Mapping Alignment Program (TMAP)‐4.0.6 to trim adapters from single‐end AmpliSeq data, align reads to human genome (hg19), and realign reads around SNV candidates; however, when we performed our downsampling analysis (which will be described later), we used TMAP‐3.0.1, which was the latest version available for download (https://github.com/iontorrent/TS/tree/master/Analysis/TMAP).

Since each exome capture has a different target region, we assessed on‐target performance for each method's respective target region. For all four technologies, following mapping and postprocessing, we calculated alignment statistics using BEDTools‐2.17.0 [Quinlan and Hall, 2010]. Using this software, three different alignment percentages were calculated: percent of sequenced fragments that aligned to the human genome (hg19), percent of total sequenced fragments that mapped to target regions of the respective technology, and percent of hg19‐aligned fragments that mapped to target regions of the respective technology. The aforementioned alignment statistics were calculated for SureSelect, SeqCap, and HaloPlex data using the final BAM file (i.e., after duplicate removal, realignment around indels, quality score recalibration, and name sorting). However, for AmpliSeq, these statistics were calculated using the resulting BAM file from mapping raw FASTQ reads to hg19 and realigning around SNV candidates (the Ion Torrent Suite's standard analysis, for AmpliSeq data, does not include duplicate removal, realignment around known indels, or quality score recalibration, which is included in our pipeline for analysis of Illumina data). For all four technologies, we used BEDTools's “bamtobed” command to convert the respective BAM files to BEDPE format for SureSelect, SeqCap, and HaloPlex, and BED format for AmpliSeq. These BEDPE/BED files were used to calculate the percentage of FASTQ fragments that aligned to hg19. Next, to calculate alignment rate to targeted regions, for SureSelect, SeqCap, and HaloPlex, we used the BEDTools “pairtobed” command to record the intersection between each of the previously mentioned BEDPE files and their respective technology's target regions. For single‐end AmpliSeq data, the BEDTools “intersect” command was used instead. These commands consider a fragment “on‐target” if at least one base from the fragment intersects a targeted region. To calculate the portion of targeted bases covered at various sequencing depths, we utilized mpileup files generated by SAMtools on the final BAM files.

### SNV Calling

For SureSelect, SeqCap, and HaloPlex, final BAM files were used to generate mpileup files using the SAMtools “mpileup” command, accepting reads with a minimum mapping quality of 10. Using these mpileup files, SNVs were called using VarScan2's (v.2.3.3) [Koboldt et al., 2012] “mpileup2snp” function under the default parameters. We also called SNVs on SureSelect, SeqCap, HaloPlex data using GATK's HaplotypeCaller [McKenna et al., 2010] and MuTect‐1.1.4 [Cibulskis et al., 2013], both under the default parameters, inputting each sample's final BAM files and the respective technology's target regions BED file. In addition, for HaloPlex data, we used Agilent's SureCall‐2.1.1.13 software (Agilent Technologies, Santa Clara, CA), a tool for HaloPlex analysis, under the default parameters. We annotated mutations using ANNOVAR (version 525) [Wang et al., 2010] to remove intronic and synonymous SNVs and to match each SNV with known genes and their associated amino acid changes, stopgain, or stoploss. Lastly, we filtered for SNVs that fell inside each technology's target regions. Note that for SNVs called by GATK, we omitted SNVs that were reported with another SNV or indel at the same position for simplicity.

Due to unique requirements for variant calling for Ion Torrent data related to homopolymer sequencing errors [Bragg et al., 2013], we utilized the company's Ion Torrent Suite 4.0.2 to calculate variants from AmpliSeq data (see Supp. Table S2 for parameters). Note that for SNVs called by the Ion Torrent Suite, we disregarded SNVs that were reported with a deletion at the same position. This indicates that Ion Torrent could miss a true SNV due to deletion calls.

Venn diagrams were prepared using Venny (http://bioinfogp.cnb.csic.es/tools/venny/).

### Indel Calling

For SureSelect, SeqCap, and HaloPlex, mpileup files were used to call indels using VarScan2's “mpileup2indel” command under the default parameters. We also called indels on SureSelect, SeqCap, and HaloPlex using GATK's HaploTypeCaller (same parameters as SNV calling above; also, as with SNVs, we omitted indels that were reported with another indel at the same position). Lastly, we used Pindel v0.2.5a8 [Ye et al., 2009] on SureSelect, SeqCap, and Haloplex libraries under the default parameters, inputting each sample's final BAM file; we disregarded indels reported with another mutation at the same position). As with SNV calling above, Ion Torrent Suite was used to call indels on AmpliSeq libraries. For all technologies, we annotated indels using ANNOVAR (version 525) to remove intronic indels and to filter for frameshift insertions, frameshift deletions, nonframeshift insertions, and nonframeshift deletions. To compare concordance, we looked only at indels whose 5′‐end fell within commonly targeted bases.

### CNV Calling

For all four technologies, we called CNVs on the HCC‐2218 cell line using the HCC‐2218BL cell line as a matched normal control. Using the final BAMs from both samples, we generated two‐sample mpileup files using the SAMtools “mpileup” function. We used VarScan2's “copynumber” command to call CNVs for SureSelect, SeqCap, and HaloPlex under the default parameters, except for the data ratio flag. We defined the data ratio flag by dividing the number of reads in the “reference” column of the mpileup file by the number of reads in the “tumor” column of the mpileup file (the data ratio flag is used by VarScan2 to account for differences in global genomic sequencing depth between the tumors and matched normal samples when calling CNVs). For AmpliSeq data, when “copynumber” was run, we used the data ratio flag, a minimum base quality of 1, and a minimum mapping quality of 1, because base qualities were generally lower on the Ion Proton^TM^ than the Illumina HiSeq [Loman et al., 2012]. The CNV output included chromosomal segments and their respective log_2_‐CNV ratios. Then, using the BEDTools “intersect” command, we filtered the CNV calls to regions inside each technology's respective target region.

We compared CNV calls from sequencing data with SNP 6.0 array data utilizing Affymetrix's Genotyping Console^TM^ Software‐4.1.4.840, according to manufacturer's instructions (Affymetrix). Using SNP array data, we generated log_2_‐CNV ratios at individual base positions for HCC‐2218 and HCC‐2218BL, each. To directly compare CNVs between HCC‐2218 and HCC‐2218BL, we used the following formula:

log 2 HCC −2218 HCC −2218 BL = log 2 HCC −2218− log 2 HCC −2218 BL

We assessed correlation between sequencing and SNP array‐derived log_2_–CNV ratios. For each SNP array base position and its corresponding CNV ratio, if any technology's target‐filtered CNV file had a CNV ratio at the corresponding position, we paired the SNP array and the sequencing CNV ratios. Before calculating correlation between the SNP array and sequencing CNV ratios, we filtered out regions whose sequencing CNV ratio fell between −0.5 and 0.5 to exclude possibly copy‐neutral regions or false positives. Then, we calculated the correlation of log_2_–CNV ratios between the SNP array and each sequencing technology.

### Statistical Tests

For all data where statistical significance was computed, we used the two‐sided Mann–Whitney *U*‐test. *P* values for all Mann–Whitney *U*‐tests are in Supp. Table S3.

## Results

### Library Construction Varies Between Technologies

We examined the abilities of four available technologies (Fig. 1A), Agilent's SureSelect, Roche/Nimblegen's SeqCap EZ Exome, Agilent's HaloPlex Exome, and Ion Torrent's Ion AmpliSeq Exome to capture whole exomes for NGS (Table 1) using four well‐annotated cell lines (Supp. Table S1 for sequencing statistics). The established SureSelect and SeqCap methods utilize sonication to fragment genomic DNA to generate unique fragments. In contrast, the newer HaloPlex and AmpliSeq use novel means to create inserts and condense some steps for library construction to shorten sample preparation time. HaloPlex generates fragments through restriction enzyme digests and uses nontiled DNA probes complementary to known restriction enzyme digestion sites. These probes are designed to circularize the digested fragment of interest to enable PCR amplification (Fig. 1A). Subsequently, HaloPlex combines adapter ligation with PCR amplification (in Fig. 1B, the PCR primers in HaloPlex are partially composed of adapters). Finally, AmpliSeq uses multiple sets of PCR primers, rather than probes, to amplify regions of interest, but similar to SureSelect and SeqCap, target regions are tiled. These methods have several underlying differences in probe strategy, including different probe sizes (Table 1), composition (DNA or RNA), density, and layouts (Fig. 1B). SureSelect utilizes RNA probes, whereas the remaining approaches use DNA probes or primers. SureSelect and SeqCap (and AmpliSeq, for longer exons) each employ a tiled probe layout (Fig. 1B).

**Table 1:** Technical Specifications of Whole‐Exome Capture Methods

	SureSelect^a^ (Agilent)	SeqCap^a^ (Nimblegen)	HaloPlex^a^ (Agilent)	AmpliSeq^a^ (Life Technologies)
Capture method	Hybridization	Hybridization	Amplicon	Amplicon
Platform(s) available	Illumina or Ion Torrent	Illumina or Ion Torrent	Illumina or Ion Torrent	Ion Torrent
Targeting method	RNA linear probes	DNA linear probes	DNA molecular inversion probes	PCR primers
Length of probes/amplicons (bp)^b^	120	55–105	161 ± 75	256 ± 14
Number of probes/amplicons	7.89 × 10^5^	2.10 × 10^6^	2.49 × 10^6^	3.17 × 10^6^
Target size (Mb)	70.4	63.6	38.5	57.7
Overall cost of sequencing per reaction (USD)	1,528.98	1,424.79	1,545.67	1,200.00

a Versions of technologies: SureSelect^XT^ Human All Exon V4+UTR's, SeqCap EZ Human Exome V3.0, HaloPlex Exome, Ion AmpliSeq Exome.

b Note that the length of amplicons for HaloPlex and AmpliSeq are denoted as median ± median absolute deviation.

### Examination of Library and Sequencing Measurements

**Figure 2:**
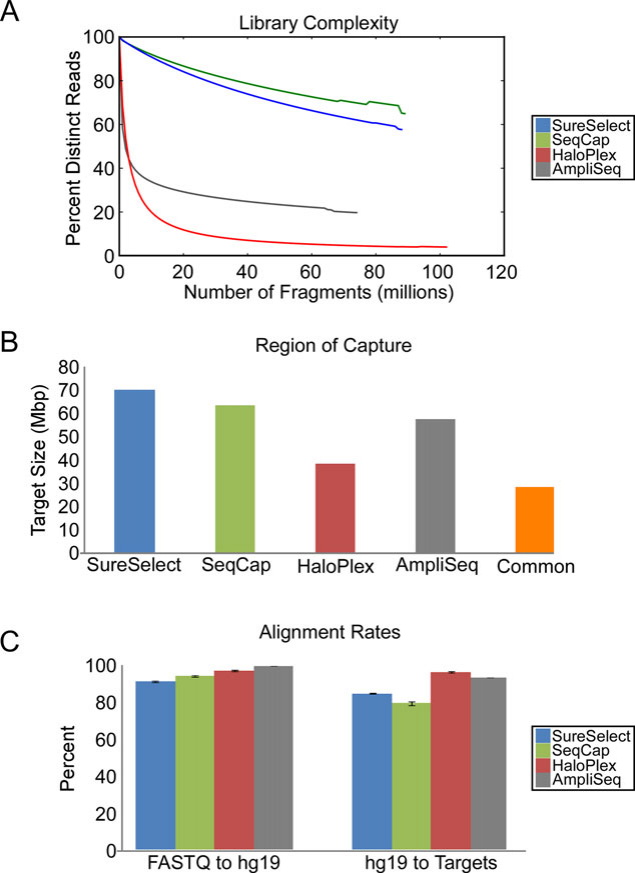
Assessment of raw library sequencing metrics. **A**: Percent molecular complexity is defined as the number of unique reads divided by the number of total reads in a sample, multiplied by 100% [Daley and Smith, 2013] as a function of millions of reads sampled. **B**: Whole‐exome regions targeted by each technology and the region in common between all technologies. **C**: Mapping statistics from raw sequencing files to hg19 and alignment from hg19 to target regions after postprocessing.

We first assessed basic library metrics. We applied Preseq [Daley and Smith, 2013] to calculate the complexity of each library (Fig. 2A). Using input BAM or BED files, Preseq computes the expected number of distinct sequencing reads as a function of total reads. The hybridization‐based methods showed significantly higher complexity than the amplicon‐based methods (*P* = 0.03). Next, we sought to assess each method's ability to capture the whole exome. While each technology attempts to target all coding exons, they also rely on different combinations of databases (i.e., Vega, Gencode, Ensembl, CCDS, etc.) for target region compilation; consequently, target region size varies between technologies (Fig. 2B). For example, SureSelect targets UTRs and SeqCap targets miRNA exons. We compared the alignment rates with targeted regions between the technologies by examining alignment from raw sequencing files to the reference genome (hg19) and from aligned fragments to each technology's respective target regions (Supp. Table S4; Fig. 2C) (median ± median absolute deviation, unless otherwise stated). We defined on‐target if at least one base of either the 5′‐end of 3′‐end of a fragment aligned to the target region. All technologies aligned greater than 90% of their raw fragments to the genome, whereas the amplicon‐based methods, HaloPlex and AmpliSeq, aligned the highest percentage of their mapped fragments to target regions (*P* = 0.03) (94.67% and 93.68%, respectively).

### Uniformity of Sequencing over Target Areas

**Figure 3:**
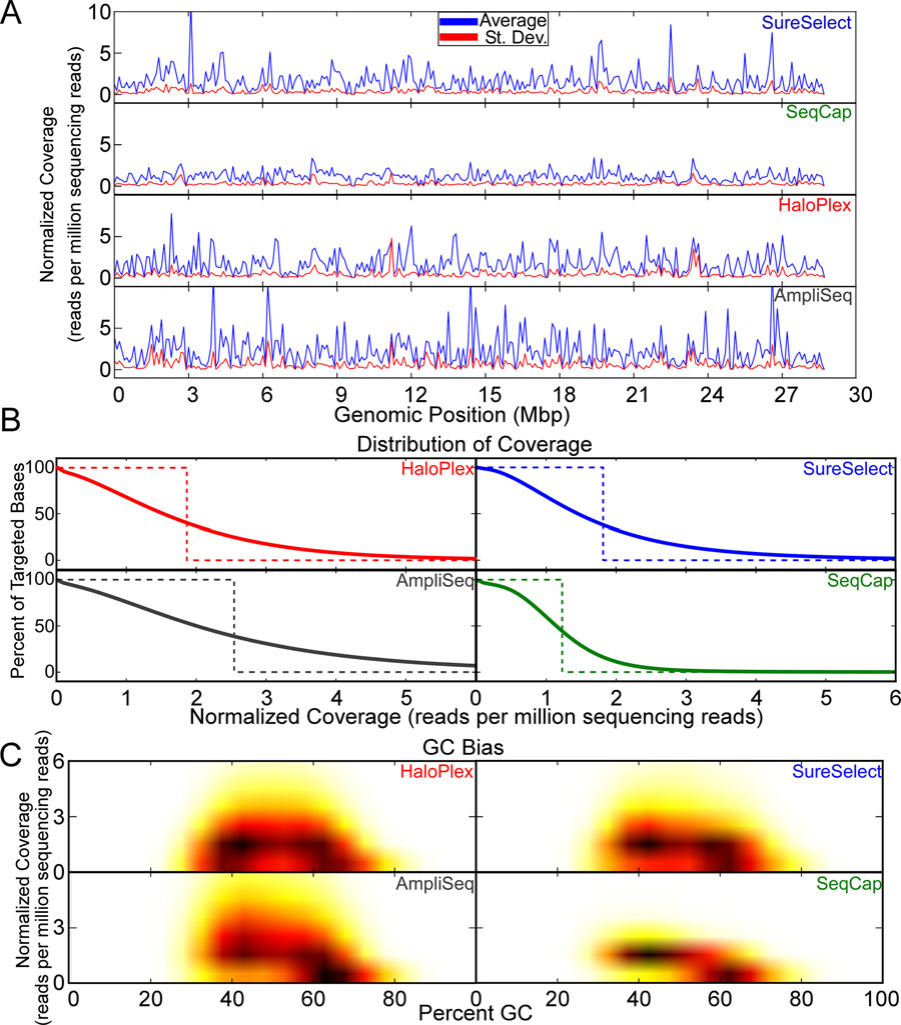
Assessment of sequencing depth in commonly targeted regions. **A**: Normalized coverage (reads per million sequencing fragments) (*y*‐axis) for all bases in commonly targeted regions. The average (blue) and standard deviation (red) for all genomic positions in commonly targeted regions are shown. **B**: Percentage of commonly targeted bases that had various minimum average coverage (solid lines). An “ideal” curve is plotted for each technology that assumes that all sequencing covers targeted bases uniformly. Because different technologies have different average normalized coverage (partially due to differences in capture size), ideal curves will differ between technologies. **C**: Normalized coverage was plotted against percent GC in 100‐bp windows for all mutually targeted bases for each technology. Darker colors and brighter colors indicate denser and lighter clusters of points, respectively.

In addition to on‐target sequencing rate, another important element is the distribution of sequencing or sequencing uniformity across the target region. We computed average normalized sequencing depth (defined as read count per million sequencing reads) in bases common to all technologies (Fig. 3A). While both amplicon‐based methods showed higher average coverage than hybridization capture‐based technologies (*P* < 10^−323^), both amplicon‐based methods also exhibited a higher standard deviation of coverage than did the hybridization capture‐based methods over commonly targeted regions (Supp. Table S5). This analysis was repeated for targeted regions of each respective technology and the results were similar (Supp. Table S5 and Supp. Fig. S1). This suggests that hybridization capture‐based methods have better sequencing uniformity than amplicon‐based methods.

To further characterize sequencing uniformity, we plotted the quantity of mutually targeted bases with various minimum average normalized coverage (solid curves in Fig. 3B; note that UTRs and miRNAs were included in SureSelect's and SeqCap's target regions, respectively, possibly lowering their overall coverage). Next, we defined a scenario, where all technologies sequenced all bases uniformly and to exemplify this, we plotted an “ideal curve” (dotted curves in Fig. 3B). In these ideal curves, each technology sequences every base position at a depth equal to its average normalized coverage in the commonly targeted region. The capture‐based methods deviated less from their respective ideal curves than did the amplicon‐based methods (*P* < 10^−323^) (Supp. Table S6), and this was true when we assessed each technology's own target region (Supp. Fig. S2 and Supp. Table S6). This gives further evidence that sequencing uniformity is greater for capture‐based than amplicon‐based methods.

### Effect of GC Content on Capture Performance

While no method for exome capture exhibits perfect uniformity, we sought to understand the degree to which GC bias influenced uniformity for each technology. Previous reports have demonstrated that regions with high or low GC content may affect probe hybridization and PCR bias [Asan et al., 2011; Clark et al., 2011]. We plotted normalized coverage in commonly targeted regions as a function of GC content in regions commonly targeted by all technologies (Fig. 3C). All capture methods were relatively evenly distributed with respect to GC content. We observed a similar trend when we plotted normalized coverage against GC content in regions targeted specifically by each technology (Supp. Fig. S3).

### Desired Sequencing Depth Calculator as a Function of Reads

**Figure 4:**
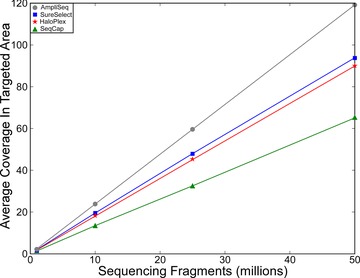
Number of sequencing fragments required for desired depth of coverage. Average coverage in target regions as a function of number of sequenced fragments. Note that SureSelect, SeqCap, and HaloPlex libraries were all paired‐end sequenced as 2 × 100 bp, whereas AmpliSeq was single‐end sequenced with variable read lengths.

For investigators planning whole‐exome sequencing projects, a common consideration is the average depth of sequencing desired. Considering how each technology has variable performance, we constructed a simple graph that allows comparison of how many sequencing reads or sequenced bases are necessary to obtain a specified average depth. Therefore, we randomly downsampled FASTQ files to 1 million, 10 million, 25 million, and 50 million paired‐end fragments (or single‐end for AmpliSeq fragments) and determined the average coverage in each technology's targeted area requiring a minimum mapping quality of 10 for each read (Fig. 4). The amplicon‐based sequencing methods and hybridization capture‐based methods showed no significant difference in sequencing depth as a function of fragments sampled (*P* = 0.70). Even though the sequencing depth is not statistically significant between methods, this analysis enables investigators to anticipate the approximate quantity of reads that should be sequenced in order to achieve the desired depth for a given capture method. However, this graph does not consider differences in sequencing uniformity across the target region or differences in target size (Fig. 3).

### Concordance of Technologies in SNV Calling

**Figure 5:**
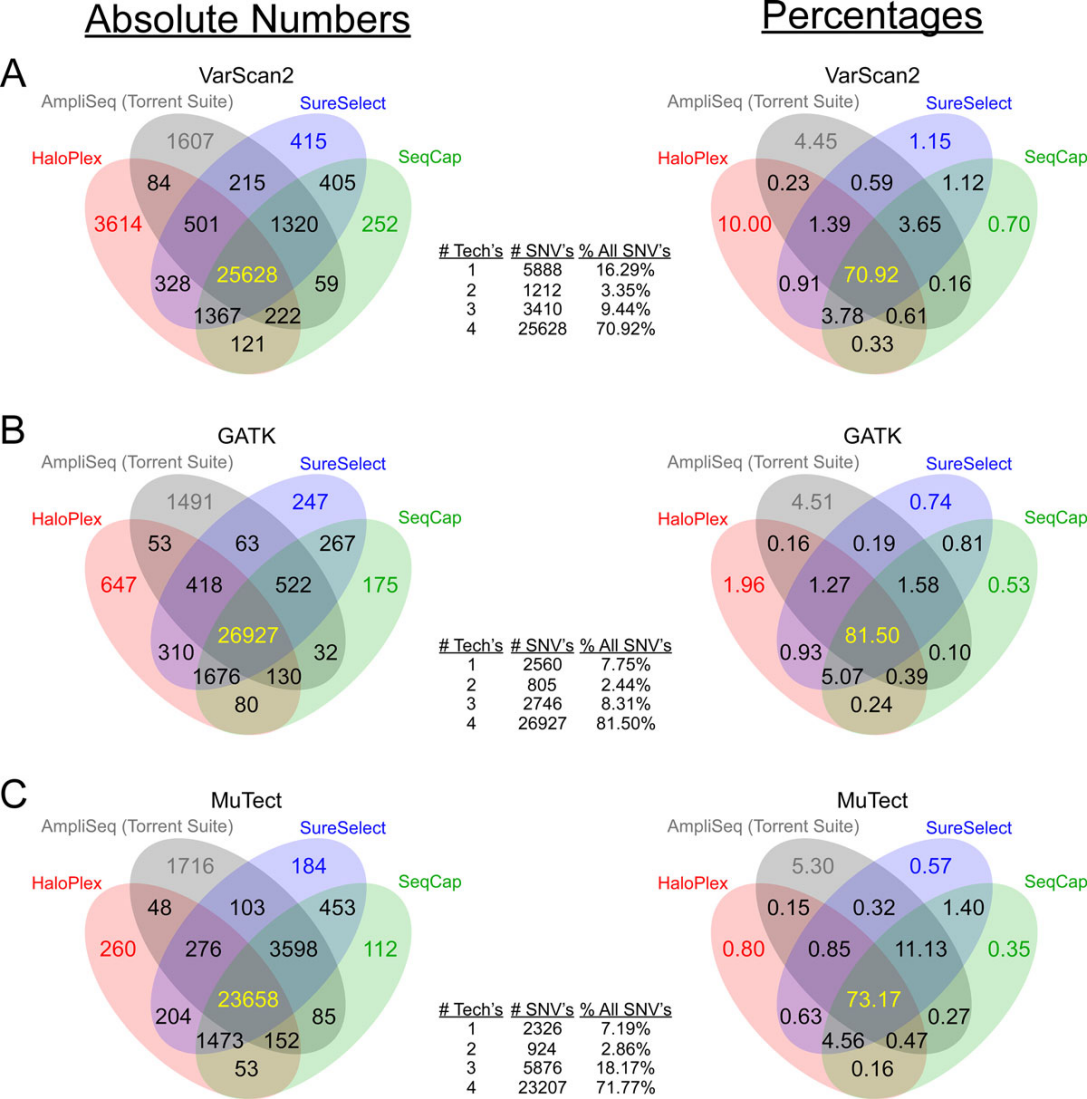
Concordance of SNV calling among technologies. Utilizing three variant callers, **A**: Varscan2; **B**: GATK; and **C**: MuTect, Venn diagrams compare absolute numbers of nonsynonymous SNVs (left) and percentage of the total number of nonsynonymous SNVs (right) in commonly targeted regions. Tables in the center count the number and percentage of concordant SNVs called by strictly one, two, three, or all four technologies.

For all four samples sequenced with SureSelect, SeqCap, and HaloPlex, we assessed single‐sample SNVs using three variant callers, VarScan2 [Koboldt et al., 2012] (Supp. Tables S7–S9), GATK [McKenna et al., 2010] (Supp. Tables S10–S12), and MuTect [Cibulskis et al., 2013] (Supp. Tables S13–S15), whereas we used only the vendor designed Torrent Variant Caller for AmpliSeq data (Supp. Table S16) (see published comparisons of variant callers [Liu et al., 2013; Roberts et al., 2013; Wang et al., 2013]). To reduce the possibility of false‐positive SNVs, we limited our analysis to SNVs with variant frequency of at least 20%. We compared nonsynonymous SNVs identified in regions targeted by all four technologies and found that 70%–82% of all SNVs were called by all four technologies, depending on the variant caller (Fig. 5; we observed similar concordance pattern when we evaluated both nonsynonymous and synonymous SNVs in Supp. Fig. S4). Of all discordant variants regardless of caller (i.e., variants missed by at least one technology), most were either called or missed solely by one amplicon‐based technology (Supp. Table S17).

Because amplicon‐based technologies missed the most SNVs, we investigated possible reasons for SNV discordance in the amplicon‐based data when VarScan2 and MuTect were used to call variants (we did not investigate discordance when GATK was used, because HaloPlex did not have many outliers using this variant caller). First, we investigated SNVs missed by HaloPlex that all other technologies called with VarScan2. The majority of such SNVs in the HaloPlex data (Fig. 5A) (840 of 1,320, 63.64%) fell below VarScan2's minimum coverage of eight total reads for consideration (regardless of base quality). For the 480 SNVs that did meet minimum coverage in HaloPlex, we found other reasons for discordance: 106 did not have at least eight reads whose base quality at the respective position was at least 15, as required by VarScan2; 252 showed variant frequency less than the minimum cutoff of 20%; and 120 SNVs had variant frequency greater than 20%, but were rejected by VarScan2 due to insufficient *P* value (default value = 0.01) (see Supp. Table S18 for variant frequency of these 480 SNVs in SureSelect and SeqCap). Lastly, HaloPlex called a different alternate base from the other technologies on two SNVs.

In addition to false negatives called in HaloPlex data, we assessed potential false positives (3,614 SNVs). Of these SNVs, we investigated the 50 that were covered the highest in HaloPlex (Supp. Table S19). We found that 17 were likely false positives caused by DNA motifs associated with Illumina sequencing errors [Meacham et al., 2011; Nakamura et al., 2011] (Supp. Fig. S5). For the remaining 33 SNVs, we found the variant frequency in HaloPlex to be broadly distributed between 20% and 50% and five variants with at least 50% variant frequency. In SureSelect and SeqCap, however, 24 of these SNVs had variant frequency less than 1%, indicating likely false positives in HaloPlex. Three had SureSelect and SeqCap variant frequency between 11% and 20%, indicating possible low‐variant frequency true positives. For the rest of the variants, frequency spanned between 1% and 11% for SureSelect and SeqCap. Lastly, we Sanger sequenced four SNVs that were nominated by all three variant callers in HaloPlex, but never in SureSelect, SeqCap, or Ampliseq. Three of these SNVs were determined to be false positives by HaloPlex, but one was indeed a true variant. The latter was missed by SureSelect and SeqCap, because the SNV frequency was less than our cutoff of 20% and missed by AmpliSeq due to lack of any variant‐supporting reads.

Next, we considered potential false negatives in HaloPlex when using MuTect (Fig. 5C). We found that, unlike VarScan2, raw coverage was not a major factor for discordance in this case. Instead, 3,097 of 3,598 such SNVs were rejected, because MuTect considered these variants “clustered position” [Cibulskis et al., 2013] (Supp. Figs. S6 and S7). If variant‐supporting bases are grouped near the start or end of a read, MuTect considers such clustering as possible low‐quality misalignments [Li et al., 2009b] and will, thus, reject this variant. This quality filtering was problematic for HaloPlex, because fragments are reproducibly generated by restriction enzymes, which cut only recognized sequences, as opposed to other technologies where fragments are generated randomly. This suggests that HaloPlex sequencing may be incompatible with variant calling using MuTect, because of this “clustered position” filter.

We next investigated potential false negatives and positives in AmpliSeq data (Fig. 5). AmpliSeq data have been optimized to run only on the Ion Proton^TM^ System, thus requiring its own separate analysis pipeline and variant caller (see *Material and Methods*). To address reasons for AmpliSeq's discordance, we first investigated the 1,367 SNVs missed only by AmpliSeq when compared with VarScan2 variant calls in the other technologies (Fig. 5A). Using SAMtools's mpileup function to approximate the AmpliSeq coverage of these SNVs, we found that 458 (33.50% of 1,367) failed to gather enough coverage of five reads needed for the Ion Torrent Suite parameters. Next, we investigated 50 of the 1,367 SNVs with highest AmpliSeq coverage (Supp. Table S19). Twenty‐two SNVs had variant frequency less than our minimum cutoff 20%. Another 19 were missed due to Ion sequencing errors caused by nearby homopolymers (Supp. Fig. S8) [Bragg et al., 2013]. The rest were missed due to strand bias (Supp. Fig. S9) and some low‐quality variant‐supporting bases, possibly causing the Ion Torrent Suite to reject the SNVs. Lastly, one SNV was rejected because of C‐insertion supporting reads, without an adjacent C‐nucleotide, which has been reported previously [Bragg et al., 2013].

Next, we examined potential false positives in AmpliSeq's nomination of 1,607 SNVs that were unsupported by any other technology, when VarScan2 was used as the variant caller (Fig. 5A). We looked closely at the 50 SNVs that were covered the highest by AmpliSeq (Supp. Table S19). We found that 19 SNVs were false positives as a result of Ion Torrent platform sequencing errors due to homopolymer regions [Bragg et al., 2013] (Supp. Fig. S10). Four others had AmpliSeq variant frequency slightly above our 20% cutoff (20%–25%), but 0%–2% variant frequency in the other technologies. One variant's frequency was 22.07% in AmpliSeq, but its frequency was 15%–17% for the other technologies. An additional five others may represent false positives due to their low (2%–17%) variant frequency in SureSelect and SeqCap (HaloPlex had no coverage on four of these variants), but high frequency in AmpliSeq, possibly indicating AmpliSeq over‐representing these variants. Furthermore, five other variants may represent AmpliSeq false positives given that they had 0% variant frequency from all other technologies. Lastly, 16 others may represent AmpliSeq true positives, but had no quality coverage from the other three technologies. Finally, we Sanger sequenced four SNVs that were nominated solely by AmpliSeq. Three SNVs showed the reference allele, whereas one showed a true variant. The latter SNV was missed by the other technologies due to poor or no coverage.

Overall, we found that the amplicon‐based technologies were most discordant largely due to coverage issues. To assess the impact of sequencing depth, we randomly downsampled FASTQ files (1 million, 10 million, 25 million, and 50 million paired‐end fragments) for each sample, called variants with VarScan2, GATK, and MuTect, and compared concordance between technologies (Supp. Fig. S11). As before, when VarScan2 was used to call SNVs, the amplicon‐based technology, HaloPlex, nominated many SNVs not called by either SureSelect or SeqCap. Also, when VarScan2 and MuTect were used to call SNVs, HaloPlex missed many SNVs called by SureSelect and SeqCap. In a second approach, we identified the set of SNVs detected by all technologies at 50 million reads and then randomly downsampled to 25 million, 10 million, and 1 million reads to assess the percentage of SNVs that were still called at lower sequencing depths (Supp. Fig. S12). It is interesting to note that HaloPlex generally detected the highest percentage of common SNVs over these downsampling depths.

### Concordance of SNV Callers with Agilent's SureCall Software

We used Agilent's recommended variant calling software, SureCall‐2.1.1.13, to call SNVs on our HaloPlex libraries (Supp. Table S20). Using these SNVs, we observed concordance with our HaloPlex SNV calls on VarScan2, GATK, and MuTect (Supp. Fig. S13). We noted that SureCall's concordance with GATK, 83.64%, was far higher than its concordance with VarScan2 and MuTect, 74.13% and 74.25%. Despite this difference, we found all three variant callers to have high concordance with SureCall.

### Detection of Previously Validated SNVs

We assessed each technology's detection of previously confirmed variants from the public domain. Therefore, for BT‐20, MCF‐7, and HCC‐2218 cancer cell lines, we took all SNVs in the Cancer Cell Line Encyclopedia (CCLE) [Barretina et al., 2012] that fell in commonly targeted regions (39 SNVs for BT‐20, 14 for MCF‐7, and 38 for HCC‐2218) and calculated the percentage of such SNVs that were called by each technology by each variant caller (Supp. Table S21) (HCC‐2218BL is not in the CCLE). While hybridization capture‐based technologies captured more variants in the CCLE than did amplicon‐based methods on average (ranged between, depending on variant caller, 78.02%–83.52% and 73.63%–82.42% for hybridization capture‐based and amplicon‐based technologies, respectively), due to low sample size, a two‐tailed Mann–Whitney *U*‐test failed to reveal a significant difference (*P* = 0.27). Note that SureCall detected the same percentage as VarScan2 in HaloPlex of SNVs in the CCLE. Also, GATK, on average, picked up more CCLE variants than did VarScan2 or MuTect. Considering that the SNVs in the CCLE have been previously confirmed, we investigated reasons different technologies/variant callers missed these mutations (Supp. Table S22). In almost all cases, we found that the variant frequency of the missed SNVs was less than our minimum 20% cutoff.

### Concordance of Technologies in Indel Calling

Having evaluated each technology's ability to call SNVs, we next investigated each technology's ability to call indels. We used VarScan2, GATK, and Pindel [Ye et al., 2009] to call indels in SureSelect, SeqCap, HaloPlex libraries, whereas the Ion Torrent Suite was used to call indels in AmpliSeq libraries. We filtered for exonic and frameshift or nonframeshift indels in commonly targeted regions and then compared concordance between technologies (Supp. Fig. S14 and Supp. Table S23). As with SNVs, both amplicon‐based technologies showed the most discordance: they each nominated more indels that were unsupported by at least one other technology and missed more indels that were called by the other three technologies.

Furthermore, we evaluated discordant indels in AmpliSeq data, because homopolymers are known to be problematic on Ion Torrent platform for indel calls [Bragg et al., 2013]. For example, AmpliSeq called 596 unique indels not called by any other technology and the majority (529, 88.76%) were 1 bp (Supp. Fig. S14A with Varscan2). Manual inspection of some of these indels showed that they were not necessarily in homopolymer regions. Similarly, with GATK and Pindel, 546 of 576 (94.79%) and 562 of 595 (94.45%) of unique indels, respectively, called by AmpliSeq were 1 bp. In contrast, AmpliSeq missed 398 indels called by all other technologies, with VarScan2, and 206 (51.76%) were 1 bp, and manual inspection of some showed that most of them were in homopolymer regions. Similarly, with GATK and Pindel, 377 of 565 (66.73%) and 211 of 320 (65.94%) of unique indels, respectively, missed by AmpliSeq were 1 bp.

### Evaluation of Copy Number Variation Calling Against SNP Array

**Figure 6:**
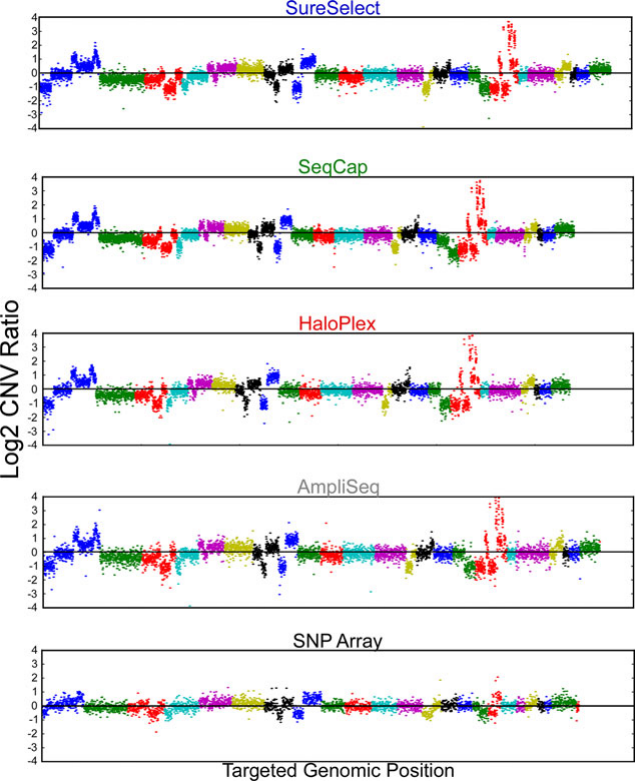
Global copy‐number variation. Global copy number (log_2_ ratio) for the HCC‐2218 cell line against its matched normal; HCC‐2218BL for all technologies and a conventional SNP array. For each technology, we plotted points with matching sequencing and SNP array copy‐number ratios, whereas for the SNP array, we plotted all log_2_ copy‐number ratios. Each color represents a different chromosome.

Next, we assessed detection of copy number variation utilizing read depth. We determined CNVs in the HCC‐2218 cancer cell line with respect to its matched normal cell line, HCC‐2218BL, and compared this with SNP array reference data for the same sample [Redon et al., 2006] (Fig. 6; Supp. Table S24). We contrasted the log_2_–CNV ratios of each technology against the SNP array for bases in each technology's targeted region (Supp. Fig. S15). We examined specifically high‐level copy gains and losses [Frampton et al., 2013; Pritchard et al., 2014] and therefore removed bases whose sequencing log_2_–CNV ratio was between −0.5 and 0.5, because we considered such bases copy neutral or beyond the accuracy for exome sequencing. Correlation of log_2_–CNV ratios between whole‐exome sequencing and SNP array was between 0.80 and 0.84 for all technologies (regression analysis test *P* < 10^−300^ for all technologies).

## Discussion

NGS technologies have enabled whole‐genome sequencing to identify potential phenotype‐altering mutations. However, despite the decreased cost of sequencing, whole‐genome sequencing remains costly [Metzker, 2010; Sulonen et al., 2011]. Further, for cancer research applications, higher depth of coverage is typically desired due to tumor samples having admixtures with normal tissue and tumor heterogeneity. Therefore, whole‐exome sequencing, which still captures most possible phenotype‐altering mutations within exons, may be more cost‐effective to achieve the necessary depth to call such variants [Gnirke et al., 2009]. For investigators seeking to utilize whole‐exome sequencing for research or clinical applications, there are two major choices to consider, the method for whole‐exome capture and the sequencing platform. First, whole‐exome capture methods can be divided into hybridization‐based and newer amplicon‐based capture methods that differ in their ability to uniformly capture and sequence targeted regions and identify mutations. Second, there are two predominant choices for sequencing platforms, Illumina and Ion Torrent technologies [Loman et al., 2012]. A unique limitation for Ion Torrent is the incidence of errors in sequencing homopolymer regions [Bragg et al., 2013], which requires the use of their free proprietary software for alignment and mutation calling that recalibrates for these errors. Meanwhile, Illumina technology has well‐known base substitution errors as well [Meacham et al., 2011; Nakamura et al., 2011]. Several previous reports have compared the earliest versions of SureSelect, SeqCap, Illumina's TruSeq, and Illumina's Nextera kits [Asan et al., 2011; Clark et al., 2011; Parla et al., 2011; Sulonen et al., 2011; Chilamakuri et al., 2014]. However, prior comparison studies have not assessed the new amplicon‐based strategies, compared with Ion Torrent sequencing platforms, or performed in‐depth analysis of SNVs and CNVs. Because of the experimental and sequencing platform differences, we assessed benefits and drawbacks of amplicon‐based (HaloPlex and AmpliSeq) and hybridization capture‐based (SureSelect and SeqCap) methods and sequenced them on either the Ion Proton^TM^ System (AmpliSeq) or the Illumina HiSeq2000 (SureSelect, SeqCap, HaloPlex) (Table 2).

**Table 2:** Performance Comparison of Four Whole‐Exome Capture Methods

	SureSelect (Agilent)	SeqCap (Nimblegen)	HaloPlex (Agilent)	AmpliSeq (Life Technologies)
Sample preparation time	2.5 days	4.5 days	1.5 days	6 hr
Recommended DNA input (μg)	2	1	0.2	0.05
Alignment (manufacturer specified)	30%–70%^a^	70%–80%^a^	30%–70%^a^	>90%^b^
Alignment (this study)	84.97%^a^	79.71%^a^	97.09%^a^	93.68%^a^
Library complexity	60.10%	67.21%	3.24%	20.92%
Base calls on positive strand	49.90%	50.11%	50.05%	45.52%
Uniformity	High	High	Low	Low
SNV	√	√	√	√
CNV	√	√	√	√

a Alignment denotes the percentage of mapped reads that aligned or should align to targeted regions.

b Alignment denotes the percentage of bases that cover the target region.

We found that the hybridization capture‐based methods performed better with respect to sequencing complexity and uniformity (Fig. 3). Hybridization capture‐based methods were less likely than amplicon‐based methods to nominate false‐positive SNVs that were unsupported by at least one other technology (Fig. 5). Also, the hybridization capture‐based methods were less likely to exclude an SNV (false negative) that was nominated by all three other technologies (Fig. 5). While both amplicon‐based methods were vulnerable to false‐positive and false‐negative SNVs, algorithms could be modified to filter or correct these known problems by adjusting parameters, such as minimum variant frequency or minimum read coverage, considering that insufficient read coverage and variant frequency were the most common reasons for false negatives. Sanger sequencing of eight SNVs called solely by HaloPlex or AmpliSeq confirmed 75% of these SNVs to be false positives. The other two variants were true positives, in each case missed due to lack of quality coverage or low‐variant fraction. Furthermore, we observed that some variant calling algorithms might not be ideal for all types of whole‐exome capture, and may need optimization. As an example, motifs causing Illumina sequencing errors [Meacham et al., 2011; Nakamura et al., 2011] contributed to false‐positive SNVs in HaloPlex (it should be noted that Illumina sequencing errors affected HaloPlex more when VarScan2, rather than GATK, was used to call variants and that these sequencing errors also affect SureSelect and SeqCap, but not to the extent they do HaloPlex). Also, homopolymers causing Ion sequencing errors [Bragg et al., 2013] contributed to false‐positive and false‐negative SNVs in AmpliSeq.

Next, we considered pragmatic considerations related to sample preparation and cost for laboratories applying whole‐exome capture and sequencing. Laboratories with limited experience in NGS sample preparation or access to specialized sonication equipment, might prefer amplicon‐based approaches for their simplified workflow. Additionally, if time is sensitive, the amplicon‐based methods may be desirable, given their shortened preparation time compared with other methods [Simon and Roychowdhury, 2013]. Some projects may be utilizing clinical specimens with small quantities of DNA for input, and amplicon‐based methods permit processing of low‐input DNA samples (Table 2). Finally, cost of sequencing is generally comparable, but may also be affected by desired depth of coverage and target region size and on‐target rate (Table 1; Fig. 4). Nonetheless, while amplicon‐based methods have some advantages for sample preparation, there may be some drawbacks with respect to sequencing complexity and coverage uniformity (Table 2).

In summary, we evaluated hybridization and amplicon‐based exome capture methods, considering uniformity of sequencing, variant calling, and sample preparation. We have shown the various advantages and disadvantages of each approach due to differences in sample preparation and probe design. In light of our results, our laboratory prefers capture‐based approaches, mostly due to their sample complexity and coverage uniformity. These data may be useful to other laboratories for selecting their preferred whole‐exome strategy and for benchmarking novel approaches.

*Disclosure statement*: S.R.’s immediate family member owns stock in Johnson and Johnson. E.L. is a former employee of Roche NimbleGen.

## Accession Numbers

All sequencing data have been submitted to the Database of Genotypes and Phenotypes (dbGaP) and is available under the accession number phs000938.v1.p1.

## Supporting information

Disclaimer: Supplementary materials have been peer‐reviewed but not copyedited.

Figure S1
Supporting Table S1 | General sample features
Supporting Table S7 | VarScan2 single‐sample SNVs from SureSelect in commonly targeted bases
Supporting Table S8 | VarScan2 single‐sample SNVs from SeqCap in commonly targeted bases
Supporting Table S9 | VarScan2 single‐sample SNVs from HaloPlex in commonly targeted bases
Supporting Table S10 | GATK single‐sample SNVs from SureSelect in commonly targeted bases
Supporting Table S10 | GATK single‐sample SNVs from SeqCap in commonly targeted bases
Supporting Table S12 | GATK single‐sample SNVs from HaloPlex in commonly targeted bases
Supporting Table S13 | MuTect single‐sample SNVs from SureSelect in commonly targeted bases
Supporting Table S14 | MuTect single‐sample SNVs from SeqCap in commonly targeted bases
Supporting Table S15 | MuTect single‐sample SNVs from HaloPlex in commonly targeted bases
Supporting Table S16 | Ion Torrent Suite single‐sample SNVs from AmpliSeq in commonly targeted bases
Supporting Table S20 | SureCall single‐sample SNVs from HaloPlex in commonly targeted bases
Table S21 | Number/Percent of SNVs in the CCLE in commonly targeted regions called by each technology
Supporting Table S22 | Reasons Different Technologies Missed CCLE Mutations in Commonly Targeted Regions
Supporting Table S23 | Indels in Commonly Targeted Regions
Supporting Table S24 | Comparison of CNV calling HCC‐2218 to HCC‐2218BL on VarScan2 and SNP array

## References

[humu22825-bib-0001] Albert TJ , Molla MN , Muzny DM , Nazareth L , Wheeler D , Song X , Richmond TA , Middle CM , Rodesch MJ , Packard CJ , Weinstock GM , Gibbs RA . 2007 Direct selection of human genomic loci by microarray hybridization. Nat Methods 4:903–905. 17934467 10.1038/nmeth1111

[humu22825-bib-0002] Asan, Xu Y , Jiang H , Tyler‐Smith C , Xue Y , Jiang T , Wang J , Wu M , Liu X , Tian G , Yang H , Zhang X . 2011 Comprehensive comparison of three commercial human whole‐exome capture platforms. Genome Biol 12:R95. 21955857 10.1186/gb-2011-12-9-r95 PMC3308058

[humu22825-bib-0003] Barretina J , Caponigro G , Stransky N , Venkatesan K , Margolin AA , Kim S , Wilson CJ , Lehar J , Kryukov GV , Sonkin D , Reddy A , Liu M , et al. 2012 The Cancer Cell Line Encyclopedia enables predictive modelling of anticancer drug sensitivity. Nature 483:603–607. 22460905 10.1038/nature11003 PMC3320027

[humu22825-bib-0004] Bragg LM , Stone G , Butler MK , Hugenholtz P , Tyson GW . 2013 Shining a light on dark sequencing: characterising errors in Ion Torrent PGM data. PLoS Comput Biol 9:e1003031. 23592973 10.1371/journal.pcbi.1003031 PMC3623719

[humu22825-bib-0005] Chilamakuri CS , Lorenz S , Madoui MA , Vodak D , Sun J , Hovig E , Myklebost O , Meza‐Zepeda LA . 2014 Performance comparison of four exome capture systems for deep sequencing. BMC Genomics 15:449. 24912484 10.1186/1471-2164-15-449 PMC4092227

[humu22825-bib-0006] Cibulskis K , Lawrence MS , Carter SL , Sivachenko A , Jaffe D , Sougnez C , Gabriel S , Meyerson M , Lander ES , Getz G . 2013 Sensitive detection of somatic point mutations in impure and heterogeneous cancer samples. Nat Biotechnol 31:213–219. 23396013 10.1038/nbt.2514 PMC3833702

[humu22825-bib-0007] Clamp M , Fry B , Kamal M , Xie X , Cuff J , Lin MF , Kellis M , Lindblad‐Toh K , Lander ES . 2007 Distinguishing protein‐coding and noncoding genes in the human genome. Proc Natl Acad Sci USA 104:19428–19433. 18040051 10.1073/pnas.0709013104 PMC2148306

[humu22825-bib-0008] Clark MJ , Chen R , Lam HY , Karczewski KJ , Euskirchen G , Butte AJ , Snyder M . 2011 Performance comparison of exome DNA sequencing technologies. Nat Biotechnol 29:908–914. 21947028 10.1038/nbt.1975 PMC4127531

[humu22825-bib-0009] Daley T , Smith AD . 2013 Predicting the molecular complexity of sequencing libraries. Nat Methods 10:325–327. 23435259 10.1038/nmeth.2375 PMC3612374

[humu22825-bib-0010] Frampton GM , Fichtenholtz A , Otto GA , Wang K , Downing SR , He J , Schnall‐Levin M , White J , Sanford EM , An P , Sun J , Juhn F , et al. 2013 Development and validation of a clinical cancer genomic profiling test based on massively parallel DNA sequencing. Nat Biotechnol 31:1023–1031. 24142049 10.1038/nbt.2696 PMC5710001

[humu22825-bib-0011] Gnirke A , Melnikov A , Maguire J , Rogov P , LeProust EM , Brockman W , Fennell T , Giannoukos G , Fisher S , Russ C , Gabriel S , Jaffe DB , et al. 2009 Solution hybrid selection with ultra‐long oligonucleotides for massively parallel targeted sequencing. Nat Biotechnol 27:182–189. 19182786 10.1038/nbt.1523 PMC2663421

[humu22825-bib-0012] Hodges E , Xuan Z , Balija V , Kramer M , Molla MN , Smith SW , Middle CM , Rodesch MJ , Albert TJ , Hannon GJ , McCombie WR . 2007 Genome‐wide in situ exon capture for selective resequencing. Nat Genet 39:1522–1527. 17982454 10.1038/ng.2007.42

[humu22825-bib-0013] Koboldt DC , Zhang Q , Larson DE , Shen D , McLellan MD , Lin L , Miller CA , Mardis ER , Ding L , Wilson RK . 2012 VarScan 2: somatic mutation and copy number alteration discovery in cancer by exome sequencing. Genome Res 22:568–576. 22300766 10.1101/gr.129684.111 PMC3290792

[humu22825-bib-0014] Li H , Durbin R . 2010 Fast and accurate long‐read alignment with Burrows‐Wheeler transform. Bioinformatics 26:589–595. 20080505 10.1093/bioinformatics/btp698 PMC2828108

[humu22825-bib-0015] Li H , Handsaker B , Wysoker A , Fennell T , Ruan J , Homer N , Marth G , Abecasis G , Durbin R , Genome Project Data Processing S. 2009a The Sequence Alignment/Map format and SAMtools. Bioinformatics 25:2078–2079. 19505943 10.1093/bioinformatics/btp352 PMC2723002

[humu22825-bib-0016] Li R , Li Y , Fang X , Yang H , Wang J , Kristiansen K , Wang J . 2009b SNP detection for massively parallel whole‐genome resequencing. Genome Res 19:1124–1132. 19420381 10.1101/gr.088013.108 PMC2694485

[humu22825-bib-0017] Liu X , Han S , Wang Z , Gelernter J , Yang BZ . 2013 Variant callers for next‐generation sequencing data: a comparison study. PLoS One 8:e75619. 24086590 10.1371/journal.pone.0075619 PMC3785481

[humu22825-bib-0018] Loman NJ , Misra RV , Dallman TJ , Constantinidou C , Gharbia SE , Wain J , Pallen MJ . 2012 Performance comparison of benchtop high‐throughput sequencing platforms. Nat Biotechnol 30:434–439. 22522955 10.1038/nbt.2198

[humu22825-bib-0019] McKenna A , Hanna M , Banks E , Sivachenko A , Cibulskis K , Kernytsky A , Garimella K , Altshuler D , Gabriel S , Daly M , DePristo MA . 2010 The Genome Analysis Toolkit: a MapReduce framework for analyzing next‐generation DNA sequencing data. Genome Res 20:1297–1303. 20644199 10.1101/gr.107524.110 PMC2928508

[humu22825-bib-0020] Meacham F , Boffelli D , Dhahbi J , Martin DI , Singer M , Pachter L . 2011 Identification and correction of systematic error in high‐throughput sequence data. BMC Bioinformatics 12:451. 22099972 10.1186/1471-2105-12-451 PMC3295828

[humu22825-bib-0021] Metzker ML . 2010 Sequencing technologies—the next generation. Nat Rev Genet 11:31–46. 19997069 10.1038/nrg2626

[humu22825-bib-0022] Nakamura K , Oshima T , Morimoto T , Ikeda S , Yoshikawa H , Shiwa Y , Ishikawa S , Linak MC , Hirai A , Takahashi H , Altaf‐Ul‐Amin M , Ogasawara N , et al. 2011 Sequence‐specific error profile of Illumina sequencers. Nucleic Acids Res 39:e90. 21576222 10.1093/nar/gkr344 PMC3141275

[humu22825-bib-0023] Parla JS , Iossifov I , Grabill I , Spector MS , Kramer M , McCombie WR . 2011 A comparative analysis of exome capture. Genome Biol 12:R97. 21958622 10.1186/gb-2011-12-9-r97 PMC3308060

[humu22825-bib-0024] Pritchard CC , Salipante SJ , Koehler K , Smith C , Scroggins S , Wood B , Wu D , Lee MK , Dintzis S , Adey A , Liu Y , Eaton KD , et al. 2014 Validation and implementation of targeted capture and sequencing for the detection of actionable mutation, copy number variation, and gene rearrangement in clinical cancer specimens. J Mol Diagn 16:56–67. 24189654 10.1016/j.jmoldx.2013.08.004 PMC3873496

[humu22825-bib-0025] Quinlan AR , Hall IM . 2010 BEDTools: a flexible suite of utilities for comparing genomic features. Bioinformatics 26:841–842. 20110278 10.1093/bioinformatics/btq033 PMC2832824

[humu22825-bib-0026] Redon R , Ishikawa S , Fitch KR , Feuk L , Perry GH , Andrews TD , Fiegler H , Shapero MH , Carson AR , Chen W , Cho EK , Dallaire S , et al. 2006 Global variation in copy number in the human genome. Nature 444:444–454. 17122850 10.1038/nature05329 PMC2669898

[humu22825-bib-0027] Roberts ND , Kortschak RD , Parker WT , Schreiber AW , Branford S , Scott HS , Glonek G , Adelson DL . 2013 A comparative analysis of algorithms for somatic SNV detection in cancer. Bioinformatics 29:2223–2230. 23842810 10.1093/bioinformatics/btt375 PMC3753564

[humu22825-bib-0028] Robinson JT , Thorvaldsdottir H , Winckler W , Guttman M , Lander ES , Getz G , Mesirov JP . 2011 Integrative genomics viewer. Nat Biotechnol 29:24–26. 21221095 10.1038/nbt.1754 PMC3346182

[humu22825-bib-0029] Sherry ST , Ward MH , Kholodov M , Baker J , Phan L , Smigielski EM , Sirotkin K . 2001 dbSNP: the NCBI database of genetic variation. Nucleic Acids Res 29:308–311. 11125122 10.1093/nar/29.1.308 PMC29783

[humu22825-bib-0030] Simon R , Roychowdhury S . 2013 Implementing personalized cancer genomics in clinical trials. Nat Rev Drug Discov 12:358–369. 23629504 10.1038/nrd3979

[humu22825-bib-0031] Sulonen AM , Ellonen P , Almusa H , Lepisto M , Eldfors S , Hannula S , Miettinen T , Tyynismaa H , Salo P , Heckman C , Joensuu H , Raivio T , et al. 2011 Comparison of solution‐based exome capture methods for next generation sequencing. Genome Biol 12:R94. 21955854 10.1186/gb-2011-12-9-r94 PMC3308057

[humu22825-bib-0032] Wang K , Li M , Hakonarson H . 2010 ANNOVAR: functional annotation of genetic variants from high‐throughput sequencing data. Nucleic Acids Res 38:e164. 20601685 10.1093/nar/gkq603 PMC2938201

[humu22825-bib-0033] Wang Q , Jia P , Li F , Chen H , Ji H , Hucks D , Dahlman KB , Pao W , Zhao Z . 2013 Detecting somatic point mutations in cancer genome sequencing data: a comparison of mutation callers. Genome Med 5:91. 24112718 10.1186/gm495 PMC3971343

[humu22825-bib-0034] Ye K , Schulz MH , Long Q , Apweiler R , Ning Z . 2009 Pindel: a pattern growth approach to detect break points of large deletions and medium sized insertions from paired‐end short reads. Bioinformatics 25:2865–2871. 19561018 10.1093/bioinformatics/btp394 PMC2781750

