# Supplementary material for: Evaluation of Hybridization Capture Versus Amplicon‐Based Methods for Whole‐Exome Sequencing
Source: Hum Mutat. 2015 Jul 15;36(9):903–14. doi: 10.1002/humu.22825 (PMC4832303; doi:10.1002/humu.22825)
Supplement: Supplementary file 1 — Figure S1 Supporting Table S1 | General sample features Supporting Table S7 | VarScan2 single‐sample SNVs from SureSelect in commonly targeted bases Supporting Table S8 | VarScan2 single‐sample SNVs from SeqCap in commonly targeted bases Supporting Table S9 | VarScan2 single‐sample SNVs from HaloPlex in commonly targeted bases Supporting Table S10 | GATK single‐sample SNVs from SureSelect in commonly targeted bases Supporting Table S10 | GATK single‐sample SNVs from SeqCap in commonly targeted bases Supporting Table S12 | GATK single‐sample SNVs from HaloPlex in commonly targeted bases Supporting Table S13 | MuTect single‐sample SNVs from SureSelect in commonly targeted bases Supporting Table S14 | MuTect single‐sample SNVs from SeqCap in commonly targeted bases Supporting Table S15 | MuTect single‐sample SNVs from HaloPlex in commonly targeted bases Supporting Table S16 | Ion Torrent Suite single‐sample SNVs from AmpliSeq in commonly targeted bases Supporting Table S20 | SureCall single‐sample SNVs from HaloPlex in commonly targeted bases Table S21 | Number/Percent of SNVs in the CCLE in commonly targeted regions called by each technology Supporting Table S22 | Reasons Different Technologies Missed CCLE Mutations in Commonly Targeted Regions Supporting Table S23 | Indels in Commonly Targeted Regions Supporting Table S24 | Comparison of CNV calling HCC‐2218 to HCC‐2218BL on VarScan2 and SNP array [file HUMU-36-903-s001.zip › humu22825-sup-0001-SuppMat.docx]

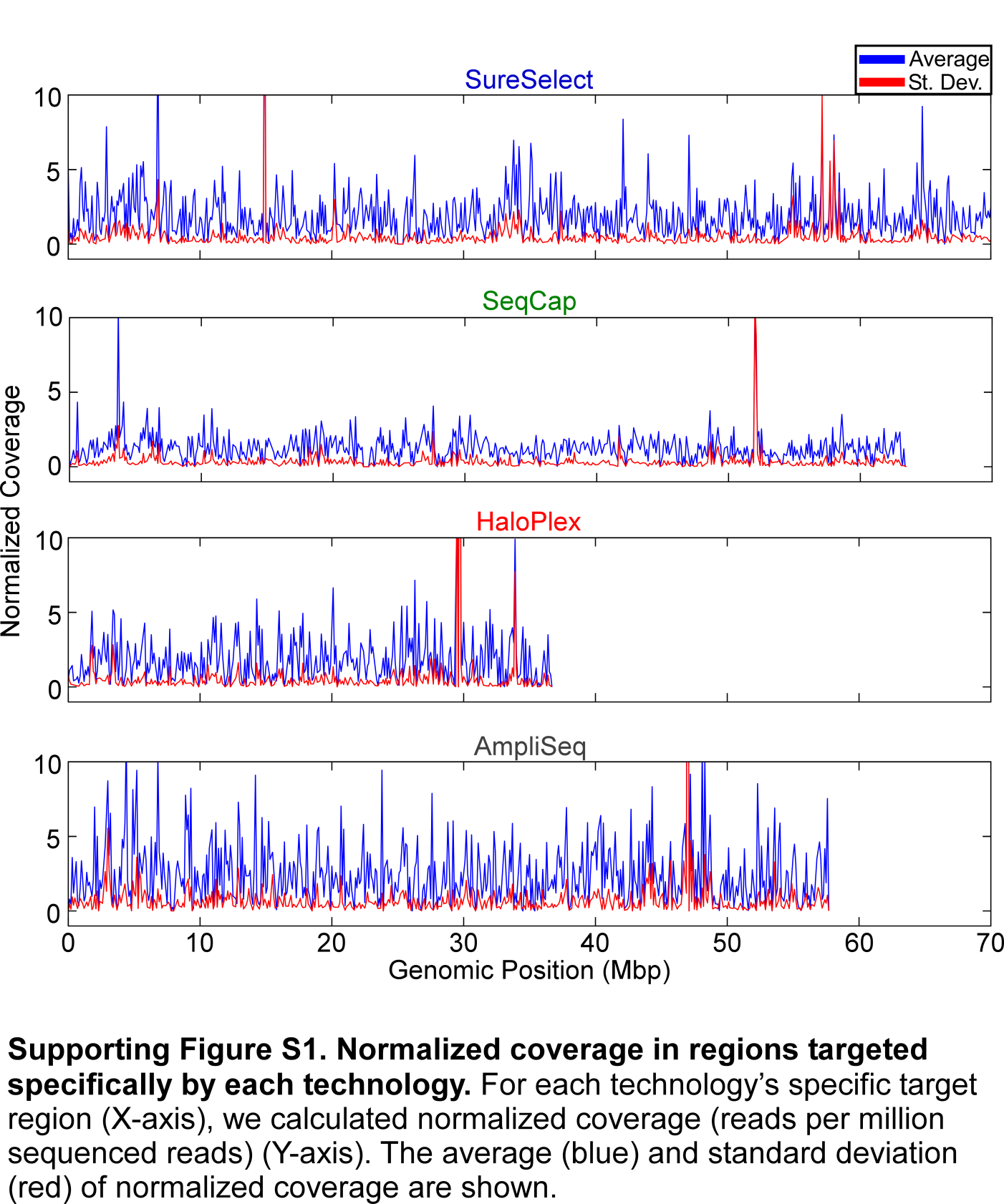


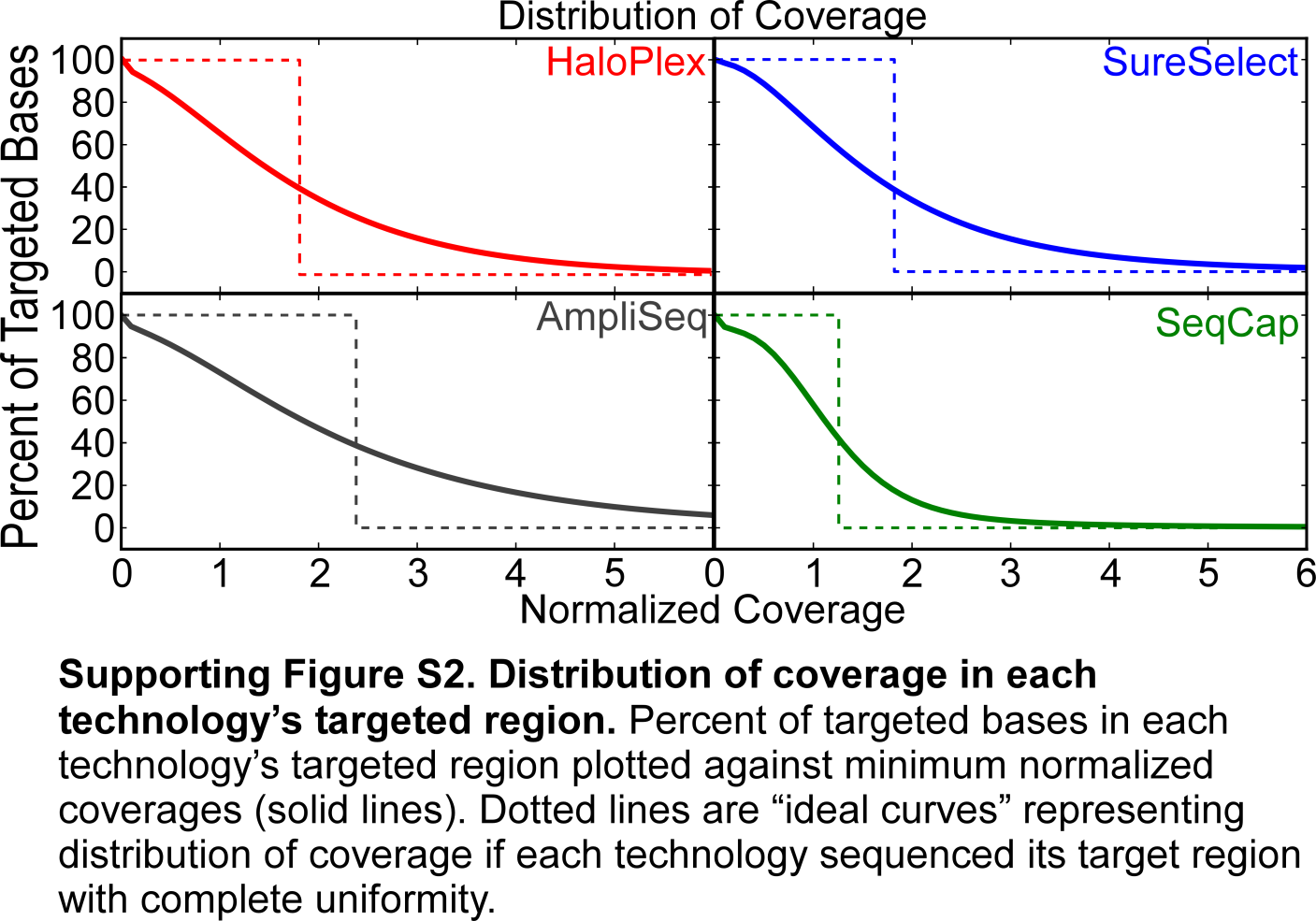


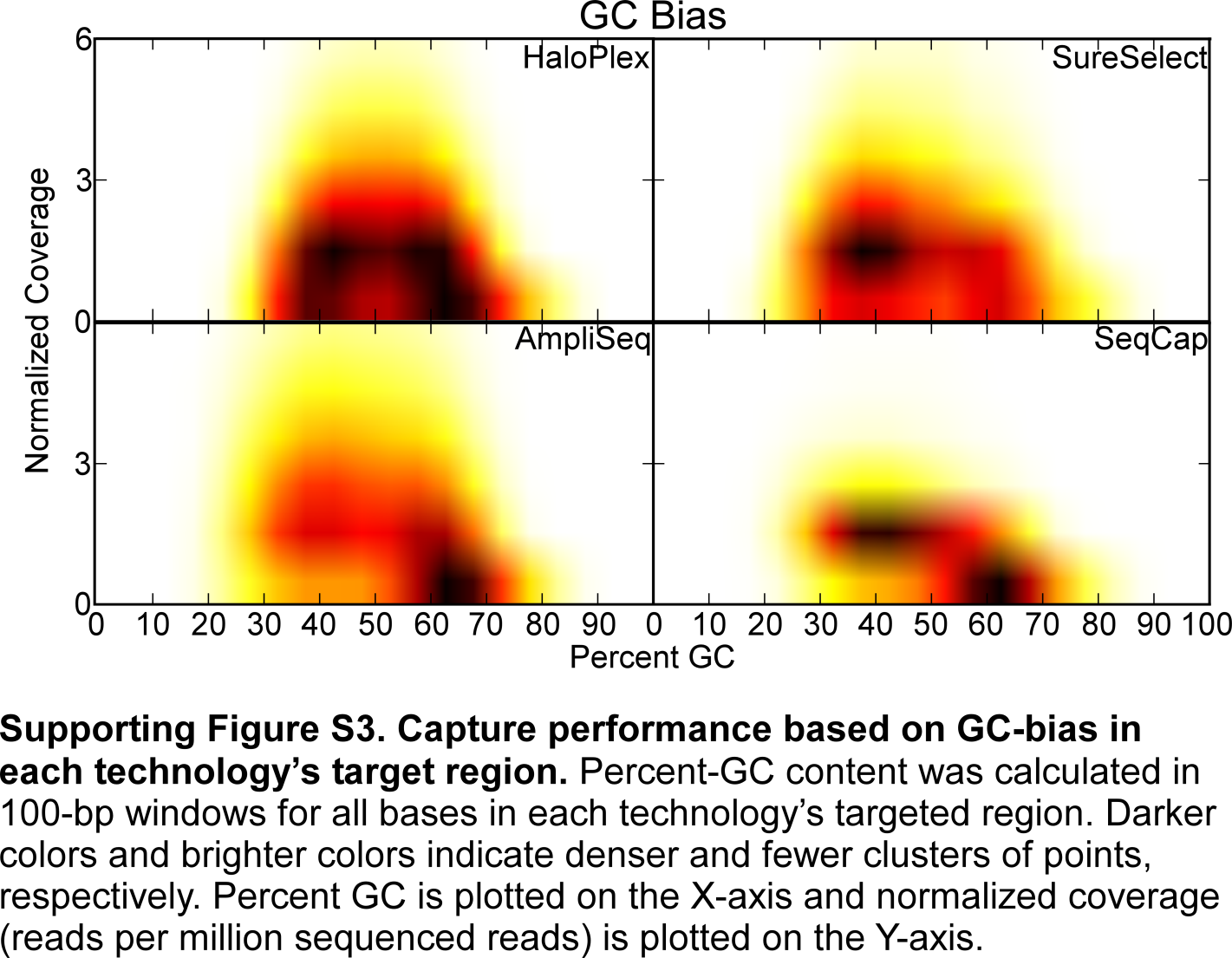


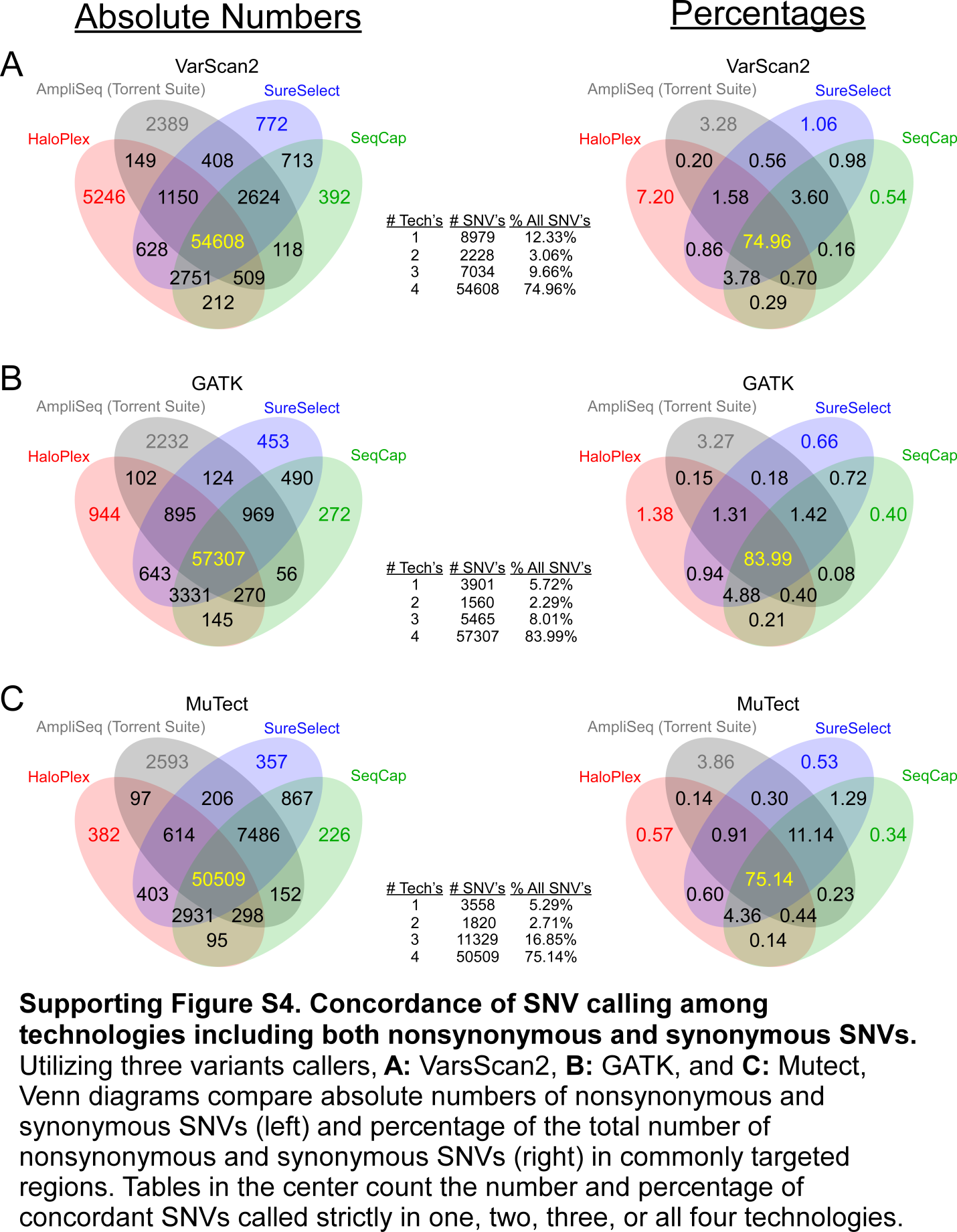


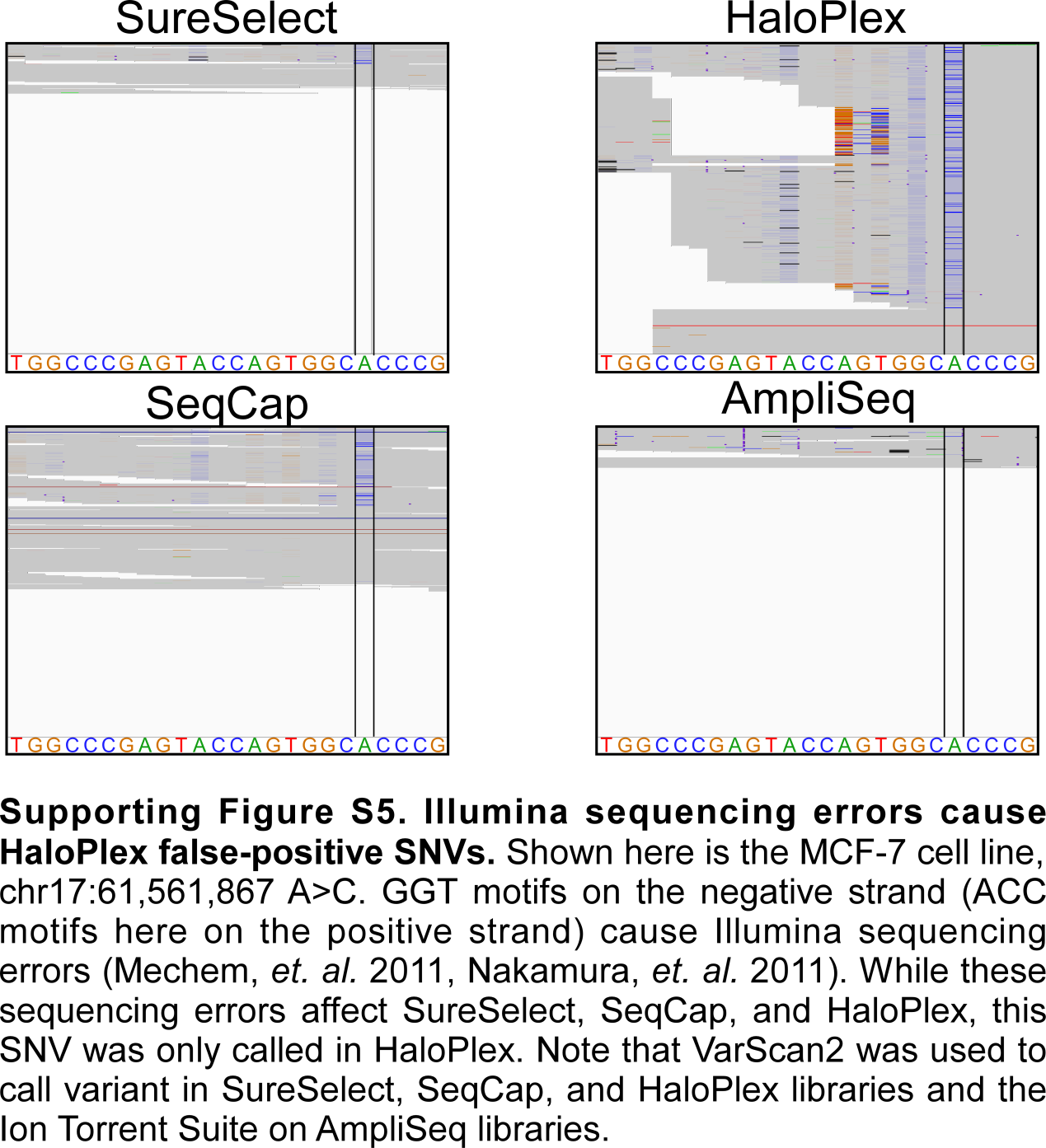


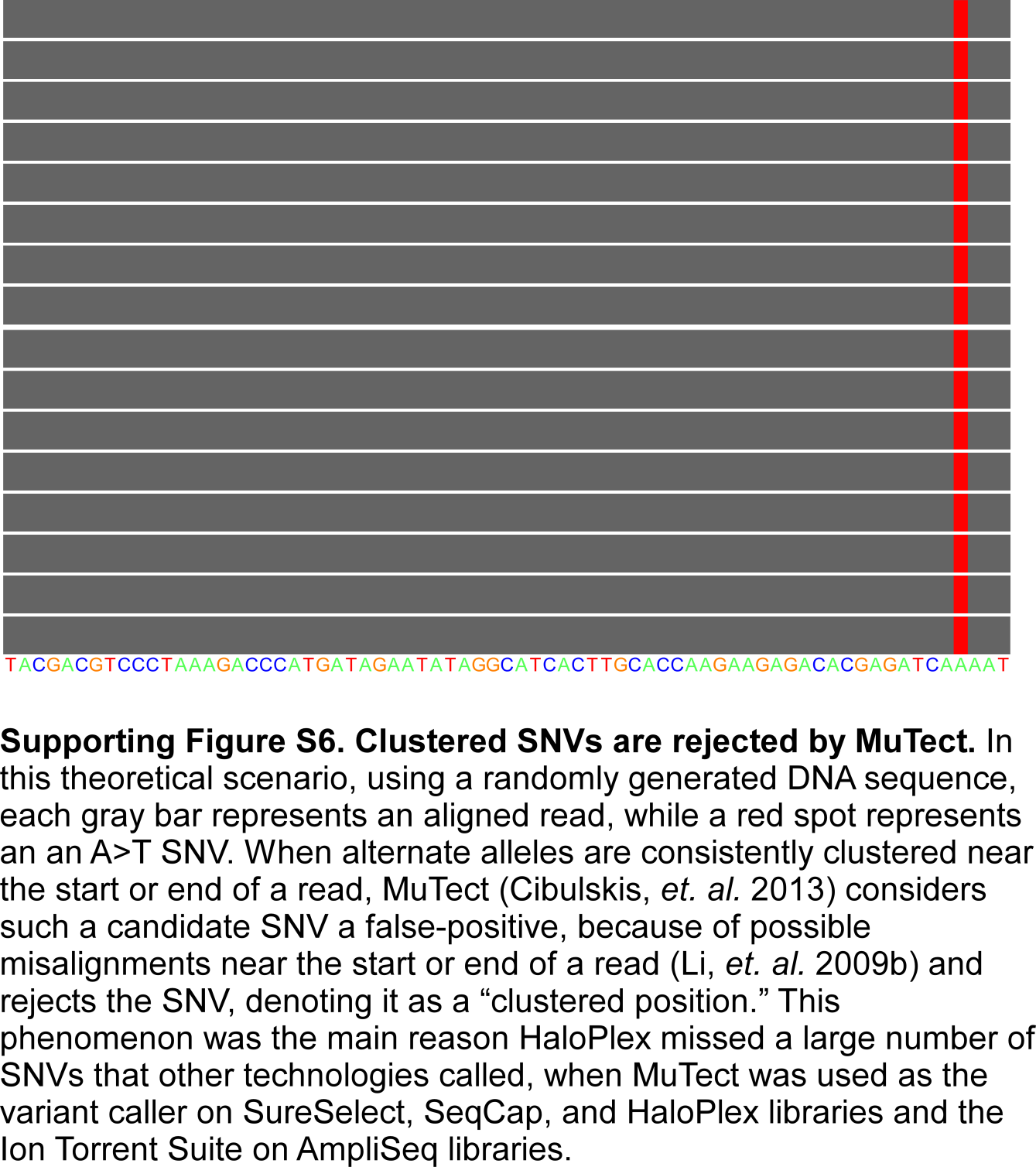


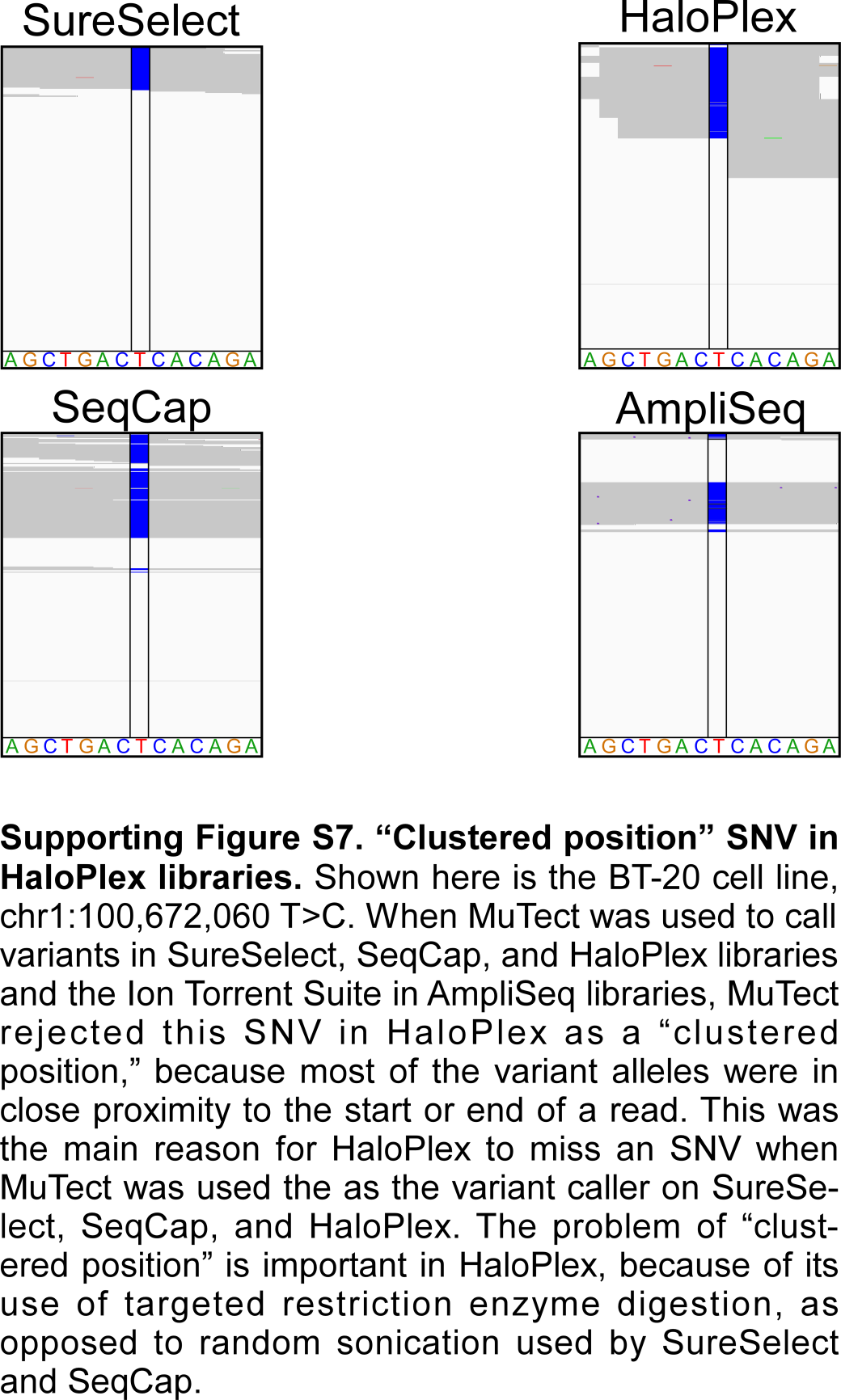


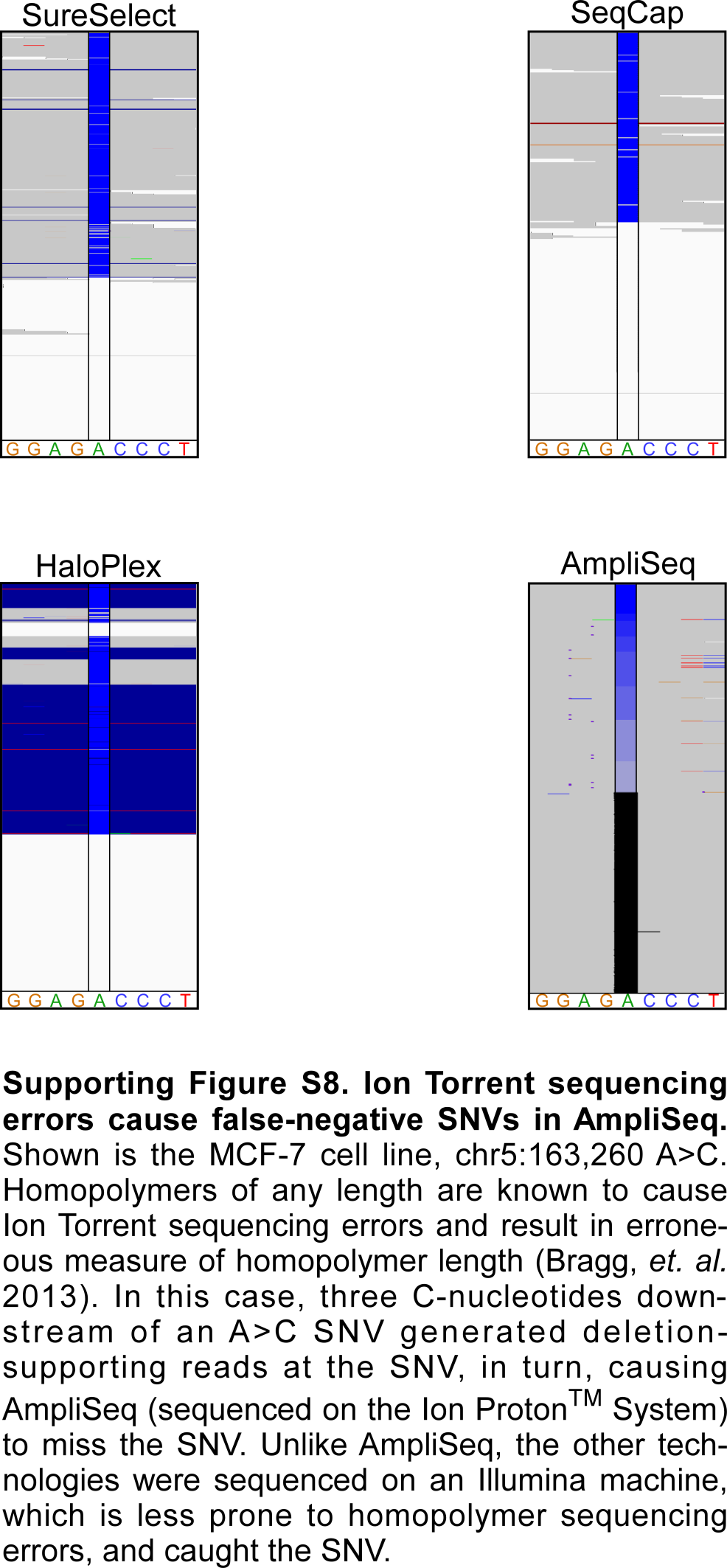

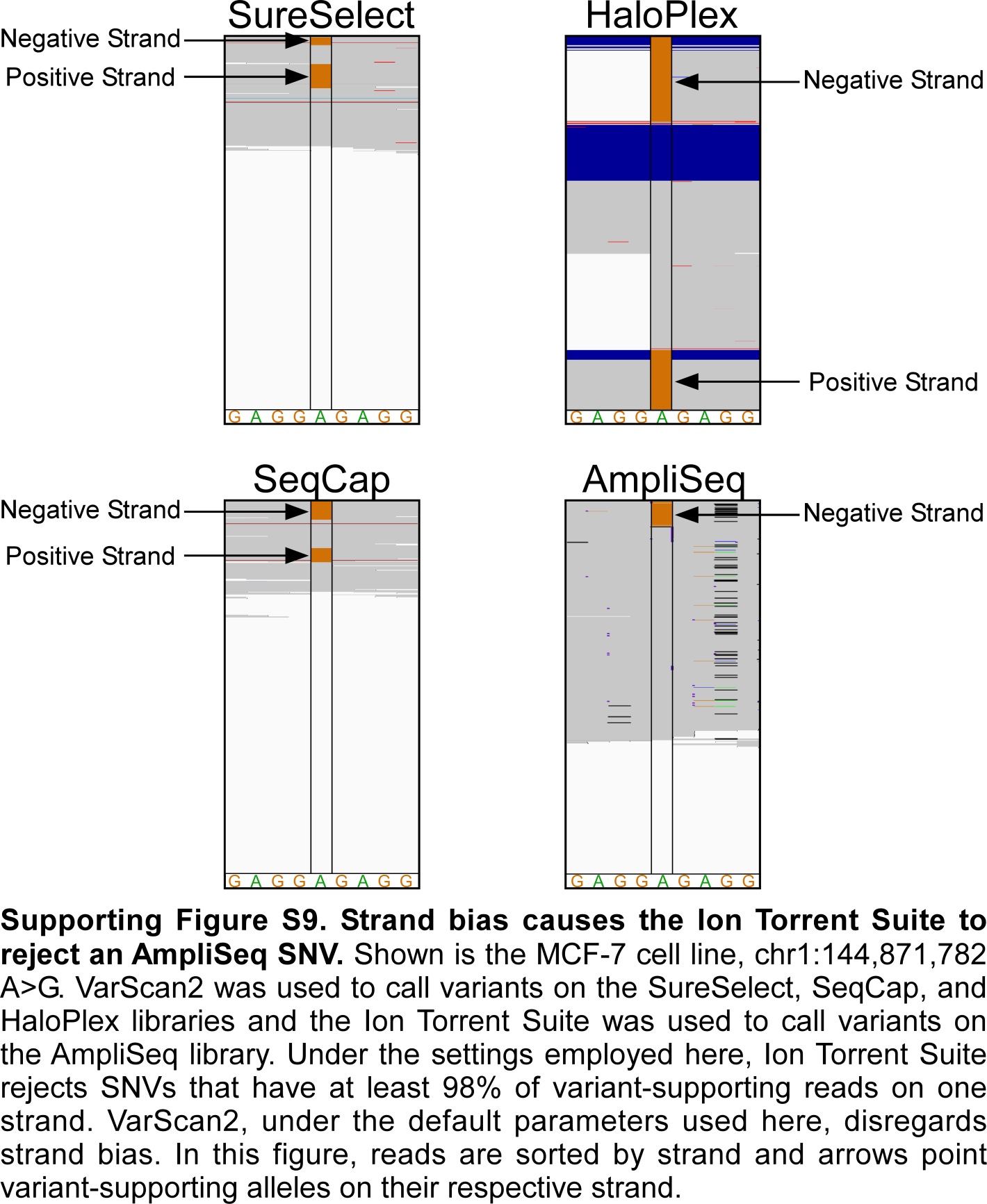


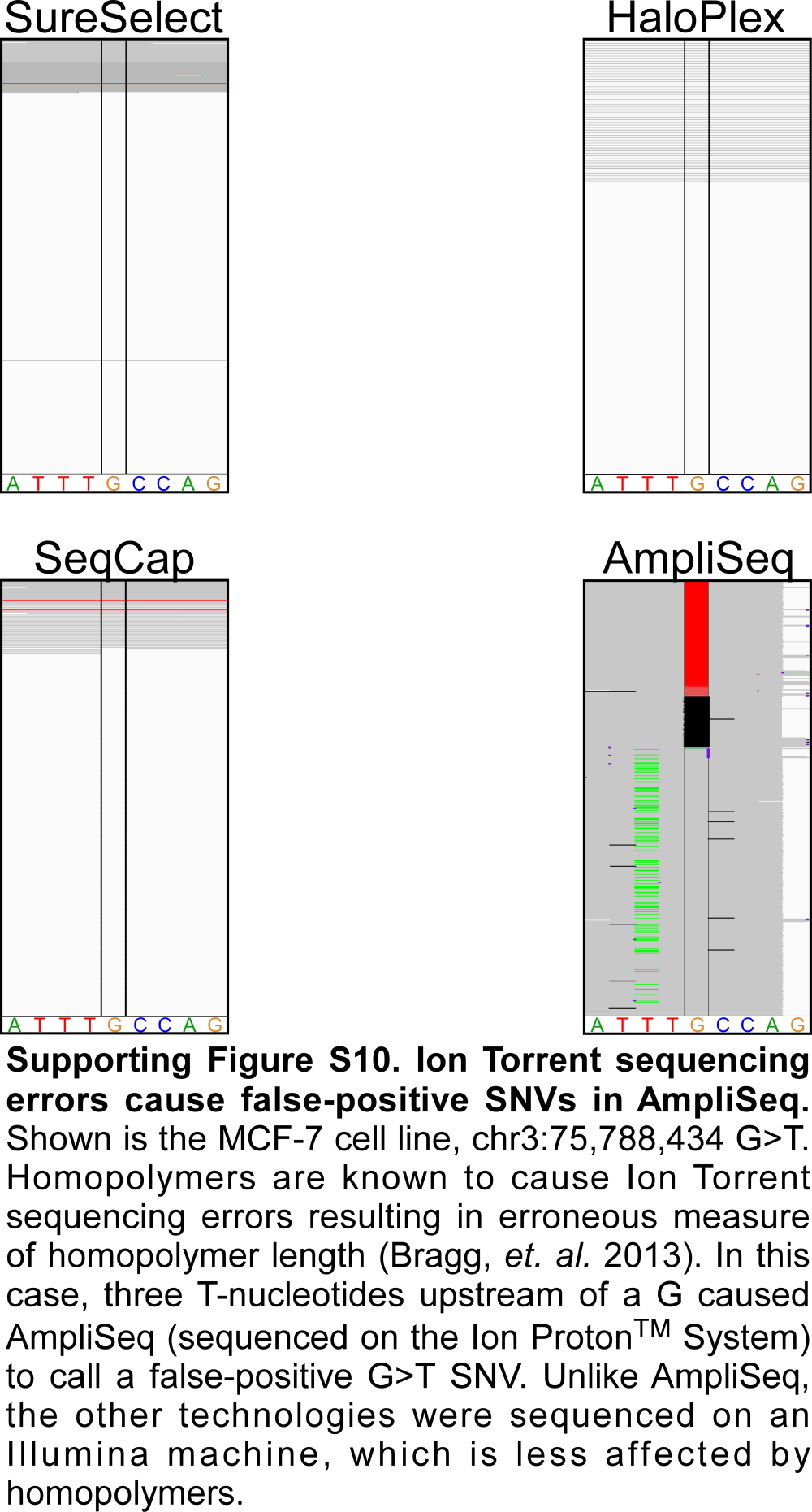


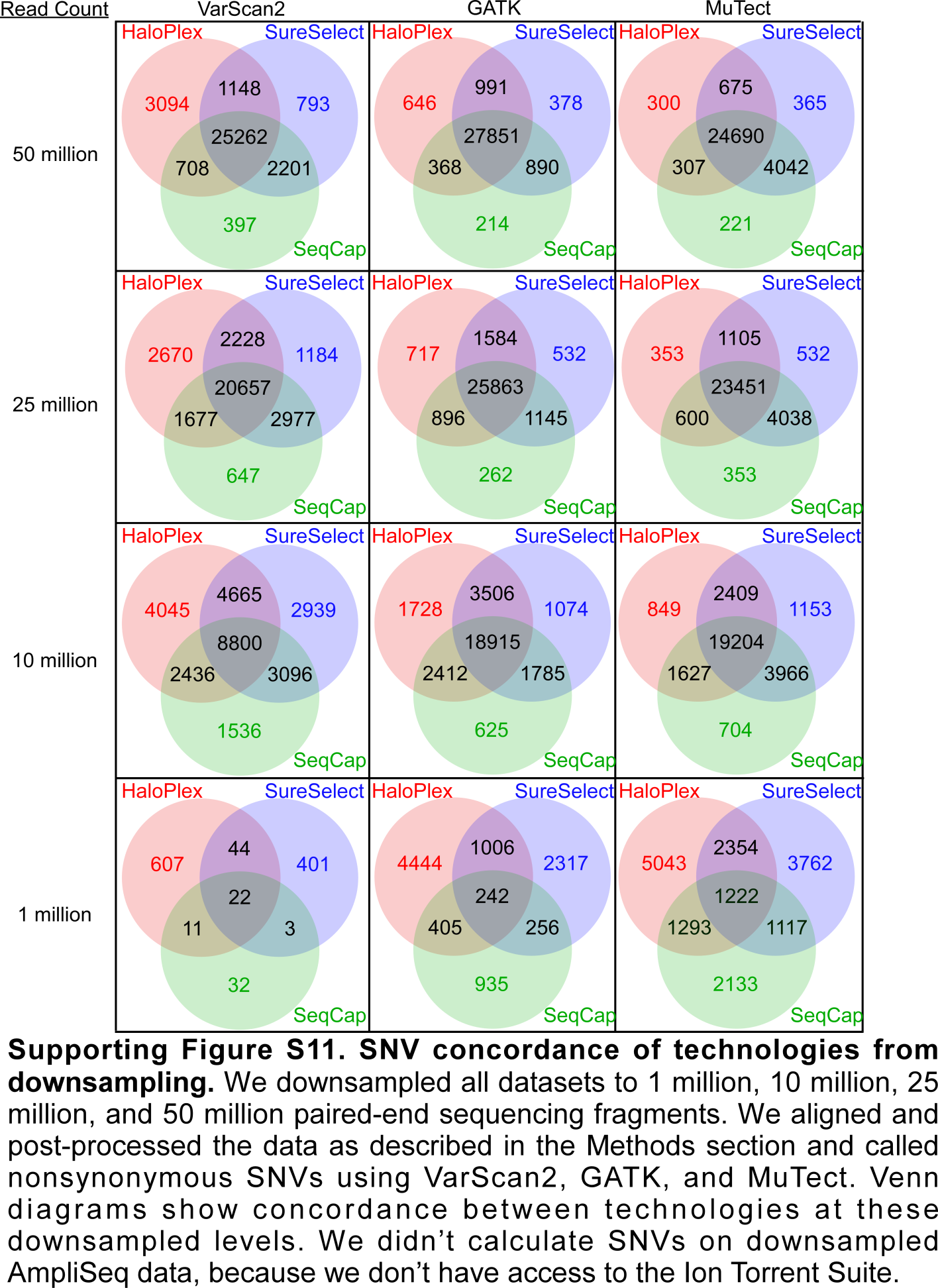


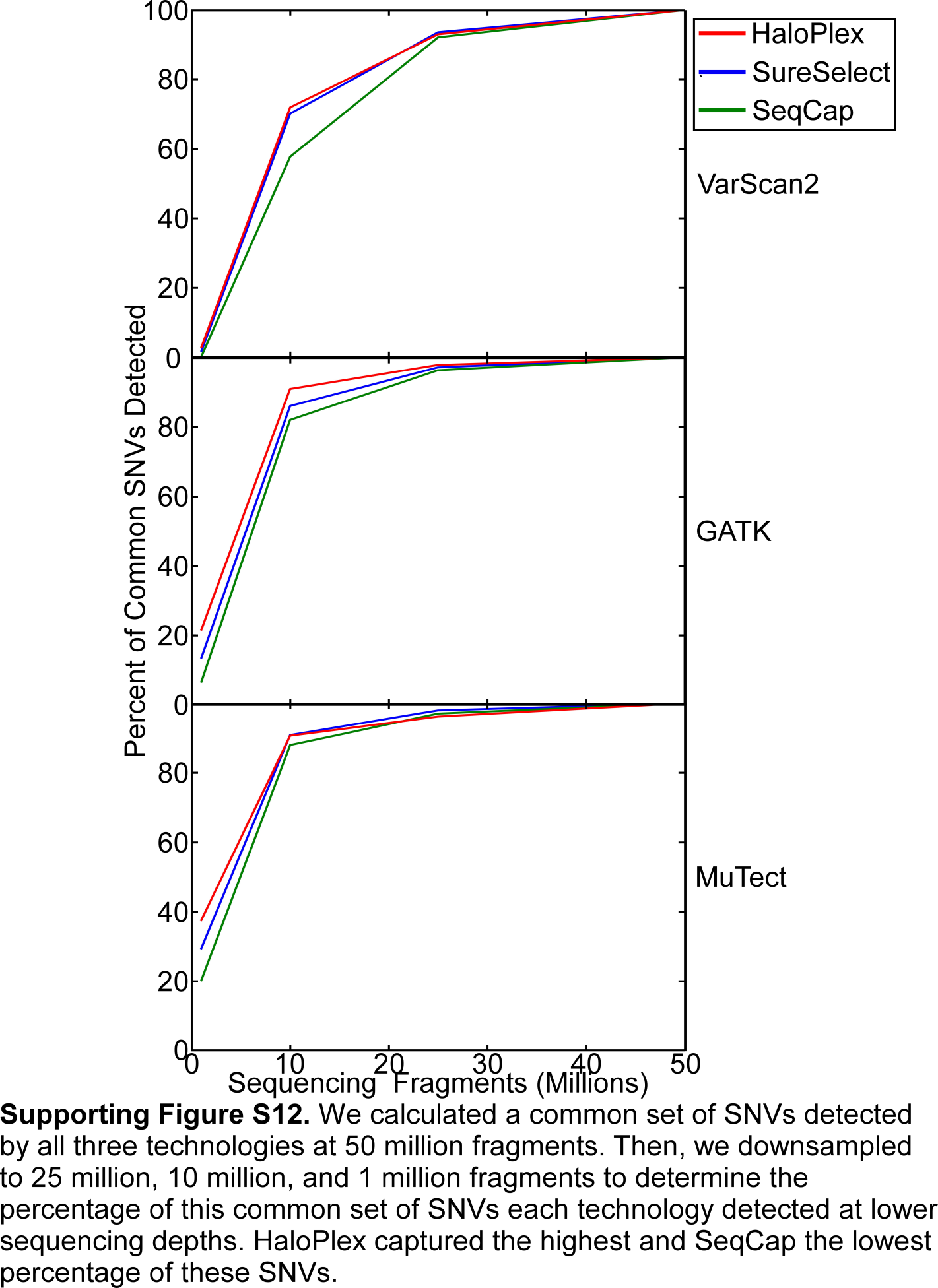


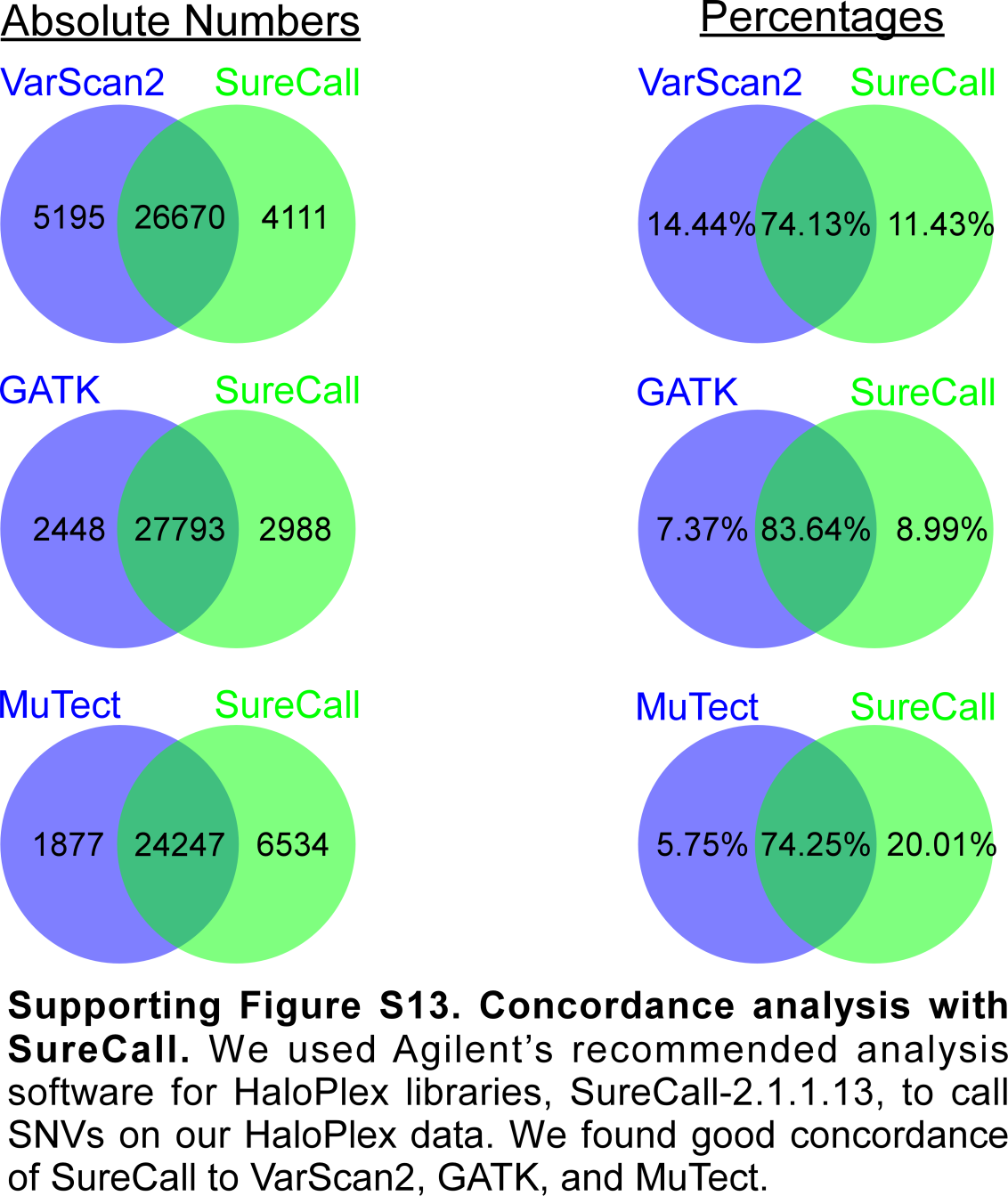


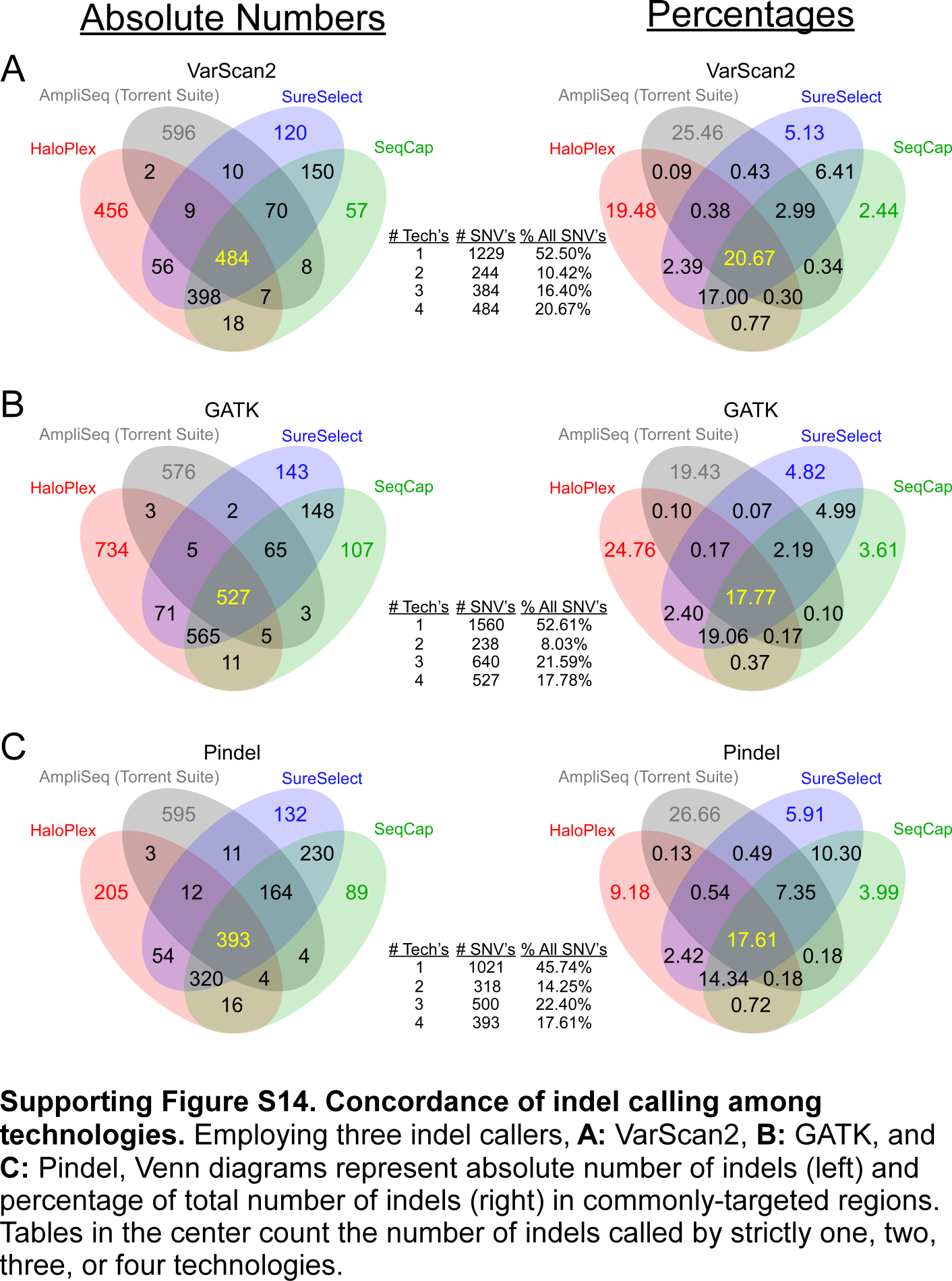


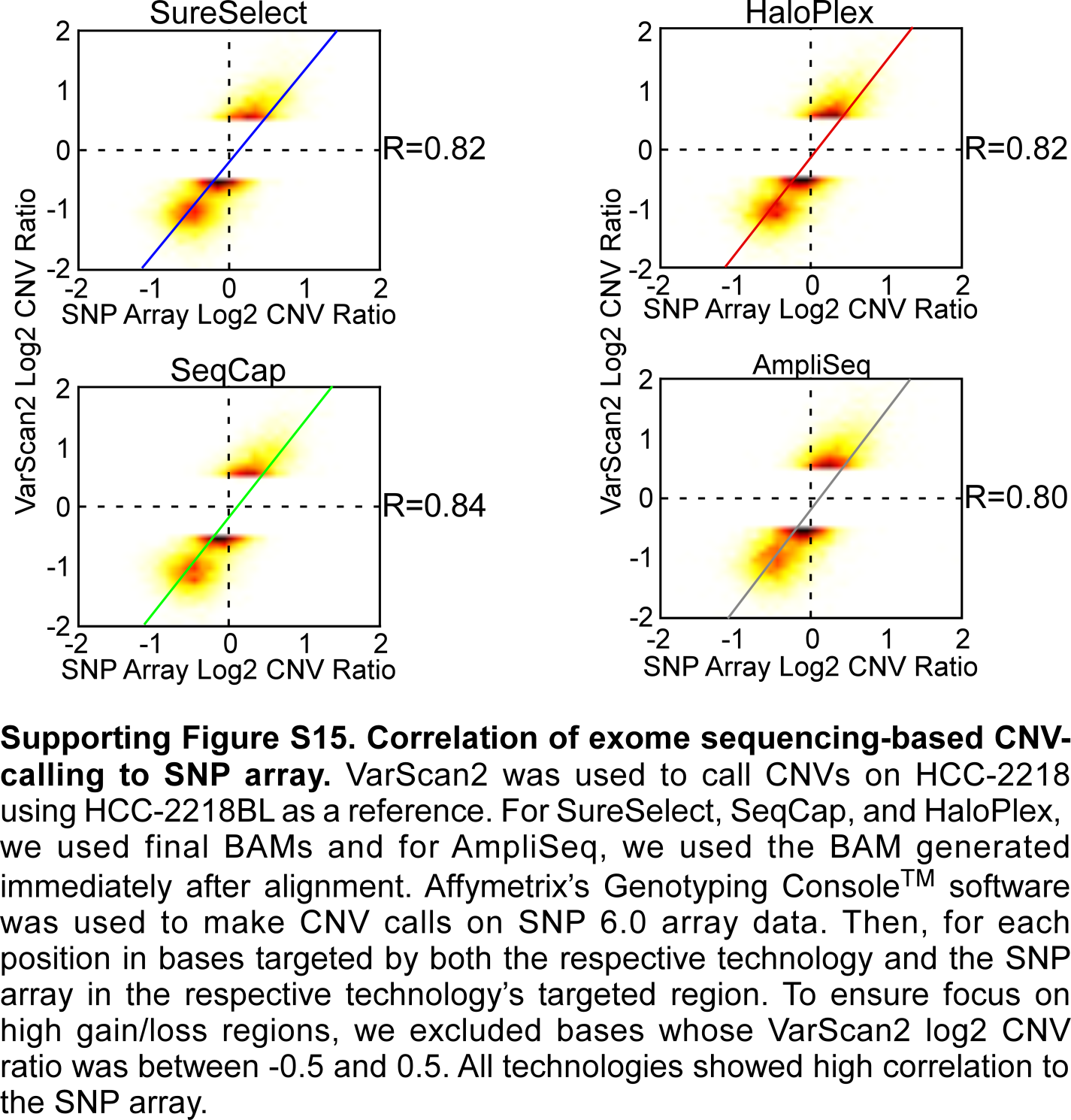


| **Supp. Table S2. AmpliSeq settings** | |
| --- | --- |
|  |  |
| snp_min_allele_freq | 0.1 |
| snp_strand_bias | 0.98 |
| hotspot_min_coverage | 6 |
| hotspot_min_cov_each_strand | 3 |
| hotspot_min_allele_freq | 0.1 |
| snp_min_variant_score | 15 |
| hotspot_strand_bias | 0.95 |
| hp_max_length | 8 |
| filter_insertion_predictions | 0.2 |
| indel_min_variant_score | 20 |
| indel_min_coverage | 10 |
| heavy_tailed | 3 |
| outlier_probability | 0.01 |
| data_quality_stringency | 5 |
| snp_min_cov_each_strand | 0 |
| hotspot_min_variant_score | 10 |
| indel_strand_bias | 0.9 |
| tvc_parameters_version | germline_low_stringency_proton-3.6.66827 |
| downsample_to_coverage | 400 |
| filter_unusual_predictions | 0.25 |
| indel_min_allele_freq | 0.15 |
| do_snp_realignment | 1 |
| prediction_precision | 1 |
| indel_min_cov_each_strand | 5 |
| filter_deletion_predictions | 0.2 |
| suppress_recalibration | 0 |
| snp_min_coverage | 5 |

**Supp. Tables S1**, **S7-S16, and S20-S24** are available as separate Excel files under the Supporting Information for this article.

| **Supp. Table S3. P-values for all Mann-Whitney U tests** | | | | |
| --- | --- | --- | --- | --- |
|  |  |  |  |  |
|  | **Complexity** | | | |
|  | **SureSelect** | **SeqCap** | **HaloPlex** | **AmpliSeq** |
| **SureSelect** | - | 0.03 | 0.03 | 0.03 |
| **SeqCap** | - | - | 0.03 | 0.03 |
| **HaloPlex** | - | - | - | 0.03 |
| **AmpliSeq** | - | - | - | - |
|  |  |  |  |  |
|  | **Avg. Norm. Cov (common region)** | | | |
|  | **SureSelect** | **SeqCap** | **HaloPlex** | **AmpliSeq** |
| **SureSelect** | - | <1e-323 | <1e-323 | <1e-323 |
| **SeqCap** | - | - | <1e-323 | <1e-323 |
| **HaloPlex** | - | - | - | <1e-323 |
| **AmpliSeq** | - | - | - | - |
|  |  |  |  |  |
|  | **St. Dev. Norm. Cov tech (specific region)** | | | |
|  | **SureSelect** | **SeqCap** | **HaloPlex** | **AmpliSeq** |
| **SureSelect** | - | <1e-323 | <1e-323 | <1e-323 |
| **SeqCap** | - | - | <1e-323 | <1e-323 |
| **HaloPlex** | - | - | - | <1e-323 |
| **AmpliSeq** | - | - | - | - |
|  |  |  |  |  |
|  | **#CCLE Variants Detected** | | | |
|  | **SureSelect** | **SeqCap** | **HaloPlex** | **AmpliSeq** |
| **SureSelect** | - | 0.56 | 0.45 | 0.93 |
| **SeqCap** | - | - | 0.17 | 1 |
| **HaloPlex** | - | - | - | 0.42 |
| **AmpliSeq** | - | - | - | - |
|  |  |  |  |  |
|  | **Mapped to hg19** | | | |
|  | **SureSelect** | **SeqCap** | **HaloPlex** | **AmpliSeq** |
| **SureSelect** | - | 0.03 | 0.03 | 0.02 |
| **SeqCap** | - | - | 0.03 | 0.02 |
| **HaloPlex** | - | - | - | 0.02 |
| **AmpliSeq** | - | - | - | - |
|  |  |  |  |  |
|  | **Avg. Norm. Cov (tech specific region)** | | | |
|  | **SureSelect** | **SeqCap** | **HaloPlex** | **AmpliSeq** |
| **SureSelect** | - | <1e-323 | <1e-323 | <1e-323 |
| **SeqCap** | - | - | <1e-323 | <1e-323 |
| **HaloPlex** | - | - | - | <1e-323 |
| **AmpliSeq** | - | - | - | - |
|  |  |  |  |  |
|  | **Dev. from Ideal Curve (common region)** | | | |
|  | **SureSelect** | **SeqCap** | **HaloPlex** | **AmpliSeq** |
| **SureSelect** | - | <1e-323 | <1e-323 | <1e-323 |
| **SeqCap** | - | - | <1e-323 | <1e-323 |
| **HaloPlex** | - | - | - | <1e-323 |
| **AmpliSeq** | - | - | - | - |
|  |  |  |  |  |
|  | **hg19 to Targets** | | | |
|  | **SureSelect** | **SeqCap** | **HaloPlex** | **AmpliSeq** |
| **SureSelect** | - | 0.03 | 0.03 | 0.03 |
| **SeqCap** | - | - | 0.03 | 0.03 |
| **HaloPlex** | - | - | - | 0.03 |
| **AmpliSeq** | - | - | - | - |
|  |  |  |  |  |
|  | **St. Dev. Norm. Cov (common region)** | | | |
|  | **SureSelect** | **SeqCap** | **HaloPlex** | **AmpliSeq** |
| **SureSelect** | - | <1e-323 | <1e-323 | <1e-323 |
| **SeqCap** | - | - | <1e-323 | <1e-323 |
| **HaloPlex** | - | - | - | <1e-323 |
| **AmpliSeq** | - | - | - | - |
|  |  |  |  |  |
|  | **Dev. from Ideal Curve (tech-spec region)** | | | |
|  | **SureSelect** | **SeqCap** | **HaloPlex** | **AmpliSeq** |
| **SureSelect** | - | <1e-323 | <1e-323 | <1e-323 |
| **SeqCap** | - | - | <1e-323 | <1e-323 |
| **HaloPlex** | - | - | - | <1e-323 |
| **AmpliSeq** | - | - | - | - |

| **Supp. Table S4. Alignment rates** | | | |
| --- | --- | --- | --- |
|  |  |  |  |
|  | **SureSelect** | | |
| **Sample** | **Pct. Fastq to Genome** | **Pct. Fastq to Target** | **Pct. Genome to Target** |
| **BT-20** | 91.27 | 77.4 | 84.81 |
| **MCF-7** | 91.99 | 78.05 | 84.84 |
| **H2218BL** | 91.32 | 78.2 | 85.63 |
| **H2218** | 91.86 | 78.17 | 85.1 |
|  |  |  |  |
| **Median** | 91.59 | 78.11 | 84.97 |
| **Med. Abs. Dev.** | 0.295 | 0.075 | 0.145 |
|  |  |  |  |
|  | **SeqCap** | | |
| **Sample** | **Pct. Fastq to Genome** | **Pct. Fastq to Target** | **Pct. Genome to Target** |
| **BT-20** | 95.12 | 77.3 | 81.27 |
| **MCF-7** | 94.99 | 63.2 | 66.53 |
| **H2218BL** | 94.14 | 75.56 | 80.26 |
| **H2218** | 93.77 | 74.22 | 79.15 |
|  |  |  |  |
| **Median** | 94.565 | 74.89 | 79.705 |
| **Med. Abs. Dev.** | 0.49 | 1.54 | 1.06 |
|  |  |  |  |
|  | **HaloPlex** | | |
| **Sample** | **Pct. Fastq to Genome** | **Pct. Fastq to Target** | **Pct. Genome to Target** |
| **BT-20** | 96.3 | 92.15 | 95.69 |
| **MCF-7** | 97.23 | 94.26 | 96.95 |
| **H2218BL** | 97.87 | 94.78 | 96.85 |
| **H2218** | 97.72 | 93.91 | 96.09 |
|  |  |  |  |
| **Median** | 97.475 | 94.085 | 96.47 |
| **Med. Abs. Dev.** | 0.32 | 0.435 | 0.43 |
|  |  |  |  |
|  | **AmpliSeq** | | |
| **Sample** | **Pct. Fastq to Genome** | **Pct. Fastq to Target** | **Pct. Genome to Target** |
| **BT-20** | 99.97 | 93.65 | 93.68 |
| **MCF-7** | 99.97 | 93.65 | 93.68 |
| **H2218BL** | 99.97 | 94.03 | 94.06 |
| **H2218** | 99.97 | 93.53 | 93.56 |
|  |  |  |  |
| **Median** | 99.97 | 93.65 | 93.68 |
| **Med. Abs. Dev.** | 0 | 0.06 | 0.06 |

| **Supp. Table S5. Average and st. dev. of normalized coverage in technology-specified regions and common regions (all units are reads per million sequenced reads)** | | | | |
| --- | --- | --- | --- | --- |
|  |  |  |  |  |
|  | **Average** | | | |
|  | **Common Regions** | | **Technology-Specified Regions** | |
|  | **Average** | **St. Dev.** | **Average** | **St. Dev.** |
| **SureSelect** | 1.82 | 1.49 | 1.82 | 1.49 |
| **SeqCap** | 1.23 | 0.73 | 1.26 | 0.97 |
| **HaloPlex** | 1.85 | 1.56 | 1.8 | 1.56 |
| **AmpliSeq** | 2.54 | 2.26 | 2.38 | 2.19 |
|  |  |  |  |  |
|  | **St. Dev.** | | | |
|  | **Common Regions** | | **Technology-Specified Regions** | |
|  | **Average** | **St. Dev.** | **Average** | **St. Dev.** |
| **SureSelect** | 0.45 | 0.78 | 0.45 | 0.77 |
| **SeqCap** | 0.32 | 0.42 | 0.33 | 0.5 |
| **HaloPlex** | 0.47 | 0.88 | 0.46 | 0.85 |
| **AmpliSeq** | 0.69 | 1.23 | 0.65 | 1.18 |

| **Supp. Table S6. Deviations from ideal sequencing (all units are reads per million sequencing reads)** | | | | |
| --- | --- | --- | --- | --- |
|  |  |  |  |  |
|  | **Common Region** | | **Tech-Spec Region** | |
|  | **Average** | **St. Dev.** | **Average** | **St. Dev.** |
| **SureSelect** | 1.03 | 1.09 | 1.04 | 1.06 |
| **SeqCap** | 0.52 | 0.51 | 0.61 | 0.76 |
| **HaloPlex** | 1.12 | 1.08 | 1.12 | 1.08 |
| **AmpliSeq** | 1.59 | 1.6 | 1.54 | 1.56 |

| **Supp. Table S17. Classification of Discordant SNVs** |  |  |  |
| --- | --- | --- | --- |
|  |  |  |  |
|  | **VarScan2** | **GATK** | **MuTect** |
| # Discordant SNVs | 10510 | 6521 | 9126 |
| # SNVs called only by one amplicon-based technology | 5221 | 2315 | 1976 |
| # SNVs missed only by one amplicon-based technology | 2687 | 2305 | 5071 |
| # SNVs called only by one hyb. capture-based technology | 667 | 556 | 296 |
| # SNVs missed only by one hyb. capture-based technology | 723 | 460 | 428 |
|  |  |  |  |
| % SNVs called or missed by only one amplicon-based technology | 75.24263 | 70.84803 | 77.21893 |
| % SNVs called or missed by only one hyb.capture -based technology | 13.2255 | 15.58043 | 7.933377 |

| **Supp. Table S18. Variant Frequency of SNVs missed by only by HaloPlex when called under SureSelect and SeqCap (the variant caller is VarScan2)** | | |
| --- | --- | --- |
|  |  |  |
| **Reason HaloPlex Missed SNV** | **Median SureSelect VF** | **Median SeqCap VF** |
| Less than 8 reads with quality 15 at SNV position | 100.00 | 100.00 |
| VF < 20% | 33.74 | 35.14 |
| VF between 20% and 30%, but failed p-value | 39.62 | 43.83 |
| VF between 30% and 40%, but failed p-value | 43.17 | 45.84 |
| VF between 40% and 50%, but failed p-value | 48.89 | 49.57 |
| VF between 50% and 60%, but failed p-value | 50.56 | 44.44 |
| VF between 60% and 70%, but failed p-value | 52.34 | 44.88 |

| All these SNVs were missed by HaloPlex, but called by SureSelect, SeqCap, and AmpliSeq. | |
| --- | --- |
| The caller for SureSelect, SeqCap, and HaloPlex was VarScan2. |  |
| The following SNVs met VarScan2's minimum raw coverage of 8 reads in HaloPlex. | |

| **Supp. Table S19. Potential false-negative and false-postive SNVs in HaloPlex and AmpliSeq that were investigated** | | | | | |
| --- | --- | --- | --- | --- | --- |
|  |  |  |  |  |  |
| **SNVs called by HaloPlex, but missed by other technologies using VarScan2** | | | | | |
| **Chromosome** | **Position** | **Ref** | **Alt** | **Sample** | **Approx. Read Count** |
| chr12 | 122812693 | G | T | H2218BL | 664 |
| chr16 | 85704622 | T | G | MCF-7 | 671 |
| chr15 | 52613630 | A | C | MCF-7 | 711 |
| chr17 | 61561867 | A | C | MCF-7 | 711 |
| chr1 | 116579977 | G | T | MCF-7 | 774 |
| chr17 | 56581559 | T | G | MCF-7 | 774 |
| chr20 | 60895896 | C | G | BT-20 | 795 |
| chr7 | 55270348 | T | C | BT-20 | 795 |
| chr17 | 72745167 | A | G | H2218 | 833 |
| chr17 | 72745164 | T | G | H2218 | 837 |
| chr17 | 72745155 | A | G | H2218 | 852 |
| chr14 | 20404319 | A | C | MCF-7 | 874 |
| chr11 | 1017783 | A | G | BT-20 | 965 |
| chr20 | 47253062 | T | C | MCF-7 | 1071 |
| chr16 | 85704622 | T | G | BT-20 | 1143 |
| chr1 | 116579977 | G | T | BT-20 | 1545 |
| chr11 | 1017595 | A | C | BT-20 | 1007 |
| chr17 | 21319767 | C | G | BT-20 | 698 |
| chr11 | 1018419 | G | A | H2218 | 1053 |
| chr11 | 1018419 | G | A | BT-20 | 1183 |
| chr12 | 49724412 | T | G | H2218 | 663 |
| chr12 | 49724387 | A | G | H2218 | 664 |
| chr12 | 49724403 | A | G | H2218 | 664 |
| chr1 | 152323339 | C | G | H2218 | 665 |
| chr1 | 186277070 | A | G | MCF-7 | 665 |
| chr5 | 878525 | T | A | BT-20 | 666 |
| chr1 | 152382780 | C | A | H2218 | 674 |
| chr16 | 67320223 | G | A | BT-20 | 697 |
| chr8 | 101719201 | A | G | MCF-7 | 700 |
| chr12 | 49724304 | G | A | H2218BL | 702 |
| chr6 | 112508770 | G | T | BT-20 | 720 |
| chr1 | 152323339 | C | G | MCF-7 | 733 |
| chr12 | 49724387 | A | G | H2218BL | 738 |
| chr12 | 49724403 | A | G | H2218BL | 740 |
| chr12 | 49724412 | T | G | H2218BL | 740 |
| chr12 | 49724313 | G | T | H2218BL | 742 |
| chr1 | 152382780 | C | A | MCF-7 | 752 |
| chr1 | 152382770 | T | C | H2218 | 760 |
| chr1 | 186277070 | A | G | H2218 | 789 |
| chr12 | 49724304 | G | A | MCF-7 | 800 |
| chr1 | 152382770 | T | C | MCF-7 | 802 |
| chr12 | 49724387 | A | G | MCF-7 | 810 |
| chr12 | 49724313 | G | T | MCF-7 | 814 |
| chr12 | 49724403 | A | G | MCF-7 | 816 |
| chr12 | 49724412 | T | G | MCF-7 | 816 |
| chr1 | 117944964 | C | A | BT-20 | 824 |
| chr1 | 152382802 | T | G | H2218 | 839 |
| chr1 | 152382802 | T | G | MCF-7 | 924 |
| chr5 | 173036394 | C | T | BT-20 | 1201 |
| chr20 | 56098733 | T | C | MCF-7 | 1799 |
|  |  |  |  |  |  |
| **SNVs missed by AmpliSeq, but called by other technologies using VarScan2** | | | | | |
| **Chromosome** | **Position** | **Ref** | **Alt** | **Sample** | **Approx. Read Count** |
| chr7 | 157931144 | C | T | MCF-7 | 341 |
| chr7 | 131128350 | G | T | MCF-7 | 343 |
| chr20 | 43723627 | T | C | HCC-2218 | 350 |
| chr11 | 48367097 | A | G | BT-20 | 361 |
| chr11 | 1017302 | G | C | HCC-2218BL | 365 |
| chr3 | 89521693 | T | C | HCC-2218BL | 368 |
| chr11 | 48373986 | A | G | BT-20 | 371 |
| chr9 | 37441650 | T | C | BT-20 | 372 |
| chr17 | 56621286 | G | T | HCC-2218 | 375 |
| chr7 | 157931144 | C | T | BT-20 | 380 |
| chr9 | 140147273 | C | T | HCC-2218BL | 385 |
| chr11 | 85436868 | G | C | MCF-7 | 387 |
| chr15 | 90784207 | A | G | HCC-2218 | 387 |
| chr1 | 175129955 | G | T | HCC-2218 | 393 |
| chr15 | 99653800 | T | C | BT-20 | 403 |
| chr20 | 35769647 | G | T | BT-20 | 408 |
| chr9 | 140777306 | C | G | BT-20 | 416 |
| chr15 | 90784207 | A | G | BT-20 | 422 |
| chr3 | 183754294 | G | C | BT-20 | 429 |
| chr15 | 90784207 | A | G | HCC-2218BL | 431 |
| chr1 | 144871782 | A | G | MCF-7 | 435 |
| chr15 | 90771750 | T | G | HCC-2218BL | 437 |
| chr1 | 34071525 | C | T | MCF-7 | 440 |
| chr19 | 49469223 | C | T | BT-20 | 440 |
| chr1 | 12785494 | G | T | HCC-2218BL | 460 |
| chr11 | 62381106 | G | C | HCC-2218BL | 471 |
| chr3 | 183754278 | C | G | BT-20 | 482 |
| chr17 | 21319171 | G | A | HCC-2218 | 487 |
| chr1 | 145109661 | G | A | HCC-2218BL | 492 |
| chr3 | 38793940 | A | G | BT-20 | 508 |
| chr6 | 57512529 | C | A | HCC-2218BL | 596 |
| chr11 | 48367097 | A | G | MCF-7 | 602 |
| chr11 | 56468155 | T | G | HCC-2218 | 615 |
| chr11 | 56467945 | G | T | HCC-2218BL | 623 |
| chr22 | 37462936 | A | G | HCC-2218BL | 625 |
| chr15 | 90784207 | A | G | MCF-7 | 639 |
| chr5 | 163260 | A | C | HCC-2218BL | 674 |
| chr16 | 70972595 | T | C | MCF-7 | 681 |
| chr11 | 48373885 | A | G | HCC-2218 | 715 |
| chr16 | 70972595 | T | C | HCC-2218 | 751 |
| chr11 | 48373986 | A | G | HCC-2218 | 752 |
| chr11 | 48373885 | A | G | MCF-7 | 796 |
| chr17 | 21319171 | G | A | MCF-7 | 807 |
| chr11 | 48373986 | A | G | MCF-7 | 828 |
| chr11 | 48373885 | A | G | HCC-2218BL | 856 |
| chr11 | 48373986 | A | G | HCC-2218BL | 883 |
| chr5 | 163260 | A | C | MCF-7 | 888 |
| chr5 | 163260 | A | C | HCC-2218 | 1055 |
| chr17 | 21319682 | C | T | BT-20 | 1561 |
| chr17 | 21319682 | C | T | HCC-2218BL | 1702 |
|  |  |  |  |  |  |
| **SNVs called by AmpliSeq, but missed by other technologies using VarScan2** | | | | | |
| **Chromosome** | **Position** | **Ref** | **Alt** | **Sample** | **Approx. Read Count** |
| chr6 | 29364815 | C | G | MCF-7 | 545 |
| chr6 | 29364835 | T | G | MCF-7 | 547 |
| chr6 | 29364838 | C | G | MCF-7 | 547 |
| chrX | 55172537 | G | A | HCC-2218BL | 549 |
| chr6 | 29364787 | C | T | MCF-7 | 557 |
| chr6 | 31084163 | A | G | HCC-2218BL | 568 |
| chr6 | 28963248 | T | G | HCC-2218 | 577 |
| chr17 | 73487884 | A | C | MCF-7 | 580 |
| chr6 | 29364787 | C | T | BT-20 | 587 |
| chr14 | 74060514 | C | A | MCF-7 | 589 |
| chr14 | 74060517 | A | G | MCF-7 | 589 |
| chr20 | 39832235 | G | A | MCF-7 | 591 |
| chr6 | 29364951 | G | A | HCC-2218BL | 603 |
| chr17 | 21319007 | G | A | HCC-2218 | 606 |
| chr11 | 48366971 | G | C | HCC-2218 | 621 |
| chr3 | 105438956 | C | T | BT-20 | 629 |
| chr6 | 29364815 | C | G | HCC-2218BL | 635 |
| chr6 | 29364835 | T | G | HCC-2218BL | 641 |
| chr6 | 29364951 | G | A | HCC-2218 | 657 |
| chr3 | 75788484 | A | G | HCC-2218BL | 662 |
| chr16 | 71101200 | T | C | HCC-2218 | 673 |
| chr6 | 29555809 | C | T | BT-20 | 673 |
| chr6 | 29364787 | C | T | HCC-2218BL | 680 |
| chr6 | 29364787 | C | T | HCC-2218 | 692 |
| chr11 | 48366971 | G | C | MCF-7 | 696 |
| chr6 | 29364815 | C | G | HCC-2218 | 701 |
| chr8 | 18729817 | G | A | HCC-2218BL | 703 |
| chr6 | 29364835 | T | G | HCC-2218 | 707 |
| chr6 | 31084163 | A | G | HCC-2218 | 707 |
| chr16 | 71101200 | T | C | BT-20 | 728 |
| chr6 | 29555864 | C | T | HCC-2218 | 730 |
| chr20 | 6065729 | C | T | MCF-7 | 739 |
| chr11 | 48366971 | G | C | HCC-2218BL | 742 |
| chr16 | 71101200 | T | C | MCF-7 | 753 |
| chr6 | 29555864 | C | T | HCC-2218BL | 753 |
| chr1 | 114948282 | T | G | MCF-7 | 759 |
| chr6 | 29555809 | C | T | HCC-2218BL | 911 |
| chr6 | 29555809 | C | T | HCC-2218 | 933 |
| chr11 | 1017338 | C | A | HCC-2218 | 1063 |
| chr20 | 6065729 | C | T | BT-20 | 1137 |
| chr11 | 1017338 | C | A | HCC-2218BL | 1199 |
| chr16 | 71318577 | T | C | BT-20 | 1214 |
| chr3 | 75788434 | G | T | BT-20 | 1292 |
| chr3 | 75788434 | G | T | MCF-7 | 1453 |
| chr17 | 73487884 | A | C | HCC-2218 | 1489 |
| chr16 | 70975667 | T | C | HCC-2218BL | 1540 |
| chr16 | 70975667 | T | C | HCC-2218 | 1827 |
| chr16 | 70975667 | T | C | MCF-7 | 1965 |
| chr16 | 70975667 | T | C | BT-20 | 2008 |
| chr3 | 75788434 | G | T | HCC-2218BL | 2364 |
