# Supplementary material for: Evaluation of Hybridization Capture Versus Amplicon‐Based Methods for Whole‐Exome Sequencing
Source: Hum Mutat. 2015 Jul 15;36(9):903–14. doi: 10.1002/humu.22825 (PMC4832303; doi:10.1002/humu.22825)
Supplement: Supplementary file 1 — Figure S1 Supporting Table S1 | General sample features Supporting Table S7 | VarScan2 single‐sample SNVs from SureSelect in commonly targeted bases Supporting Table S8 | VarScan2 single‐sample SNVs from SeqCap in commonly targeted bases Supporting Table S9 | VarScan2 single‐sample SNVs from HaloPlex in commonly targeted bases Supporting Table S10 | GATK single‐sample SNVs from SureSelect in commonly targeted bases Supporting Table S10 | GATK single‐sample SNVs from SeqCap in commonly targeted bases Supporting Table S12 | GATK single‐sample SNVs from HaloPlex in commonly targeted bases Supporting Table S13 | MuTect single‐sample SNVs from SureSelect in commonly targeted bases Supporting Table S14 | MuTect single‐sample SNVs from SeqCap in commonly targeted bases Supporting Table S15 | MuTect single‐sample SNVs from HaloPlex in commonly targeted bases Supporting Table S16 | Ion Torrent Suite single‐sample SNVs from AmpliSeq in commonly targeted bases Supporting Table S20 | SureCall single‐sample SNVs from HaloPlex in commonly targeted bases Table S21 | Number/Percent of SNVs in the CCLE in commonly targeted regions called by each technology Supporting Table S22 | Reasons Different Technologies Missed CCLE Mutations in Commonly Targeted Regions Supporting Table S23 | Indels in Commonly Targeted Regions Supporting Table S24 | Comparison of CNV calling HCC‐2218 to HCC‐2218BL on VarScan2 and SNP array [file HUMU-36-903-s001.zip › humu22825-sup-0002-SuppMat.pdf]

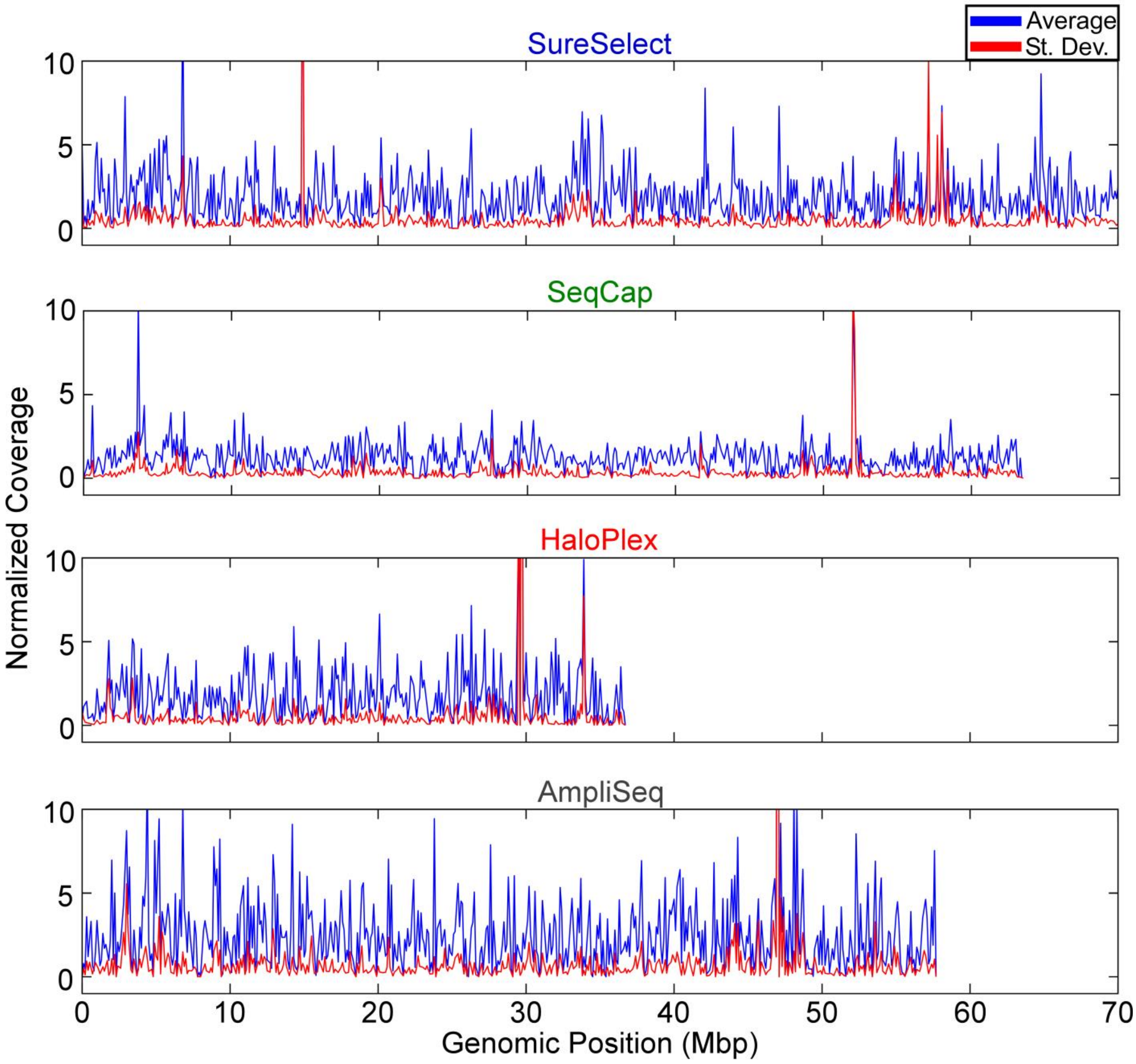

Supporting Figure S1. Normalized coverage in regions targeted specifically by each technology. For each technology's specific target region (X-axis), we calculated normalized coverage (reads per million sequenced reads) (Y-axis). The average (blue) and standard deviation (red) of normalized coverage are shown.

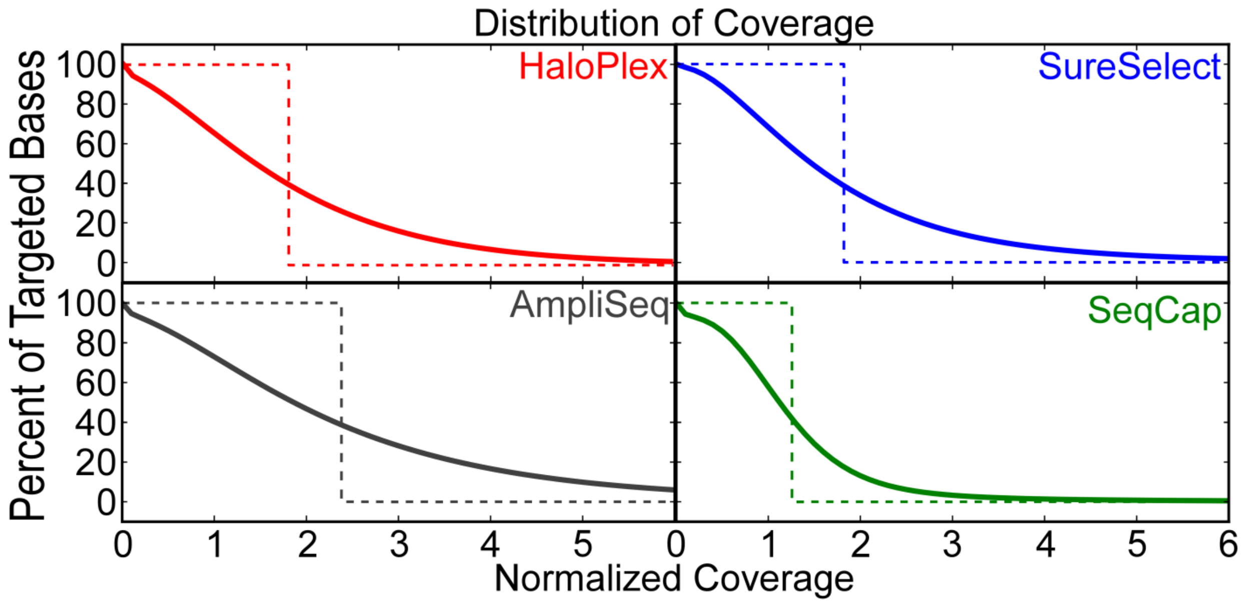

Supporting Figure S2. Distribution of coverage in each technology's targeted region. Percent of targeted bases in each technology's targeted region plotted against minimum normalized coverages (solid lines). Dotted lines are "ideal curves" representing distribution of coverage if each technology sequenced its target region with complete uniformity.

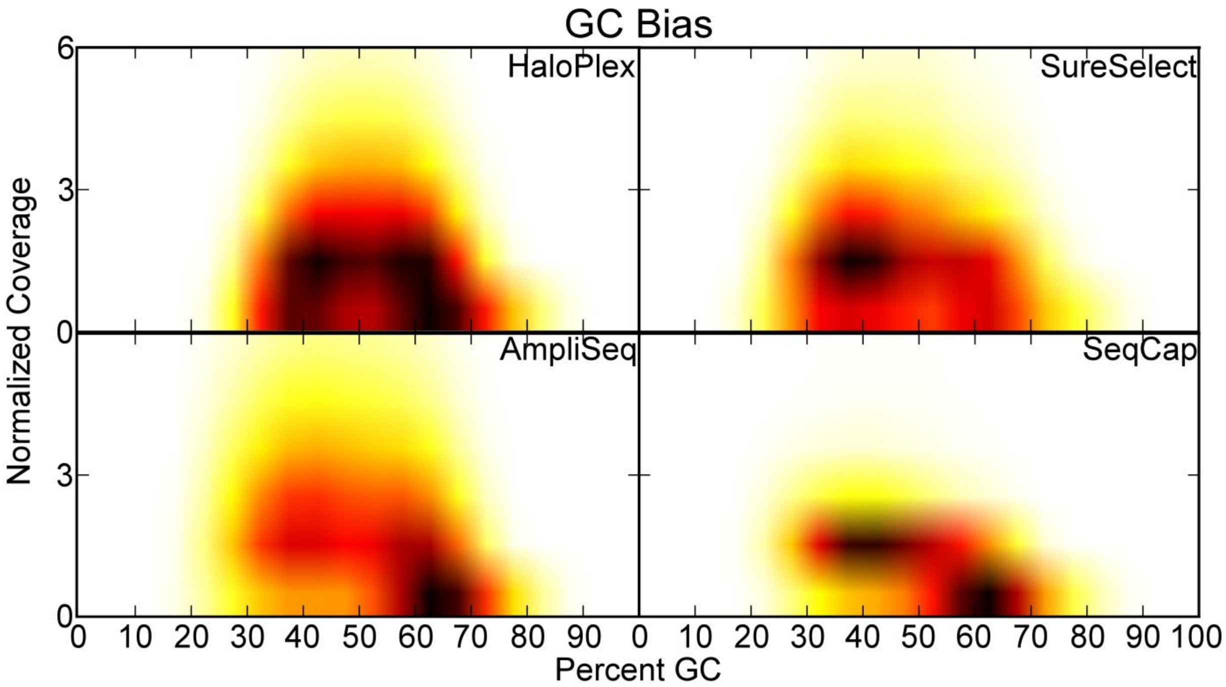

Supporting Figure S3. Capture performance based on GC-bias in each technology's target region. Percent-GC content was calculated in 100-bp windows for all bases in each technology's targeted region. Darker colors and brighter colors indicate denser and fewer clusters of points, respectively. Percent GC is plotted on the X-axis and normalized coverage (reads per million sequenced reads) is plotted on the Y-axis.

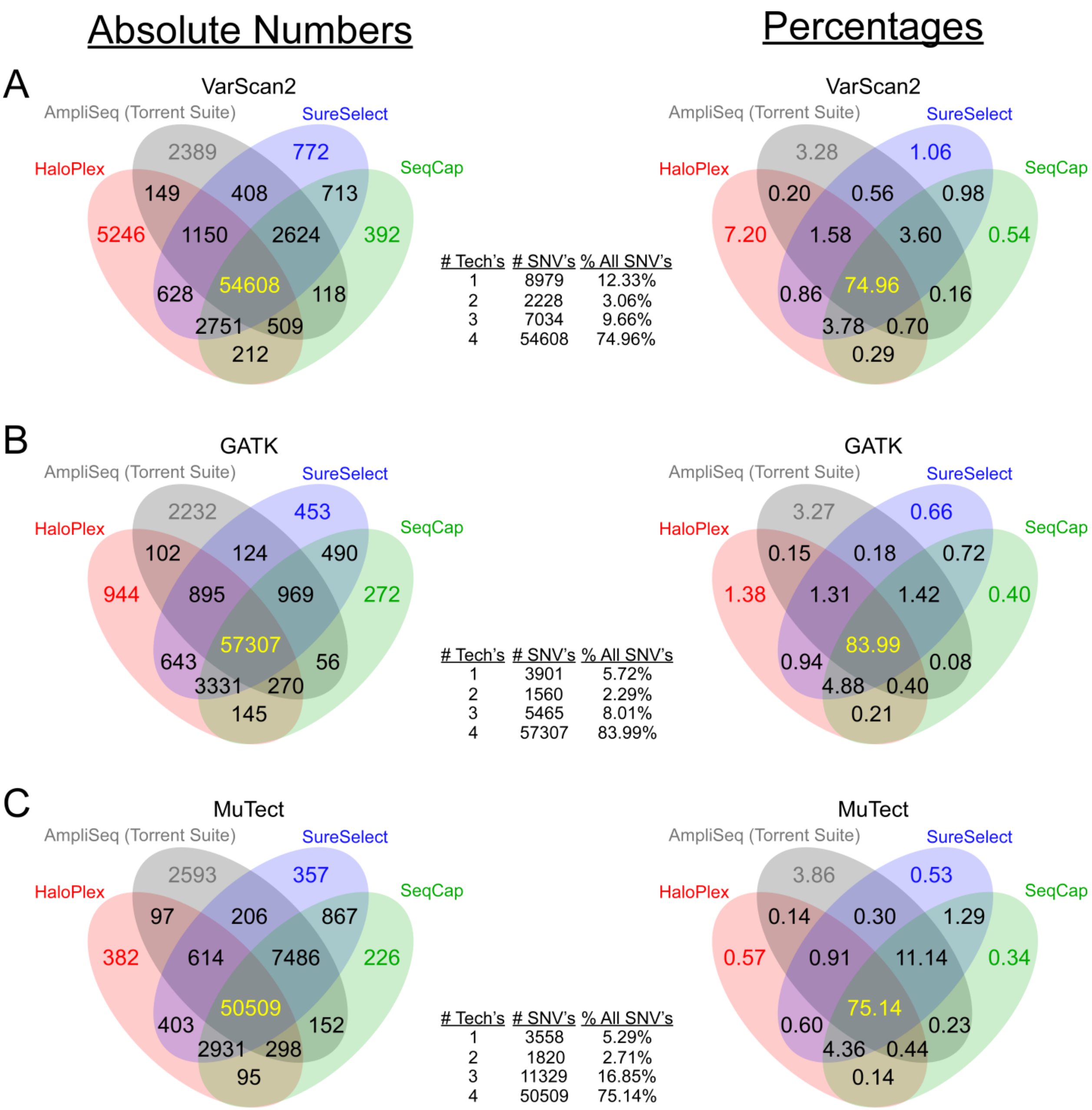

Supporting Figure S4. Concordance of SNV calling among technologies including both nonsynonymous and synonymous SNVs. Utilizing three variants callers, **A:** VarsScan2, **B:** GATK, and **C:** Mutect, Venn diagrams compare absolute numbers of nonsynonymous and synonymous SNVs (left) and percentage of the total number of nonsynonymous and synonymous SNVs (right) in commonly targeted regions. Tables in the center count the number and percentage of concordant SNVs called strictly in one, two, three, or all four technologies.

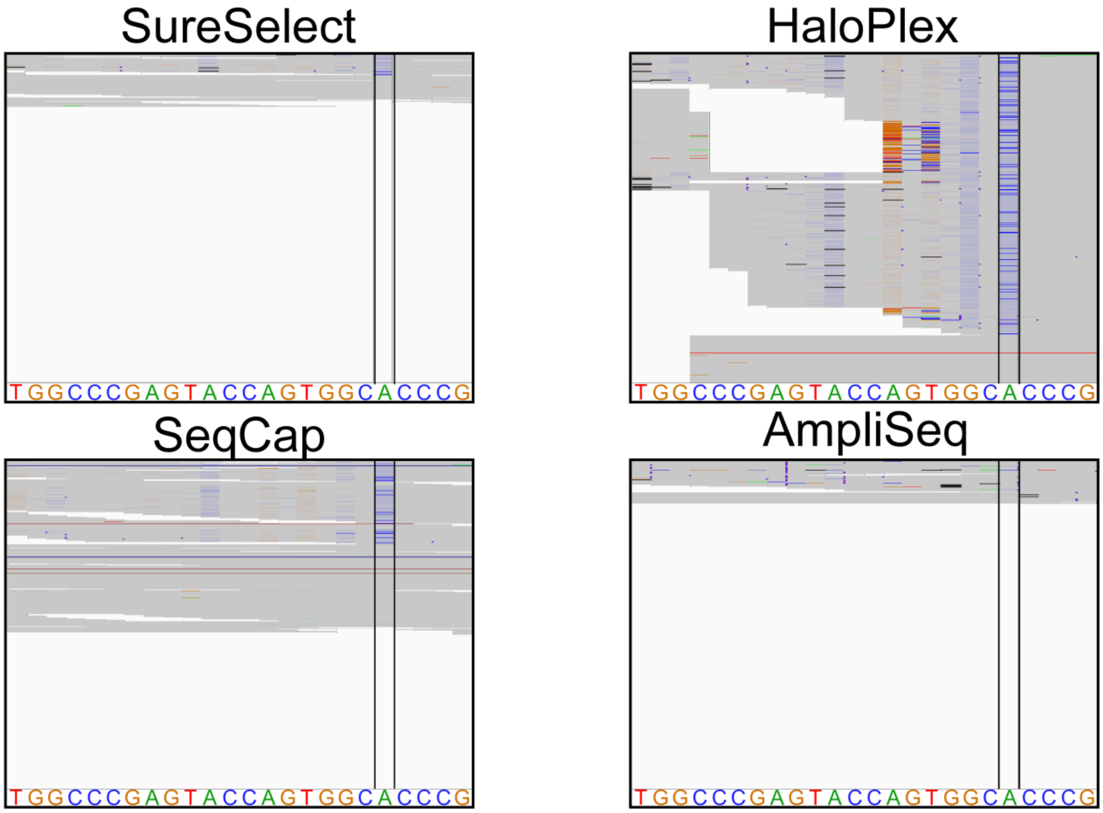

Supporting Figure S5. Illumina sequencing errors cause HaloPlex false-positive SNVs. Shown here is the MCF-7 cell line, chr17:61,561,867 A>C. GGT motifs on the negative strand (ACC motifs here on the positive strand) cause Illumina sequencing errors (Mechem, *et. al.* 2011, Nakamura, *et. al.* 2011). While these sequencing errors affect SureSelect, SeqCap, and HaloPlex, this SNV was only called in HaloPlex. Note that VarScan2 was used to call variant in SureSelect, SeqCap, and HaloPlex libraries and the Ion Torrent Suite on AmpliSeq libraries.

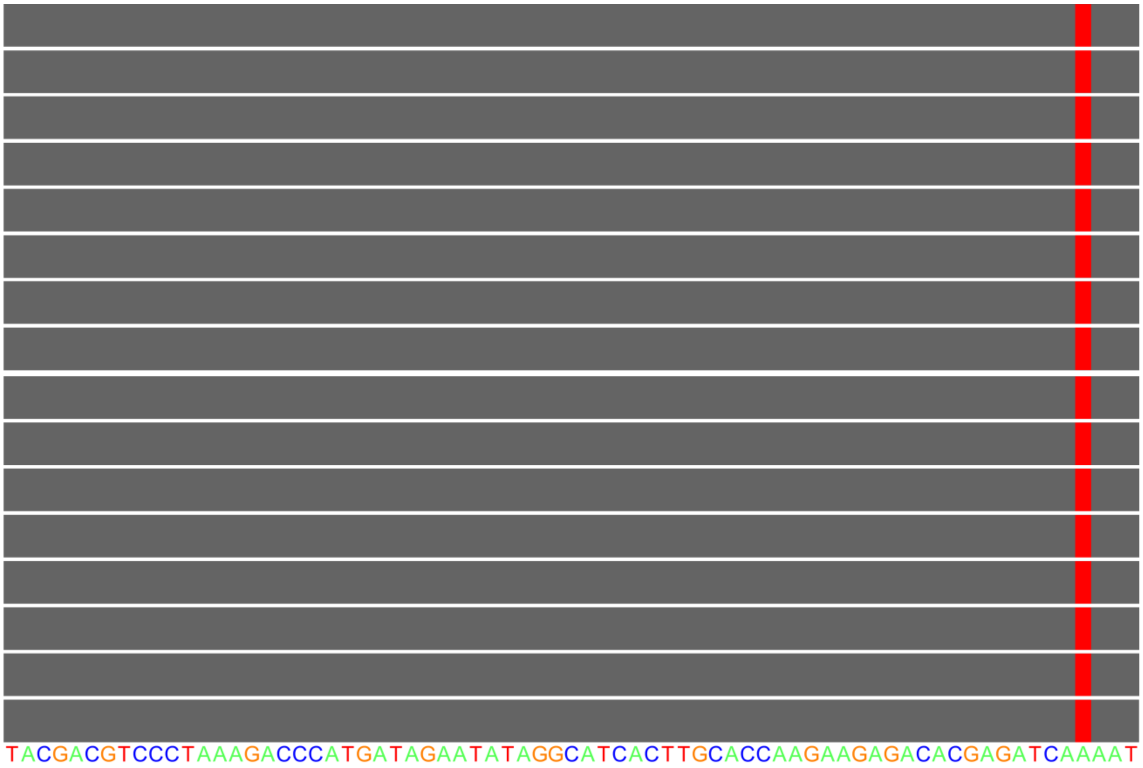

Supporting Figure S6. Clustered SNVs are rejected by MuTect. In this theoretical scenario, using a randomly generated DNA sequence, each gray bar represents an aligned read, while a red spot represents an an A>T SNV. When alternate alleles are consistently clustered near the start or end of a read, MuTect (Cibulskis, *et. al.* 2013) considers such a candidate SNV a false-positive, because of possible misalignments near the start or end of a read (Li, *et. al.* 2009b) and rejects the SNV, denoting it as a “clustered position.” This phenomenon was the main reason HaloPlex missed a large number of SNVs that other technologies called, when MuTect was used as the variant caller on SureSelect, SeqCap, and HaloPlex libraries and the Ion Torrent Suite on AmpliSeq libraries.

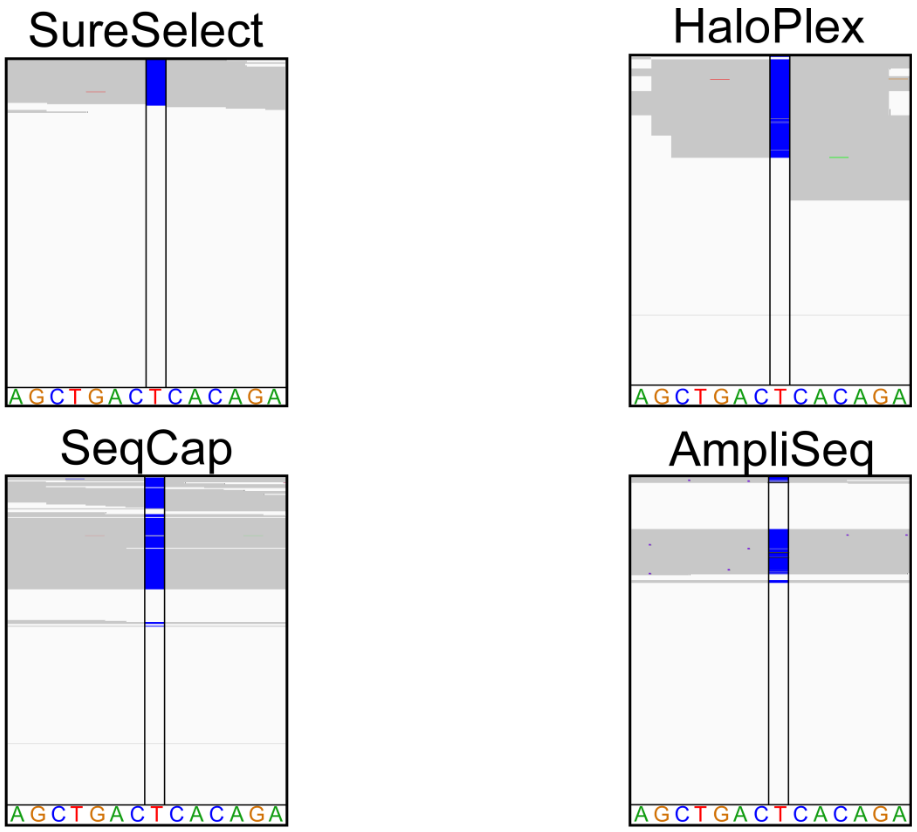

Supporting Figure S7. "Clustered position" SNV in HaloPlex libraries. Shown here is the BT-20 cell line, chr1:100,672,060 T>C. When MuTect was used to call variants in SureSelect, SeqCap, and HaloPlex libraries and the Ion Torrent Suite in AmpliSeq libraries, MuTect rejected this SNV in HaloPlex as a "clustered position," because most of the variant alleles were in close proximity to the start or end of a read. This was the main reason for HaloPlex to miss an SNV when MuTect was used the as the variant caller on SureSelect, SeqCap, and HaloPlex. The problem of "clustered position" is important in HaloPlex, because of its use of targeted restriction enzyme digestion, as opposed to random sonication used by SureSelect and SeqCap.

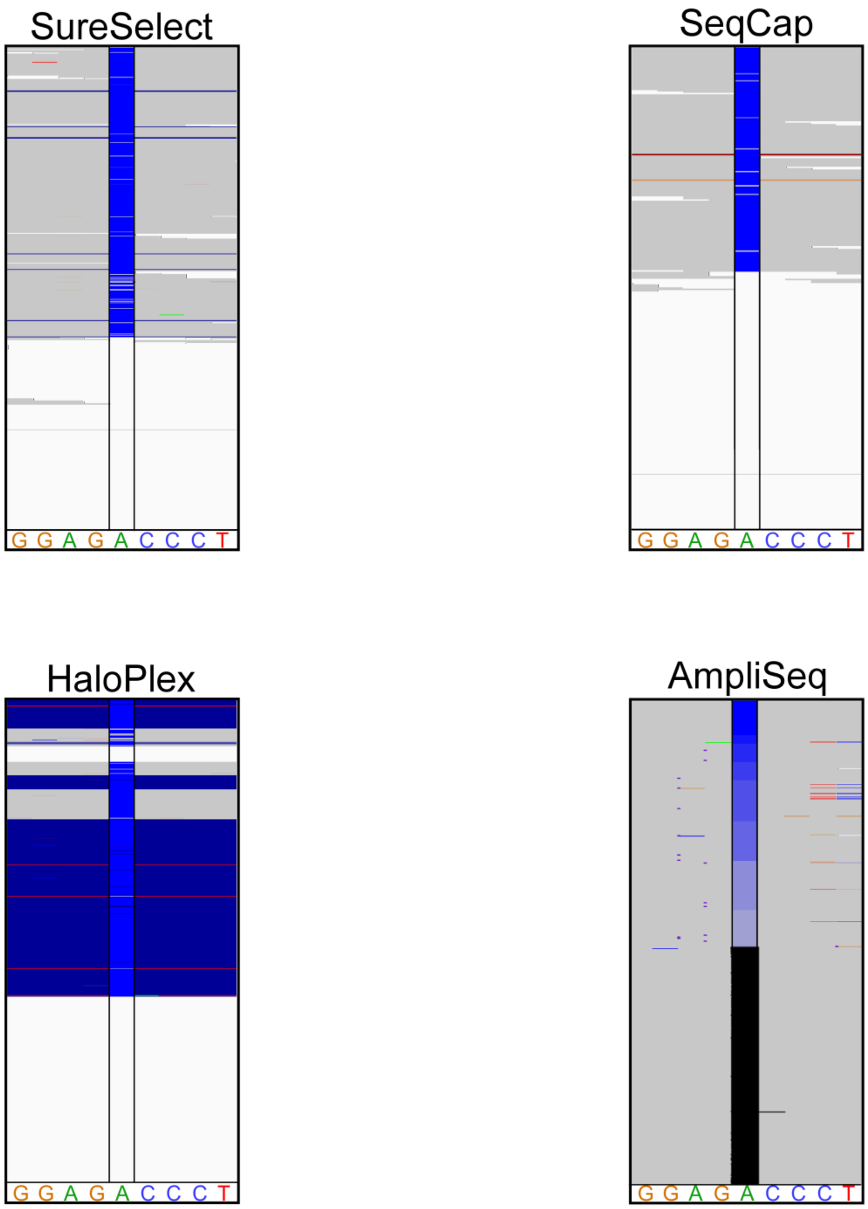

Supporting Figure S8. Ion Torrent sequencing errors cause false-negative SNVs in AmpliSeq. Shown is the MCF-7 cell line, chr5:163,260 A>C. Homopolymers of any length are known to cause Ion Torrent sequencing errors and result in erroneous measure of homopolymer length (Bragg, *et. al.* 2013). In this case, three C-nucleotides downstream of an A>C SNV generated deletion-supporting reads at the SNV, in turn, causing AmpliSeq (sequenced on the Ion Proton^{TM} System) to miss the SNV. Unlike AmpliSeq, the other technologies were sequenced on an Illumina machine, which is less prone to homopolymer sequencing errors, and caught the SNV.

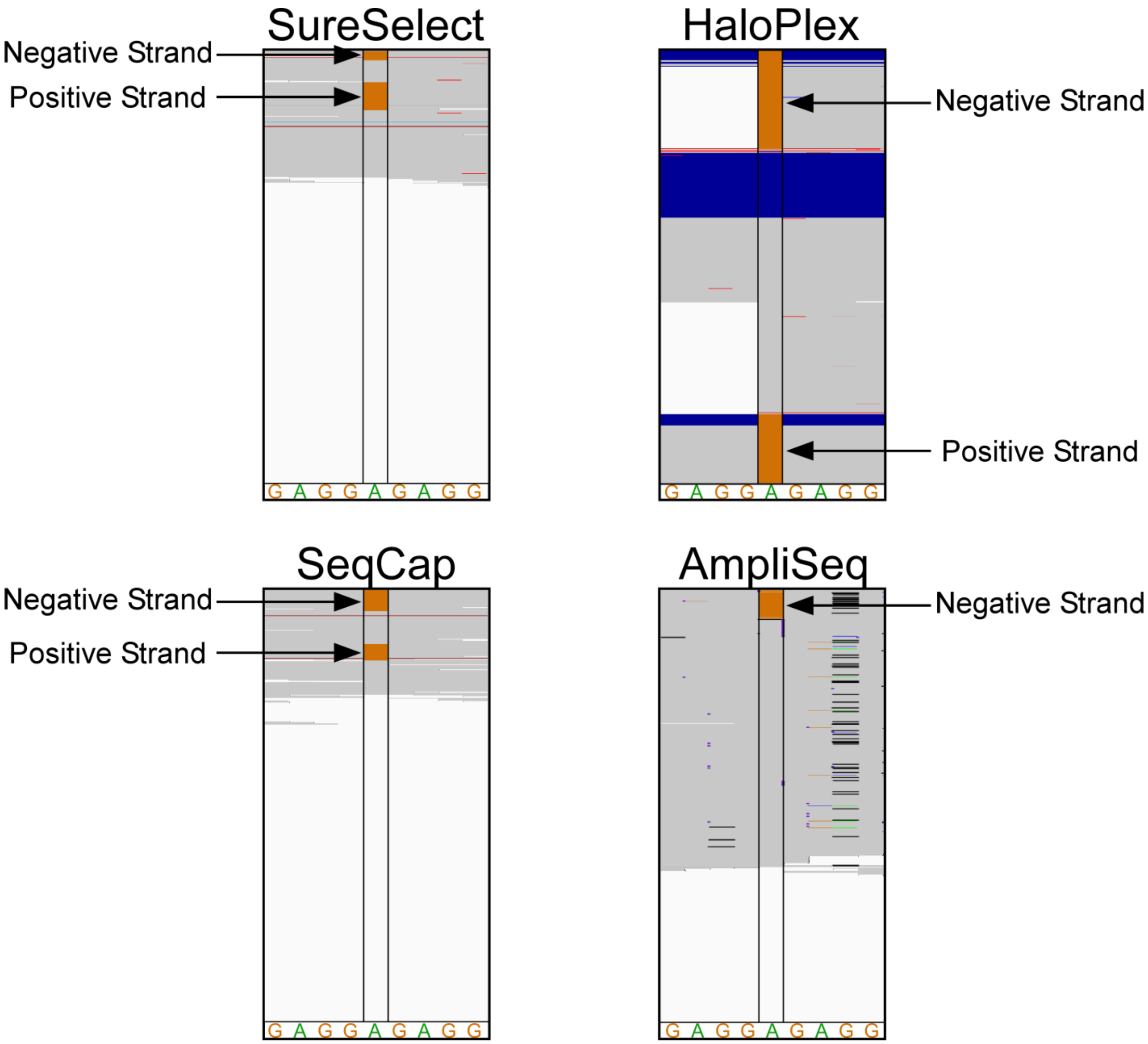

Supporting Figure S9. Strand bias causes the Ion Torrent Suite to reject an AmpliSeq SNV. Shown is the MCF-7 cell line, chr1:144,871,782 A>G. VarScan2 was used to call variants on the SureSelect, SeqCap, and HaloPlex libraries and the Ion Torrent Suite was used to call variants on the AmpliSeq library. Under the settings employed here, Ion Torrent Suite rejects SNVs that have at least 98% of variant-supporting reads on one strand. VarScan2, under the default parameters used here, disregards strand bias. In this figure, reads are sorted by strand and arrows point variant-supporting alleles on their respective strand.

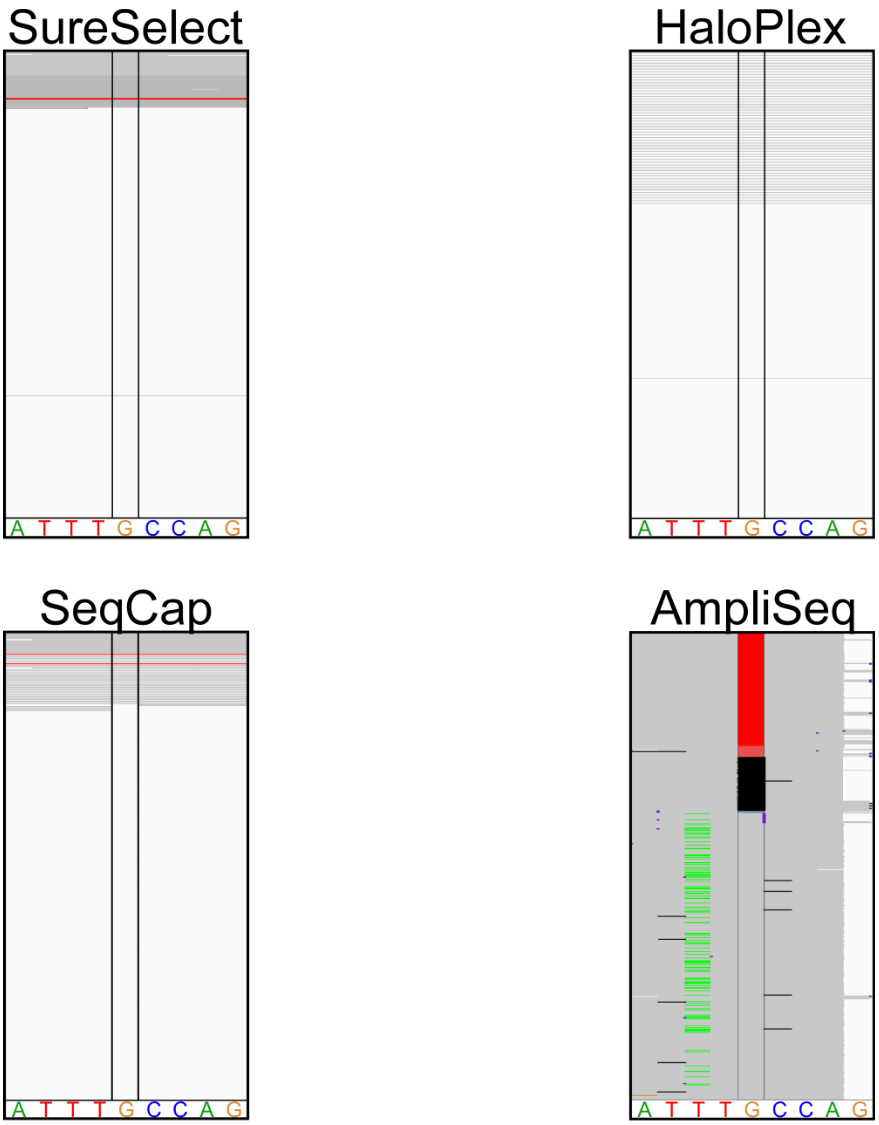

Supporting Figure S10. Ion Torrent sequencing errors cause false-positive SNVs in AmpliSeq. Shown is the MCF-7 cell line, chr3:75,788,434 G>T. Homopolymers are known to cause Ion Torrent sequencing errors resulting in erroneous measure of homopolymer length (Bragg, *et. al.* 2013). In this case, three T-nucleotides upstream of a G caused AmpliSeq (sequenced on the Ion Proton™ System) to call a false-positive G>T SNV. Unlike AmpliSeq, the other technologies were sequenced on an Illumina machine, which is less affected by homopolymers.

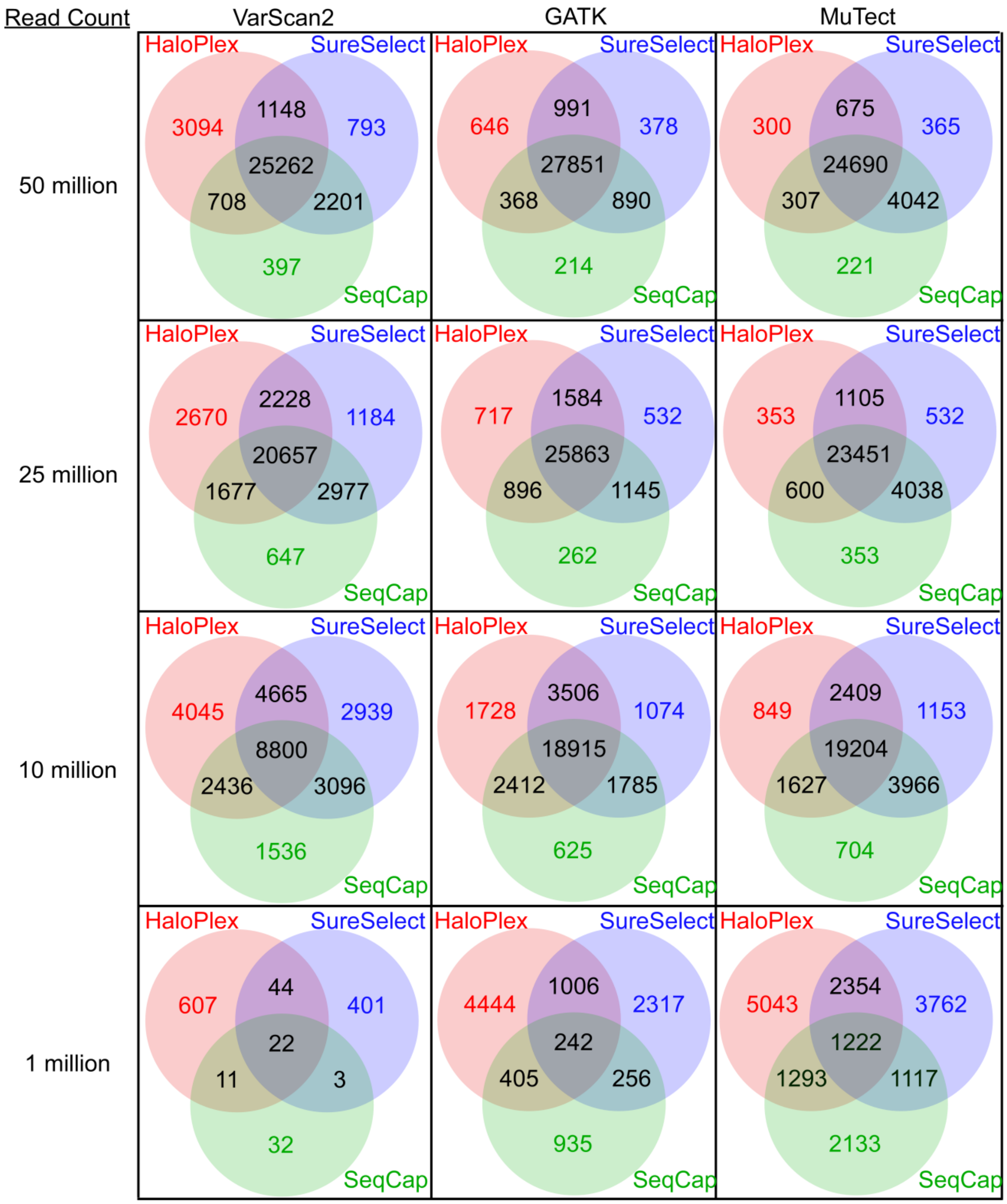

Supporting Figure S11. SNV concordance of technologies from downsampling. We downsampled all datasets to 1 million, 10 million, 25 million, and 50 million paired-end sequencing fragments. We aligned and post-processed the data as described in the Methods section and called nonsynonymous SNVs using VarScan2, GATK, and MuTect. Venn diagrams show concordance between technologies at these downsampled levels. We didn't calculate SNVs on downsampled AmpliSeq data, because we don't have access to the Ion Torrent Suite.

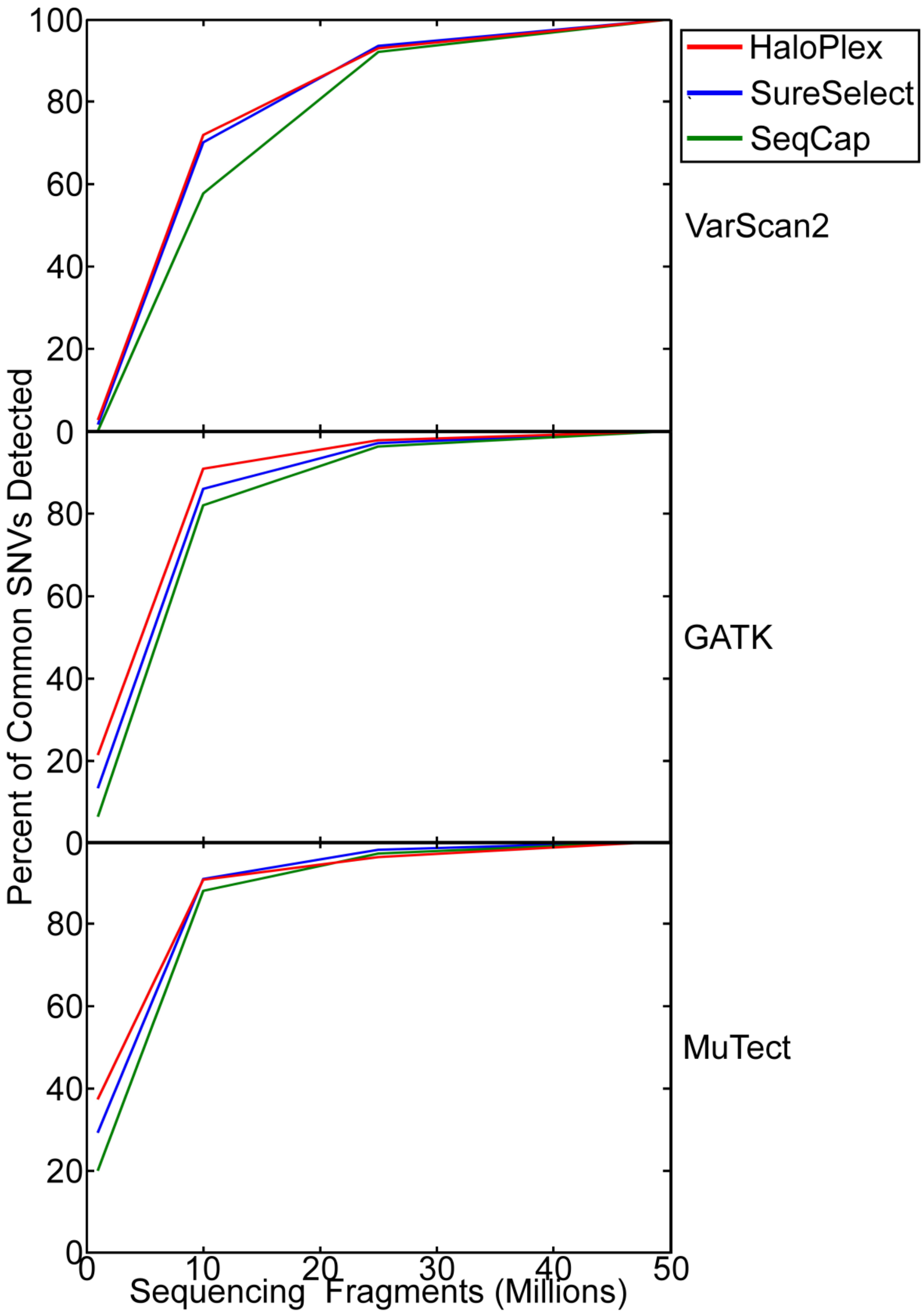

Supporting Figure S12. We calculated a common set of SNVs detected by all three technologies at 50 million fragments. Then, we downsampled to 25 million, 10 million, and 1 million fragments to determine the percentage of this common set of SNVs each technology detected at lower sequencing depths. HaloPlex captured the highest and SeqCap the lowest percentage of these SNVs.

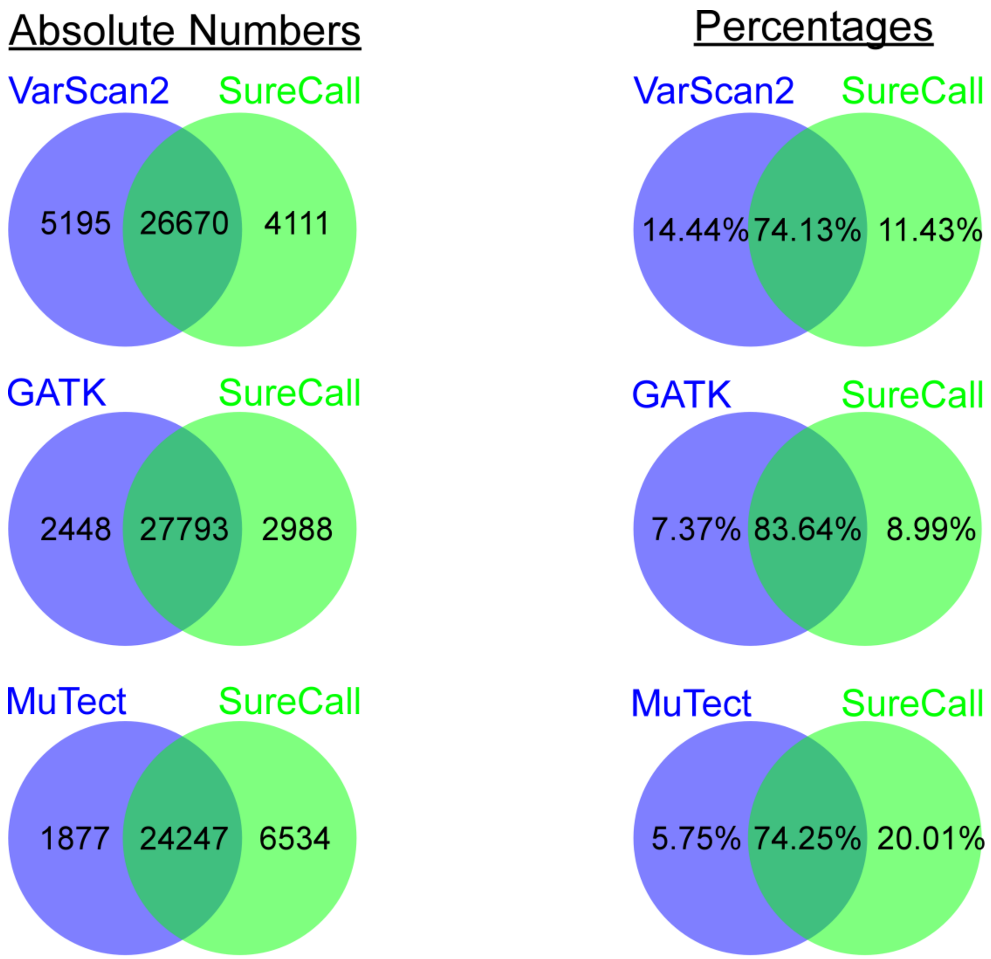

Supporting Figure S13. Concordance analysis with SureCall. We used Agilent's recommended analysis software for HaloPlex libraries, SureCall-2.1.1.13, to call SNVs on our HaloPlex data. We found good concordance of SureCall to VarScan2, GATK, and MuTect.

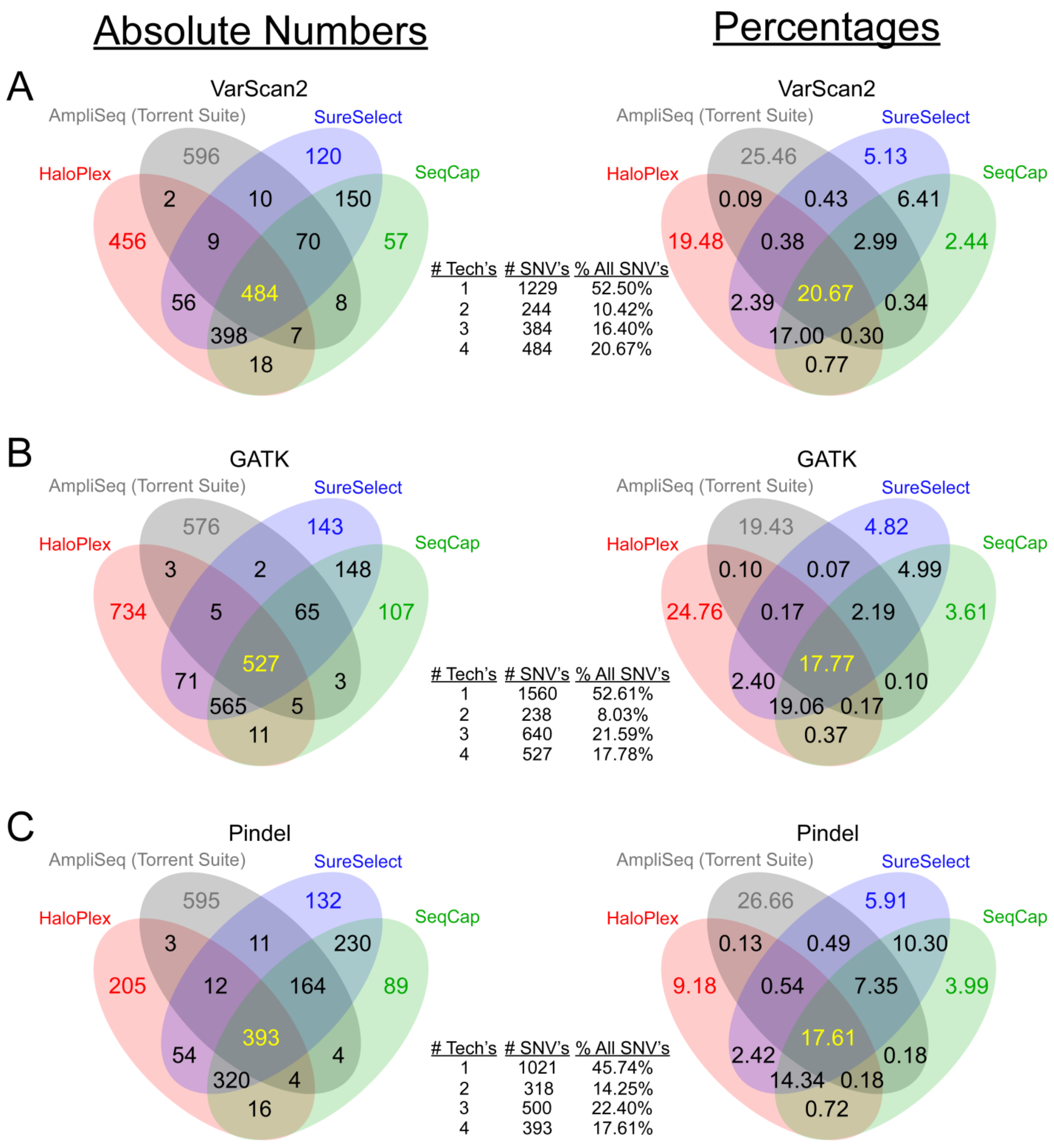

# Tech's	# SNV's	% All SNV's
1	1229	52.50%
2	244	10.42%
3	384	16.40%
4	484	20.67%

# Tech's	# SNV's	% All SNV's
1	1560	52.61%
2	238	8.03%
3	640	21.59%
4	527	17.78%

# Tech's	# SNV's	% All SNV's
1	1021	45.74%
2	318	14.25%
3	500	22.40%
4	393	17.61%

Supporting Figure S14. Concordance of indel calling among technologies. Employing three indel callers, **A:** VarScan2, **B:** GATK, and **C:** Pindel, Venn diagrams represent absolute number of indels (left) and percentage of total number of indels (right) in commonly-targeted regions. Tables in the center count the number of indels called by strictly one, two, three, or four technologies.

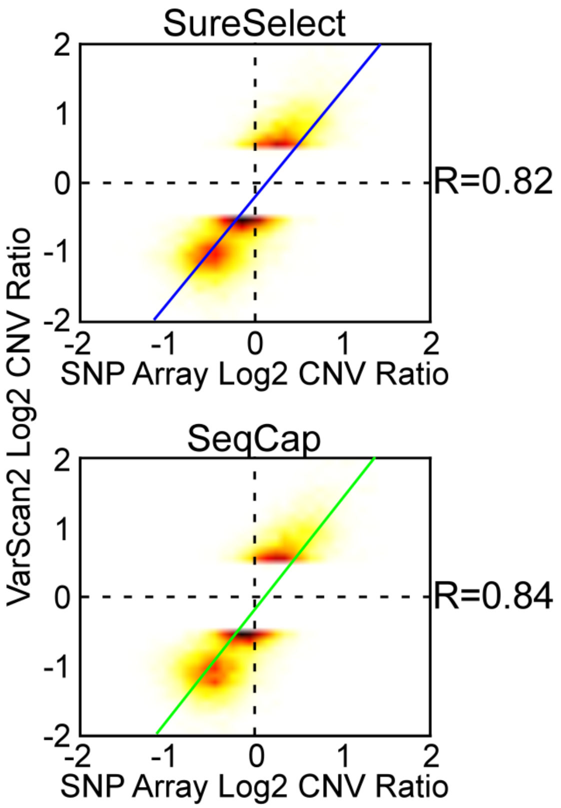

Supporting Figure S15. Correlation of exome sequencing-based CNV-calling to SNP array. VarScan2 was used to call CNVs on HCC-2218 using HCC-2218BL as a reference. For SureSelect, SeqCap, and HaloPlex, we used final BAMs and for AmpliSeq, we used the BAM generated immediately after alignment. Affymetrix's Genotyping Console™ software was used to make CNV calls on SNP 6.0 array data. Then, for each position in bases targeted by both the respective technology and the SNP array in the respective technology's targeted region. To ensure focus on high gain/loss regions, we excluded bases whose VarScan2 log2 CNV ratio was between -0.5 and 0.5. All technologies showed high correlation to the SNP array.

Supp. Tables S1, **S7-S16, and S20-S24** are available as separate Excel files under the Supporting Information for this article.

**Supp. Table S2. AmpliSeq settings**	
snp_min_allele_freq	0.1
snp_strand_bias	0.98
hotspot_min_coverage	6
hotspot_min_cov_each_strand	3
hotspot_min_allele_freq	0.1
snp_min_variant_score	15
hotspot_strand_bias	0.95
hp_max_length	8
filter_insertion_predictions	0.2
indel_min_variant_score	20
indel_min_coverage	10
heavy_tailed	3
outlier_probability	0.01
data_quality_stringency	5
snp_min_cov_each_strand	0
hotspot_min_variant_score	10
indel_strand_bias	0.9
tvc_parameters_version	germline_low_stringency_proton-3.6.66827
downsample_to_coverage	400
filter_unusual_predictions	0.25
indel_min_allele_freq	0.15
do_snp_realignment	1
prediction_precision	1
indel_min_cov_each_strand	5
filter_deletion_predictions	0.2
suppress_recalibration	0
snp_min_coverage	5

Supp. Table S3. P-values for all Mann-Whitney U tests

	Complexity			
	**SureSelect**	**SeqCap**	**HaloPlex**	**AmpliSeq**
**SureSelect**	-	0.03	0.03	0.03
**SeqCap**	-	-	0.03	0.03
**HaloPlex**	-	-	-	0.03
**AmpliSeq**	-	-	-	-

	Avg. Norm. Cov (common region)			
	**SureSelect**	**SeqCap**	**HaloPlex**	**AmpliSeq**
**SureSelect**	-	<1e-323	<1e-323	<1e-323
**SeqCap**	-	-	<1e-323	<1e-323
**HaloPlex**	-	-	-	<1e-323
**AmpliSeq**	-	-	-	-

	St. Dev. Norm. Cov tech (specific region)			
	**SureSelect**	**SeqCap**	**HaloPlex**	**AmpliSeq**
**SureSelect**	-	<1e-323	<1e-323	<1e-323
**SeqCap**	-	-	<1e-323	<1e-323
**HaloPlex**	-	-	-	<1e-323
**AmpliSeq**	-	-	-	-

	#CCLE Variants Detected			
	**SureSelect**	**SeqCap**	**HaloPlex**	**AmpliSeq**
**SureSelect**	-	0.56	0.45	0.93
**SeqCap**	-	-	0.17	1
**HaloPlex**	-	-	-	0.42
**AmpliSeq**	-	-	-	-

	Mapped to hg19			
	**SureSelect**	**SeqCap**	**HaloPlex**	**AmpliSeq**
**SureSelect**	-	0.03	0.03	0.02
**SeqCap**	-	-	0.03	0.02
**HaloPlex**	-	-	-	0.02
**AmpliSeq**	-	-	-	-

	Avg. Norm. Cov (tech specific region)			
	**SureSelect**	**SeqCap**	**HaloPlex**	**AmpliSeq**
**SureSelect**	-	<1e-	<1e-323	<1e-323

		323		
**SeqCap**	-	-	<1e-323	<1e-323
**HaloPlex**	-	-	-	<1e-323
**AmpliSeq**	-	-	-	-

	**Dev. from Ideal Curve (common region)**			
	**SureSelect**	**SeqCap**	**HaloPlex**	**AmpliSeq**
**SureSelect**	-	<1e-323	<1e-323	<1e-323
**SeqCap**	-	-	<1e-323	<1e-323
**HaloPlex**	-	-	-	<1e-323
**AmpliSeq**	-	-	-	-

	**hg19 to Targets**			
	**SureSelect**	**SeqCap**	**HaloPlex**	**AmpliSeq**
**SureSelect**	-	0.03	0.03	0.03
**SeqCap**	-	-	0.03	0.03
**HaloPlex**	-	-	-	0.03
**AmpliSeq**	-	-	-	-

	**St. Dev. Norm. Cov (common region)**			
	**SureSelect**	**SeqCap**	**HaloPlex**	**AmpliSeq**
**SureSelect**	-	<1e-323	<1e-323	<1e-323
**SeqCap**	-	-	<1e-323	<1e-323
**HaloPlex**	-	-	-	<1e-323
**AmpliSeq**	-	-	-	-

	**Dev. from Ideal Curve (tech-spec region)**			
	**SureSelect**	**SeqCap**	**HaloPlex**	**AmpliSeq**
**SureSelect**	-	<1e-323	<1e-323	<1e-323
**SeqCap**	-	-	<1e-323	<1e-323
**HaloPlex**	-	-	-	<1e-323
**AmpliSeq**	-	-	-	-

Supp. Table S4. Alignment rates

	SureSelect		
Sample	Pct. Fastq to Genome	Pct. Fastq to Target	Pct. Genome to Target
BT-20	91.27	77.4	84.81
MCF-7	91.99	78.05	84.84
H2218BL	91.32	78.2	85.63
H2218	91.86	78.17	85.1
Median	91.59	78.11	84.97
Med. Abs. Dev.	0.295	0.075	0.145

	SeqCap		
Sample	Pct. Fastq to Genome	Pct. Fastq to Target	Pct. Genome to Target
BT-20	95.12	77.3	81.27
MCF-7	94.99	63.2	66.53
H2218BL	94.14	75.56	80.26
H2218	93.77	74.22	79.15
Median	94.565	74.89	79.705
Med. Abs. Dev.	0.49	1.54	1.06

	HaloPlex		
Sample	Pct. Fastq to Genome	Pct. Fastq to Target	Pct. Genome to Target
BT-20	96.3	92.15	95.69
MCF-7	97.23	94.26	96.95
H2218BL	97.87	94.78	96.85
H2218	97.72	93.91	96.09
Median	97.475	94.085	96.47
Med. Abs. Dev.	0.32	0.435	0.43

	AmpliSeq		
Sample	Pct. Fastq to Genome	Pct. Fastq to Target	Pct. Genome to Target
BT-20	99.97	93.65	93.68
MCF-7	99.97	93.65	93.68

**H2218BL**	99.97	94.03	94.06
**H2218**	99.97	93.53	93.56
**Median**	99.97	93.65	93.68
**Med. Abs. Dev.**	0	0.06	0.06

Supp. Table S5. Average and st. dev. of normalized coverage in technology-specified regions and common regions (all units are reads per million sequenced reads)

	**Average**			
	**Common Regions**		**Technology-Specified Regions**	
	**Average**	**St. Dev.**	**Average**	**St. Dev.**
**SureSelect**	1.82	1.49	1.82	1.49
**SeqCap**	1.23	0.73	1.26	0.97
**HaloPlex**	1.85	1.56	1.8	1.56
**AmpliSeq**	2.54	2.26	2.38	2.19
	**St. Dev.**			
	**Common Regions**		**Technology-Specified Regions**	
	**Average**	**St. Dev.**	**Average**	**St. Dev.**
**SureSelect**	0.45	0.78	0.45	0.77
**SeqCap**	0.32	0.42	0.33	0.5
**HaloPlex**	0.47	0.88	0.46	0.85
**AmpliSeq**	0.69	1.23	0.65	1.18

Supp. Table S6. Deviations from ideal sequencing (all units are reads per million sequencing reads)

	Common Region		Tech-Spec Region	
	Average	St. Dev.	Average	St. Dev.
**SureSelect**	1.03	1.09	1.04	1.06
**SeqCap**	0.52	0.51	0.61	0.76
**HaloPlex**	1.12	1.08	1.12	1.08
**AmpliSeq**	1.59	1.6	1.54	1.56

Supp. Table S17. Classification of Discordant SNVs

	VarScan 2	GATK	MuTect
# Discordant SNVs	10510	6521	9126
# SNVs called only by one amplicon-based technology	5221	2315	1976
# SNVs missed only by one amplicon-based technology	2687	2305	5071
# SNVs called only by one hyb. capture-based technology	667	556	296
# SNVs missed only by one hyb. capture-based technology	723	460	428
% SNVs called or missed by only one amplicon-based technology	75.24263	70.84803	77.21893
% SNVs called or missed by only one hyb.capture -based technology	13.2255	15.58043	7.933377

Supp. Table S18. Variant Frequency of SNVs missed by only by HaloPlex when called under SureSelect and SeqCap (the variant caller is VarScan2)

Reason HaloPlex Missed SNV	Median SureSelect VF	Median SeqCap VF
Less than 8 reads with quality 15 at SNV position	100.00	100.00
VF < 20%	33.74	35.14
VF between 20% and 30%, but failed p-value	39.62	43.83
VF between 30% and 40%, but failed p-value	43.17	45.84
VF between 40% and 50%, but failed p-value	48.89	49.57
VF between 50% and 60%, but failed p-value	50.56	44.44
VF between 60% and 70%, but failed p-value	52.34	44.88

All these SNVs were missed by HaloPlex, but called by SureSelect, SeqCap, and AmpliSeq.
The caller for SureSelect, SeqCap, and HaloPlex was VarScan2.
The following SNVs met VarScan2's minimum raw coverage of 8 reads in HaloPlex.

Supp. Table S19. Potential false-negative and false-postive SNVs in HaloPlex and AmpliSeq that were investigated

SNVs called by HaloPlex, but missed by other technologies using VarScan2					
Chromosome	Position	Ref	Alt	Sample	Approx. Read Count
chr12	122812693	G	T	H2218BL	664
chr16	85704622	T	G	MCF-7	671
chr15	52613630	A	C	MCF-7	711
chr17	61561867	A	C	MCF-7	711
chr1	116579977	G	T	MCF-7	774
chr17	56581559	T	G	MCF-7	774
chr20	60895896	C	G	BT-20	795
chr7	55270348	T	C	BT-20	795
chr17	72745167	A	G	H2218	833
chr17	72745164	T	G	H2218	837
chr17	72745155	A	G	H2218	852
chr14	20404319	A	C	MCF-7	874
chr11	1017783	A	G	BT-20	965
chr20	47253062	T	C	MCF-7	1071
chr16	85704622	T	G	BT-20	1143
chr1	116579977	G	T	BT-20	1545
chr11	1017595	A	C	BT-20	1007
chr17	21319767	C	G	BT-20	698
chr11	1018419	G	A	H2218	1053
chr11	1018419	G	A	BT-20	1183
chr12	49724412	T	G	H2218	663
chr12	49724387	A	G	H2218	664
chr12	49724403	A	G	H2218	664
chr1	152323339	C	G	H2218	665
chr1	186277070	A	G	MCF-7	665
chr5	878525	T	A	BT-20	666
chr1	152382780	C	A	H2218	674
chr16	67320223	G	A	BT-20	697
chr8	101719201	A	G	MCF-7	700
chr12	49724304	G	A	H2218BL	702
chr6	112508770	G	T	BT-20	720
chr1	152323339	C	G	MCF-7	733
chr12	49724387	A	G	H2218BL	738
chr12	49724403	A	G	H2218BL	740
chr12	49724412	T	G	H2218BL	740
chr12	49724313	G	T	H2218BL	742
chr1	152382780	C	A	MCF-7	752

chr1	152382770	T	C	H2218	760
chr1	186277070	A	G	H2218	789
chr12	49724304	G	A	MCF-7	800
chr1	152382770	T	C	MCF-7	802
chr12	49724387	A	G	MCF-7	810
chr12	49724313	G	T	MCF-7	814
chr12	49724403	A	G	MCF-7	816
chr12	49724412	T	G	MCF-7	816
chr1	117944964	C	A	BT-20	824
chr1	152382802	T	G	H2218	839
chr1	152382802	T	G	MCF-7	924
chr5	173036394	C	T	BT-20	1201
chr20	56098733	T	C	MCF-7	1799

**SNVs missed by AmpliSeq, but called by other technologies using VarScan2**					
**Chromosome**	**Position**	**Ref**	**Alt**	**Sample**	**Approx. Read Count**
chr7	157931144	C	T	MCF-7	341
chr7	131128350	G	T	MCF-7	343
chr20	43723627	T	C	HCC-2218	350
chr11	48367097	A	G	BT-20	361
chr11	1017302	G	C	HCC-2218BL	365
chr3	89521693	T	C	HCC-2218BL	368
chr11	48373986	A	G	BT-20	371
chr9	37441650	T	C	BT-20	372
chr17	56621286	G	T	HCC-2218	375
chr7	157931144	C	T	BT-20	380
chr9	140147273	C	T	HCC-2218BL	385
chr11	85436868	G	C	MCF-7	387
chr15	90784207	A	G	HCC-2218	387
chr1	175129955	G	T	HCC-2218	393
chr15	99653800	T	C	BT-20	403
chr20	35769647	G	T	BT-20	408
chr9	140777306	C	G	BT-20	416
chr15	90784207	A	G	BT-20	422
chr3	183754294	G	C	BT-20	429
chr15	90784207	A	G	HCC-2218BL	431
chr1	144871782	A	G	MCF-7	435
chr15	90771750	T	G	HCC-2218BL	437
chr1	34071525	C	T	MCF-7	440
chr19	49469223	C	T	BT-20	440
chr1	12785494	G	T	HCC-2218BL	460

chr11	62381106	G	C	HCC-2218BL	471
chr3	183754278	C	G	BT-20	482
chr17	21319171	G	A	HCC-2218	487
chr1	145109661	G	A	HCC-2218BL	492
chr3	38793940	A	G	BT-20	508
chr6	57512529	C	A	HCC-2218BL	596
chr11	48367097	A	G	MCF-7	602
chr11	56468155	T	G	HCC-2218	615
chr11	56467945	G	T	HCC-2218BL	623
chr22	37462936	A	G	HCC-2218BL	625
chr15	90784207	A	G	MCF-7	639
chr5	163260	A	C	HCC-2218BL	674
chr16	70972595	T	C	MCF-7	681
chr11	48373885	A	G	HCC-2218	715
chr16	70972595	T	C	HCC-2218	751
chr11	48373986	A	G	HCC-2218	752
chr11	48373885	A	G	MCF-7	796
chr17	21319171	G	A	MCF-7	807
chr11	48373986	A	G	MCF-7	828
chr11	48373885	A	G	HCC-2218BL	856
chr11	48373986	A	G	HCC-2218BL	883
chr5	163260	A	C	MCF-7	888
chr5	163260	A	C	HCC-2218	1055
chr17	21319682	C	T	BT-20	1561
chr17	21319682	C	T	HCC-2218BL	1702

**SNVs called by AmpliSeq, but missed by other technologies using VarScan2**					
**Chromosome**	**Position**	**Ref**	**Alt**	**Sample**	**Approx. Read Count**
chr6	29364815	C	G	MCF-7	545
chr6	29364835	T	G	MCF-7	547
chr6	29364838	C	G	MCF-7	547
chrX	55172537	G	A	HCC-2218BL	549
chr6	29364787	C	T	MCF-7	557
chr6	31084163	A	G	HCC-2218BL	568
chr6	28963248	T	G	HCC-2218	577
chr17	73487884	A	C	MCF-7	580
chr6	29364787	C	T	BT-20	587
chr14	74060514	C	A	MCF-7	589
chr14	74060517	A	G	MCF-7	589
chr20	39832235	G	A	MCF-7	591
chr6	29364951	G	A	HCC-2218BL	603

chr17	21319007	G	A	HCC-2218	606
chr11	48366971	G	C	HCC-2218	621
chr3	105438956	C	T	BT-20	629
chr6	29364815	C	G	HCC-2218BL	635
chr6	29364835	T	G	HCC-2218BL	641
chr6	29364951	G	A	HCC-2218	657
chr3	75788484	A	G	HCC-2218BL	662
chr16	71101200	T	C	HCC-2218	673
chr6	29555809	C	T	BT-20	673
chr6	29364787	C	T	HCC-2218BL	680
chr6	29364787	C	T	HCC-2218	692
chr11	48366971	G	C	MCF-7	696
chr6	29364815	C	G	HCC-2218	701
chr8	18729817	G	A	HCC-2218BL	703
chr6	29364835	T	G	HCC-2218	707
chr6	31084163	A	G	HCC-2218	707
chr16	71101200	T	C	BT-20	728
chr6	29555864	C	T	HCC-2218	730
chr20	6065729	C	T	MCF-7	739
chr11	48366971	G	C	HCC-2218BL	742
chr16	71101200	T	C	MCF-7	753
chr6	29555864	C	T	HCC-2218BL	753
chr1	114948282	T	G	MCF-7	759
chr6	29555809	C	T	HCC-2218BL	911
chr6	29555809	C	T	HCC-2218	933
chr11	1017338	C	A	HCC-2218	1063
chr20	6065729	C	T	BT-20	1137
chr11	1017338	C	A	HCC-2218BL	1199
chr16	71318577	T	C	BT-20	1214
chr3	75788434	G	T	BT-20	1292
chr3	75788434	G	T	MCF-7	1453
chr17	73487884	A	C	HCC-2218	1489
chr16	70975667	T	C	HCC-2218BL	1540
chr16	70975667	T	C	HCC-2218	1827
chr16	70975667	T	C	MCF-7	1965
chr16	70975667	T	C	BT-20	2008
chr3	75788434	G	T	HCC-2218BL	2364
